# On the Converse of Pansu’s Theorem

**DOI:** 10.1007/s00205-024-02059-8

**Published:** 2024-12-10

**Authors:** Guido De Philippis, Andrea Marchese, Andrea Merlo, Andrea Pinamonti, Filip Rindler

**Affiliations:** 1 Courant Institute of Mathematical Sciences, New York University, 251 Mercer St., New York, NY 10012 USA; 2 Università di Trento, Via Sommarive 14, 38123 Trento, Italy; 3 Universidad del País Vasco (UPV/EHU), Barrio Sarriena S/N 48940, Leioa, Spain; 4 Mathematics Institute, University of Warwick, Coventry, CV4 7AL United Kingdom

**Keywords:** 26B05, 49Q15, 26A27, 28A75, 53C17

## Abstract

We provide a suitable generalisation of Pansu’s differentiability theorem to general Radon measures on Carnot groups and we show that if Lipschitz maps between Carnot groups are Pansu-differentiable almost everywhere for some Radon measures $\mu$, then $\mu$ must be absolutely continuous with respect to the Haar measure of the group.

## Introduction

Rademacher’s theorem asserts that Lipschitz functions defined on the Euclidean space are differentiable almost everywhere with respect to the Lebesgue measure. Obviously this result fails if the Lebesgue measure is replaced by an arbitrary measure, for instance a Dirac delta, so it is natural to ask whether this is a rigidity property of the Lebesgue measure, see [1–3, 46]. Namely, does there exist a singular measure for which Rademacher’s theorem holds? In [21], the first and the last author showed that the answer to the previous question is negative (in two dimensions, this also follows by combining the main result of [1, 2] with [3]). Such a result opened the road to a better understanding of the structure of Lipschitz differentiablility spaces, *RCD*(*K*, *N*) spaces, certain types of Sobolev spaces and also some general measures satisfying linear PDE constraints, see [11, 13, 15, 19, 20, 27, 33, 41].

In [18], Cheeger generalized Rademacher’s theorem to the setting of metric spaces endowed with a doubling measure and a Poincaré type inequality. This has inspired a lot of research in the area of analysis on metric measure spaces. The notion of Lipschitz differentiability space has been later axiomatised by Keith in [32]. In [16], Bate characterized Lipschitz differentiability spaces in terms of the existence of a sufficiently rich family of representations of the underlying measure as an integral of Lipschitz curve fragments.

Carnot groups are connected, simply connected, nilpotent Lie groups whose Lie algebra is stratified. Referring to the next section for more details, we only mention here that they are metric measure spaces whose ambient vector space is $\mathbb {R}^d$, the metric allowing movements only along certain horizontal curves, tangent to a given smooth non-involutive distribution of planes, the so-called first layer of the Lie algebra stratification. One can define a natural notion of differentiablity for functions between Carnot groups and a seminal theorem of Pansu, [43] , proves the analogue of Rademacher’s theorem in this setting. In particular, Carnot groups endowed with the Haar measure are Lipschitz differentiability spaces.

In this paper we prove the analogue in the Sub-Riemannian setting of the result proved in [21], namely that the Haar meaesure is indeed the (essentially) unique measure on a Carnot group, for which a Rademacher-type theorem can hold.

### Theorem 1.1

Let $\mathbb {G}$ be a Carnot group, $\mathbb {H}$ an homogeneous group, and let $\mu$ be a Radon measure on $\mathbb {G}$. If every Lipschitz function $f:\mathbb {G}\rightarrow \mathbb {H}$ is Pansu-differentiable $\mu$-almost everywhere in the sense of Definition 2.7, then $\mu$ is absolutely continuous with respect to the Haar measure on $\mathbb {G}$.

For the proof of Theorem 1.1 we refer to Theorem 7.6. In order to prove Theorem 1.1 we follow the same general strategy of its Euclidean counterpart. First we generalize the work of Alberti and the second author [3] by associating to every Radon measure $\mu$ on $\mathbb {G}$ a decomposability bundle $V(\mu ,\cdot )$, that identifies a set of directions along which a Rademacher-type theorem, adapted to the measure $\mu$ holds true; see Sect. 3. More precisely, we obtain the following result:

### Theorem 1.2

Let $\mu$ be a Radon measure on a Carnot group $\mathbb {G}$. Then, there exists a $\mu$-measurable family of homogeneous subgroups $V(\mu ,x)$ such that for every homogeneous group $\mathbb {H}$ and every Lipschitz function $f:\mathbb {G}\rightarrow \mathbb {H}$ is Pansu differentiable at $\mu$-almost every $x\in \mathbb {G}$ with respect to the $V(\mu ,x)$.

For the proof of Theorem 1.2 we refer to Theorem 6.6 and to Definition 2.7 for the introduction of the notion of differentiability along a homogeneous subgroup.

Once this bundle is obtained, we exploit the work of Bate [16] to show that for a measure $\mu$ satisfying the assumptions of Theorem 1.1, $V(\mu ,x)=\mathbb {G}$ for $\mu$-almost every *x*, see Proposition 7.4. Finally, we show that this forces $\mu$ to be absolutely continuous with respect to the Haar measure. This last step is obtained by a PDE-type argument that extends some of the result of [21] to the hypoelliptic setting.

We note that, although the general strategy follows the one used to prove the Euclidean counterpart of Theorem 1.1, its adaptation to the Carnot setting requires several non-trivial adjustments. In particular, one of the key step in the proof of the converse of Rademacher theorem is the link between the fact that the decomposability bundle of a measure has full dimension and the existence of a suitable family of normal currents, proved in [3, Section 6]. This is indeed a crucial point in order to rely on the results in [21]. The key geometric property used to show the existence of this family of currents is the fact that, given a compact set $K\subset [0,1]$ and a Lipschitz fragment $\gamma : K \rightarrow \mathbb {R}^n$ with $\gamma `(t)$ belonging to a cone $C$ for almost every $t \in K$, the fragment $\gamma$ mostly coincides, locally almost everywhere, with a Lipschitz curve $\tilde{\gamma }: (a,b)\subset [0,1] \rightarrow \mathbb {R}^n$, which still satisfies $\tilde{\gamma }`(t)\in C$, for almost every $t\in (a,b)$, see [3, (6.13)]. This property is in general false for Carnot groups, see [10] and [29], and it requires specific assumptions to be true [37, 45, 50, 53]. We need thus to rely on a completely different construction which we believe to be of independent interest, see Section 4.

The second key point is the extension of the theory established in [21] to the setting of differential operators defined by Hörmander type vector fields. Indeed, the results in [21] strongly rely on the notion of wave cone associated with a differential operator which is, loosely speaking, related to the notion of ellipticity. This notion is too strong in this context and it should be relaxed to the notion of hypoellipticity, which however is less "explicit". Luckily, for second order operators (which are the only ones needed in this context), this notion can be characterized algebraically and this allows to adapt the proofs in [21] to this setting, see Proposition 7.5. We conclude by noticing that it is an interesting question to extend the full results of [21] to a "hypoelliptic wave cone"; in this context also see the examples in [14].

Concerning application of the results obtained here, we mention the recent extension of Cheeger’s conjecture originally proved by the first, second and last author in [20] to the context of Pansu’s differentiability spaces by Antonelli, Le Donne, and the third-named author in [8].

## List of Notations

We add below a list of frequently used notations, together with the page of their first appearance:

**Table:** 

$|\cdot |$	Euclidean norm,	3
$d_c$	Carnot-Carathéodory metric	6
$\delta _\lambda$	Intrinsic dilations	3
$X_i$	Canonical horizontal vector fields	6
$\mathscr {M}(\mathbb {R}^n,\mathbb {R}^m)$	Family of vector-valued measures of finite mass endowed with the topology of weak* topology	3
$\mathbb {M}(\mu )$	Total mass of a real valued or vector-valued measure	3
*B*(*x*, *r*)	Ball of centre *x* and radius *r* with respect to the metric $d_c$	3
*U*(*x*, *r*)	Ball of centre *x* and radius *r* with respect to the Euclidean metric	3
$\textrm{Gr}(\mathbb {G})$	Grassmannian of homogeneous subgroups of $\mathbb {G}$	5
$\textrm{Gr}_\mathfrak {C}(\mathbb {G})$	Grassmannian of Carnot subgroups of $\mathbb {G}$	5
$V(\mu ,\cdot )$	Decomposability bundle of the Radon measure	11
$N(\mu ,\cdot )$	Auxiliary decomposability bundle of the Radon measure	21
$\partial T$	Boundary of a current *T*	8
$d_Vf(x)$	Differential of a Borel map *f* along the subgroup $V\in \textrm{Gr}(\mathbb {G})$	7

## Notation and Preliminaries

### Preliminaries on Carnot Groups

In this subsection we briefly introduce some notations on Carnot groups that we will extensively use throughout the paper. For a detailed account on Carnot and homogeneous groups we refer to [35].

A Carnot group $\mathbb {G}$ of step $\mathfrak s$ is a connected and simply connected Lie group whose Lie algebra $\mathfrak g$ admits a stratification $\mathfrak g=V_1\, \oplus \, V_2 \, \oplus \dots \oplus \, V_{\mathfrak {s}}$. We say that $V_1\, \oplus \, V_2 \, \oplus \dots \oplus \, V_\mathfrak {s}$ is a *stratification* of $\mathfrak g$ if $\mathfrak g = V_1\, \oplus \, V_2 \, \oplus \dots \oplus \, V_\mathfrak {s}$,

$$\begin{aligned} {[}V_1,V_i]=V_{i+1}, \quad \text {for every } i=1,\dots ,\mathfrak {s}-1, \quad \text {and} \quad {[}V_1,V_\mathfrak {s}]=\{0\}, \end{aligned}$$

where $[A,B]:=\textrm{span}\{[a,b]:a\in A,b\in B\}$. We call $V_1$ the *horizontal layer* of $\mathbb {G}$. We denote by *n* the topological dimension of $\mathfrak g$ and by $n_j$ the dimension of $V_j$ for every $j=1,\dots ,\mathfrak {s}$. Furthermore, we define $\pi _i:\mathfrak {g}\rightarrow V_i$ to be the projection maps on the *i*-th strata. We will often shorten the notation to $v_i:=\pi _iv$.

The exponential map $\exp :\mathfrak g \rightarrow \mathbb {G}$ is a global diffeomorphism from $\mathfrak g$ to $\mathbb {G}$. Hence, if we choose a basis $\{X_1,\dots , X_n\}$ of $\mathfrak g$, any $p\in \mathbb {G}$ can be written in a unique way as $p=\exp (p_1X_1+\dots +p_nX_n)$. This means that we can identify $p\in \mathbb {G}$ with the *n*-tuple $(p_1,\dots , p_n)\in \mathbb {R}^n$, $V_1$ with $\mathbb {R}^{n_1}$ and the group $\mathbb {G}$ itself with $\mathbb {R}^n$ endowed with $*$, the group operation determined by the Baker-Campbell-Hausdorff formula. *From now on, we will always assume that* $\mathbb {G}=(\mathbb {R}^n,*)$ *and, as a consequence, that the exponential map* $\exp$ *acts as the identity.* Further, for every $z\in \mathbb {G}$, we introduce the *left translations* $\tau _z:\mathbb {G}\rightarrow \mathbb {G}$ that are defined as $\tau _z(x):=z*x$. The stratification of $\mathfrak {g}$ carries with it a family of dilations $\delta _\lambda :\mathfrak {g}\rightarrow \mathfrak {g}$ of $\mathfrak {g}$ defined by

1 $$\begin{aligned} \delta _\lambda (v_1,\dots , v_\mathfrak {s}):={\left\{ \begin{array}{ll} (\lambda v_1,\lambda ^2 v_2,\dots , \lambda ^\mathfrak {s} v_\mathfrak {s}), \quad & \text {for every }\lambda >0,\\ (-|\lambda |v_1,-|\lambda |^2 v_2,\dots , -|\lambda |^\mathfrak {s} v_\mathfrak {s}), \quad & \text {for every }\lambda \le 0, \end{array}\right. } \end{aligned}$$

where $v_i\in V_i$. The stratification of the Lie algebra $\mathfrak {g}$ naturally induces a gradation on each of its homogeneous Lie sub-algebras $\mathfrak {h}$, i.e., a sub-algebra that is $\delta _{\lambda }$-invariant for every $\lambda >0$

2 $$\begin{aligned} \mathfrak {h}=(V_1\cap \mathfrak {h})\oplus \ldots (\oplus V_\mathfrak {s}\cap \mathfrak {h}). \end{aligned}$$

We say that $\mathfrak h=W_1\oplus \dots \oplus W_{\mathfrak {s}}$ is a *grading* of $\mathfrak h$ if $[W_i,W_j]\subseteq W_{i+j}$ for every $1\le i,j\le \mathfrak {s}$, where we mean that $W_\ell :=\{0\}$ for every $\ell > \mathfrak {s}$. Since the exponential map acts as the identity, the Lie algebra automorphisms $\{\delta _\lambda :\lambda >0\}$ are also group automorphisms of $\mathbb {G}$.

#### Remark 2.1

Let us note that the definition of dilations given in (2), is not the natural one for $\lambda \le 0$. The natural definition would be

$$\begin{aligned} \tilde{\delta }_\lambda (v_1,\dots , v_\mathfrak {s}):= (\lambda v_1,\lambda ^2 v_2,\dots , \lambda ^\mathfrak {s} v_\mathfrak {s}),\qquad \text {for every }\lambda \in \mathbb {R}. \end{aligned}$$

However, in this work and purely for notations reasons, that will come apparent especially in (6), it is convenient to define dilations as in (1).

#### Definition 2.1

A subgroup $\mathbb {V}$ of $\mathbb {G}$ is said to be *homogeneous* if it is a Lie subgroup of $\mathbb {G}$ that is invariant under the dilations $\delta _\lambda$ with $\lambda >0$. A homogeneous subgroup $\mathbb {V}\subset \mathbb {G}$ is called *horizontal subgroup* if $\mathbb {V}\subseteq \exp (V_1)=V_1$.

The following general fact will play a crucial role later on:

#### Proposition 2.1

Suppose *H* is a closed subgroup of $\mathbb {G}\cong (\mathbb {R}^n,*)$. Then *H* can be identified with a vector subspace of $\mathbb {R}^n$. In particular, homogeneous closed subgroups of $\mathbb {G}$ are in bijective correspondence through $\exp$ with the Lie sub-algebras of $\mathfrak {g}$ that are invariant under the dilations $\delta _\lambda$ with $\lambda >0$.

#### Proof

Thanks to [38, Theorem 3.6] we know that *H* is a Lie subgroup of $\mathbb {G}$. In particular its Lie algebra $\mathfrak {h}$ is a Lie sub-algebra of $\mathfrak {g}$. Thanks to the definition of the operation $*$, the exponential map $\textrm{exp}$ acts as the identity and thus *H*, can be identified with its Lie algebra in $\mathfrak {g}\cong \mathbb {R}^n$ and in particular it can be viewed as a vector subspace of $\mathbb {R}^n$. $\square$

*From now on, since* $\textrm{exp}$ *acts as the identity due to the choice of* $*$, *we will always identify the elements of* $\mathbb {G}$, *with their preimage under* $\textrm{exp}$ *in* $\mathfrak {g}$.

In what follows, if not stated otherwise, $\mathbb {G}$ will be a fixed Carnot group.

#### Definition 2.2

(Homogeneous left-invariant distance and norm) A metric $d:\mathbb {G}\times \mathbb {G}\rightarrow \mathbb {R}$ is said to be *homogeneous* and *left-invariant* if for every $x,y\in \mathbb {G}$ we have, respectively

(i) $$d(\delta _\lambda x,\delta _\lambda y)=\lambda d(x,y)$$ for every $$\lambda >0$$,
(ii) $d(z*x,z*y)=d(x,y)$ for every $z\in \mathbb {G}$.

Given a homogeneous left-invariant distance, its associated homogeneous norm is defined by $\Vert g\Vert _{d}:=d(g,0)$, for every $g\in \mathbb {G}$, where 0 is the identity element of $\mathbb {G}$. Given a homogeneous left-invariant distance *d* on $\mathbb {G}$, for every $x\in \mathbb {G}$ and every $E\subseteq \mathbb {G}$ we define $\textrm{dist}(x,E):=\inf \{d(x,y):y\in E\}$.

The specific choice of the metric is not relevant for our purposes thanks to the following result, [17, Proposition 5.1.4]. In the following we will leave the dependence of the norm on the metric always implicit:

#### Proposition 2.2

Assume $d_1,d_2$ are two homogeneous left-invariant metrics on $\mathbb {G}$. Then there exists a constant $C>0$ depending on $d_1$ and $d_2$ such that $C^{-1}d_1(x,y)\le d_2(x,y)\le Cd_1(x,y)$ for every $x,y\in \mathbb {G}$.

We refer to [40, Lemma 3.6] for the proof of the following result:

#### Lemma 2.3

For every left-invariant and homogeneous distance and for every $k>0$ there exists a constant $C_{1}:=C_{1}(k,\mathbb {G},d)>1$ such that if $x,y\in B(0,k)$, then

$$\begin{aligned} \Vert y^{-1}* x* y\Vert \le C_{1}\Vert x\Vert ^{1/\mathfrak {s}}. \end{aligned}$$

#### Remark 2.2

Let *d* be a left-invariant homogeneous distance on $\mathbb {G}$. It is well known, see for instance [17, Proposition 5.15.1], that for every compact subset *K* of $\mathbb {R}^n$ there is a constant $C(K,d)>1$ such that:

$$\begin{aligned} C(K,d)^{-1}|x-y|\le d(x,y)\le C(K,d)|x-y|^{1/\mathfrak {s}}\qquad \text {for every }x,y\in K, \end{aligned}$$

where $|\cdot |$ is the Euclidean norm. More precisely the constant *C* introduced above depends only on $\textrm{dist}(0,K)+\textrm{diam}(K)$ and *d*.

For every Lie algebra $\mathfrak {h}$ with stratification $\mathfrak h= W_1\oplus \ldots \oplus W_{\mathfrak {s}}$, we define its *homogeneous dimension* as

$$\begin{aligned} \text {dim}_{\textrm{hom}}(\mathfrak {h}):=\sum _{i=1}^{\mathfrak {s}} i\cdot \text {dim}(W_i). \end{aligned}$$

Thanks to (2) we infer that, if $\mathfrak {h}$ is a homogeneous Lie sub-algebra of $\mathfrak {g}$, then

$$\begin{aligned} \text {dim}_{\textrm{hom}}(\mathfrak {h})=\sum _{i=1}^{\mathfrak {s}} i\cdot \text {dim}(\mathfrak {h}\cap V_i). \end{aligned}$$

It is well-known that the Hausdorff dimension, of a graded Lie group $\mathbb {G}$ with respect to a left-invariant homogeneous distance coincides with the homogeneous dimension of its Lie algebra, see [36, Theorem 4.4].

#### Definition 2.3

(Carnot subgroups) Let $\Lambda \subset [0,\infty )$. Given a collection $\mathscr {F}=\{v_\lambda \in \mathbb {G}:\lambda \in \Lambda \}$ of elements of $\mathbb {G}$ we define the homogeneous subgroup $\mathfrak {S}(\mathscr {F})$ of $\mathbb {G}$ generated by $\mathscr {F}$ as

$$\begin{aligned} \mathfrak {S}(\mathscr {F}):= &   \textrm{cl}\big (\big \{\delta _{\rho _1}(v_{\lambda _1})*\cdots *\delta _{\rho _N}(v_{\lambda _N}):N\in \mathbb {N},\,\rho _j\in \mathbb {R}\text { and }\\  &   \quad \lambda _j\in \Lambda \text { for every every }j\in \{1,\ldots ,N\}\big \}\big ). \end{aligned}$$

We say that a subgroup *V* of $\mathbb {G}$ is a *Carnot subgroup* if $V=\mathfrak {S}(V\cap V_1)$.

#### Definition 2.4

(Intrinsic Grassmannian on Carnot groups) Let $\mathcal {Q}:=\text {dim}_{hom}(\mathfrak {g})$ and let $1\le h\le Q$. We define $\textrm{Gr}(h)$ and $\textrm{Gr}_{\mathfrak {C}}(h)$ to be the family of all homogeneous subgroups *W* of $\mathbb {G}$ with Hausdorff dimension *h* and the family of all Carnot subgroups *W* of $\mathbb {G}$ with Hausdorff dimension *h*,  respectively. Finally, we denote by $\textrm{Gr}(\mathbb {G})$ and $\textrm{Gr}_\mathfrak {C} (\mathbb {G})$ the sets

$$\begin{aligned} \textrm{Gr}(\mathbb {G})=\bigcup _{h=1}^Q \textrm{Gr}(h) and \textrm{Gr}_{\mathfrak {C}}(\mathbb {G})=\bigcup _{h=1}^Q \textrm{Gr}_{\mathfrak {C}}(h). \end{aligned}$$

Since it will be occasionally used, it will be convenient to denote by $\textrm{Gr}_{eu}(\mathbb {G})$ the Euclidean Grassmannian of the underlying space of $\mathbb {G}$ endowed with the topology generated by the Hausdorff distance induced by the Euclidean distance. It is easy to see that such topology and the one induced by the Carnot-Carathéodory Hausdorff distance are the same.

#### Proposition 2.4

Let $V\in \textrm{Gr}_\mathfrak {C}(\mathbb {G})$ and assume $v_1,\ldots ,v_N\in V\cap V_1$ are such that $V\cap V_1$ coincides with the linear span of $\{v_1,\ldots ,v_N\}$ when seen as vectors of $\mathbb {R}^n$. Then $\mathfrak {S}(\{v_1,\ldots ,v_N\})=V$.

#### Proof

The inclusion $\mathfrak {S}(\{v_1,\ldots ,v_N\})\subseteq V$ is obvious and thus we just need to prove the converse. Since $\mathfrak {S}(\{v_1,\ldots ,v_N\})$ is a closed homogeneous subgroup of $\mathbb {G}$, it is also a vector subspace of $\mathbb {G}$; see Proposition 2.1. Therefore, we have $\textrm{span}\{v_1,\ldots ,v_N\}=V\cap V_1\subseteq \mathfrak {S}(\{v_1,\ldots ,v_N\})$, and thus

$$\begin{aligned} V=\mathfrak {S}(V_1\cap V)\subseteq \mathfrak {S}(\{v_1,\ldots ,v_N\}), \end{aligned}$$

where the first identity follows from the fact that *V* is a Carnot subgroup of $\mathbb {G}$. $\square$

As already remarked above, we can suppose without loss of generality that the group operation $*$ is determined by the Campbell-Hausdorff formula. It is well known that $*$ has a polynomial expression in the coordinates, see [25, Proposition 2.1], and, more precisely,

$$\begin{aligned} p*q= p+q+\mathscr {Q}(p,q), \quad \text{ for } \text{ all } \, p,q \in \mathbb {R}^n, \end{aligned}$$

where $\mathscr {Q}=(\mathscr {Q}_1,\dots , \mathscr {Q}_{\mathfrak {s}}):\mathbb {R}^n\times \mathbb {R}^n \rightarrow V_1\oplus \ldots \oplus V_\mathfrak {s}$, and the $\mathscr {Q}_i$s are vector valued polynomials. For every $i=1,\ldots \mathfrak {s}$ and every $p,q\in \mathbb {G}$ we have

(i) $$\mathscr {Q}_i(\delta _\lambda p,\delta _\lambda q)=\lambda ^i\mathscr {Q}_i(p,q)$$ for $$\lambda >0$$,
(ii) $$\mathscr {Q}_i(p,q)=-\mathscr {Q}_i(-q,-p)$$,
(iii) $\mathscr {Q}_1=0$ and the polynomial $\mathscr {Q}_i$ depends only on the first $i-1$ components of *p* and *q*. Hence, we can write $\mathscr {Q}_i$ with abuse of notation as
  $$\begin{aligned} \mathscr {Q}_i(p,q)=\mathscr {Q}_i(p_1,\ldots ,p_{i-1},q_1,\ldots ,q_{i-1}). \end{aligned}$$

Therefore, we can represent the operation $*$ as

3 $$\begin{aligned} p * q= &   (p_1+q_1,p_2+q_2+\mathscr {Q}_2(p_1,q_1),\dots ,p_{\mathfrak {s}} +q_{\mathfrak {s}}\nonumber \\  &   +\mathscr {Q}_{\mathfrak {s}} (p_1,\dots , p_{\mathfrak s-1} ,q_1,\dots ,q_{\mathfrak s-1})). \end{aligned}$$

### Lipschitz Curves and the Horizontal Distribution and the Carnot-Carathéodory Distance

In this subsection we introduce the *horizontal distribution* of $n_1$-dimensional planes in $\mathbb {R}^n$ associated to $\mathbb {G}$ and we define the Carnot-Carathéodory distance.

#### Definition 2.5

Let $\{e_1,\ldots ,e_{n_1}\}$ be an orthonormal basis of $V_1$. For every $i=1,\ldots ,n_1$ we say that the left-invariant vector field tangent to $e_i$ at the origin,

4 $$\begin{aligned} X_i(x):=\lim _{t\rightarrow 0+}\frac{x*\delta _t(e_i)-x}{t}, \end{aligned}$$

is the *i*-th *horizontal vector field*. Furthermore, for every $i=1,\ldots ,n_1$ we can write the vector field $X_i$ as

$$\begin{aligned} X_i(x):=\sum _{j=1}^n \mathfrak {c}_j^i(x)\partial _j, \end{aligned}$$

where $\mathfrak {c}_j^i(x)$ are smooth functions since the $\mathscr {Q}_i$s are polynomial functions. In the following it will be useful to write the coefficients $\mathfrak {c}_j^i$ in the form of the matrix

$$\begin{aligned} \mathscr {C}(x):=\begin{pmatrix} \mathfrak {c}_1^1(x) & \dots &  \mathfrak {c}^{n_1}_1(x)\\ \vdots & \ddots & \vdots \\ \mathfrak {c}^1_n(x)& \dots & \mathfrak {c}^{n_1}_n(x) \end{pmatrix}. \end{aligned}$$

We further let

5 $$\begin{aligned} H\mathbb {G}(x):=\textrm{span}(X_1(x),\ldots ,X_{n_1}(x)). \end{aligned}$$

The distribution $H\mathbb {G}(x)$ of $n_1$-dimensional planes is usually said to be the *horizontal distribution* associated to the group $\mathbb {G}$.

#### Remark 2.3

(Expression for the $\mathfrak {c}_i$’s) Thanks to (4) and using the coordinate-wise expression of the operation $*$ given in (3), it is easy to see that

$$\begin{aligned} X_i(x)=e_i+\frac{\partial \mathscr {Q}}{\partial q_i}(x,0). \end{aligned}$$

This shows in particular that the matrix $\mathscr {C}(x)$ can be represented as

#### Definition 2.6

Let *B* be a bounded Borel subset of the real line. Given a map $\gamma :B\rightarrow \mathbb {G}$ and a Lebesgue density point $t\in B$ of $\gamma$, we denote that

$$\begin{aligned} \gamma ^\prime (t):=\lim _{\begin{array}{c} r\rightarrow 0 \\ t+r\in B \end{array}}\frac{\gamma (t+r)-\gamma (t)}{r},\qquad \text {whenever the right-hand side exists.} \end{aligned}$$

Furthermore, given $a<b$ we say that an absolutely continuous curve $\gamma :[a,b]\rightarrow \mathbb {G}$ is *horizontal* if there exists a measurable function $h: [a,b]\rightarrow V_1$ such that

(i) $\gamma ^\prime (t)=\mathscr {C}(\gamma (t))[h(t)]$ for $\mathscr {L}^1$-almost every $t\in [a,b]$,
(ii) $$|h|\in L^\infty ([a,b])$$.

Following the notation of [42] we shall refer to *h* as the *canonical coordinates of *$\gamma$ and if $\Vert h\Vert _\infty \le 1$ we will say that $\gamma$ is a *sub-unit* path. Finally, we define the Carnot-Carathéodory distance $d_c$ on $\mathbb {G}$ as

$$\begin{aligned} d_c(x,y):= &   \inf \{T\ge 0:\text { there is a sub-unit path }\gamma :[0,T]\rightarrow \mathbb {R}^n\text { such that }\\  &   \quad \gamma (0)=x\text { and }\gamma (T)=y\}. \end{aligned}$$

It is well known that $d_c(\cdot ,\cdot )$ is a left-invariant homogeneous metric on $\mathbb {G}$. Finally throughout the paper we will denote by $\Vert \cdot \Vert$ the homogeneous function $x\mapsto d_c(x,0)$ and *from now on and if not otherwise specified*, $\mathbb {G}$ *will always be endowed with the distance* $d_c$.

#### Proposition 2.5

The distance $d_c$ is a geodesic distance, i.e. for every $x,y\in \mathbb {G}$ there exists a sub-unit path $\gamma :[0,T]\rightarrow \mathbb {G}$ such that $\gamma (0)=x$, $\gamma (T)=y$ and $d_c(x,y)=T$.

#### Proof

This follows immediately from Proposition 2.2 and [26, Lemma 3.12]. $\square$

The following lemma allows us to characterise those Euclidean Lipschitz fragments that are also Lipschitz fragments when $\mathbb {R}^n$ is endowed with the Carnot-Carathéodory distance $d_c$ introduced above:

#### Lemma 2.6

Let *B* be a bounded Borel subset of the real line. If a map $\gamma :B\rightarrow \mathbb {G}$ is *L*-Lipschitz with respect to the distance $d_c$ on $\mathbb {G}$, then $\gamma$ is an Euclidean absolutely continuous map such that

$$\begin{aligned} \gamma ^\prime (t)=\mathscr {C}(\gamma (t))[h(t)]\qquad \text { for }\mathscr {L}^1\text {-almost every }t\in B \end{aligned}$$

for some $h\in L^\infty (B,V_1)$ with $\Vert h\Vert _\infty \le L$.

#### Remark 2.4

With abuse of language, for every Lipschitz fragment $\gamma :B\rightarrow \mathbb {G}$ we will refer to the function *h* yielded by Lemma 2.6 as *the canonical coordinates of* $\gamma$. For the original definition of canonical coordinates, see Definition 2.6.

#### Proof of Lemma 2.6

The proof of the lemma follows from [42, Lemma 1.3.3] together with an elementary localisation argument. $\square$

#### Definition 2.7

(Pansu differentiability) We say that a map $f:\mathbb {G}\rightarrow \mathbb {H}$ is Pansu differentiable at the point $x\in \mathbb {G}$ with respect to a homogeneous subgroup *V* of $\mathbb {G}$ if there exists a homogeneous homomorphism $L:V\rightarrow \mathbb {H}$ such that

$$\begin{aligned} d_{\mathbb {H}}\big (f(x)^{-1}*f(xh), L(h)\big )=o(\Vert h\Vert _{\mathbb {G}}) \quad \hbox {for all}\quad h\in V. \end{aligned}$$

When it exists, *L* is called the (Pansu) derivative of *f* at *x* with respect to *V* and is denoted by $d_V\hspace{-1.0pt}f(x)$. If $V=\mathbb {G}$ then $d_V\hspace{-1.0pt}f(x)$ is the usual (Pansu) derivative, and is simply denoted by *df*(*x*).

The next lemma can be proved with an immediate adaptation of the argument used to prove [42, Lemma 2.1.4] that allows us to characterise the Pansu derivative of Lipschitz fragments.

#### Lemma 2.7

Let *B* a bounded Borel subset of the real line and assume $\gamma :B\rightarrow \mathbb {G}$ is a Lipschitz fragment. If $h\in L^\infty (B,V_1)$ is the vector of canonical coordinates of $\gamma$, then for $\mathscr {L}^1$-almost every $t\in B$ we have:

$$\begin{aligned} D\gamma (t):=\lim _{\begin{array}{c} s\rightarrow 0+\\ t+s\in B \end{array}}\delta _{1/s}(\gamma (t)^{-1}*\gamma (t+s))=(h_1(t),\ldots ,h_{n_1}(t),0,\ldots ,0). \end{aligned}$$

In particular $D\gamma (t)$ exists for $\mathscr {L}^1$-almost every $t\in B$.

#### Proof

The proof of this lemma follows from [42, Lemma 2.1.4] together with an elementary localization argument. $\square$

#### Remark 2.5

Let us put ourselves in the notations of Lemma 2.7. It is useful to observe that Pansu’s differentiability theorem and the uniqueness of the limit imply that

$$\begin{aligned} \lim _{\begin{array}{c} s\rightarrow 0\\ t+s\in B \end{array}}\frac{d_\mathbb {G}(\gamma (t)^{-1}*\gamma (t+s),D\gamma (t)s)}{|s|}=0\qquad \text {for }\mathscr {L}^1\text {-almost every }t\in B, \end{aligned}$$

where here $D\gamma (t)s$ stands for the element $(s\,h_1(t),\ldots ,s\,h_{n_1}(t),0,\ldots ,0)$.

#### Definition 2.8

(*C*-curves) Let $e\in V_1$ be a unit vector and $\sigma \in (0,1)$. We denote by $C(e,\sigma )$ the one-sided, closed, convex cone with axis *e* and opening $\sigma$ in $V_1$, namely

$$\begin{aligned} C(e,\sigma ):=\{x\in V_1:\langle x,e\rangle \ge (1-\sigma ^2)|x|\}. \end{aligned}$$

Let *B* be a bounded Borel subset of the real line. A Lipschitz fragment $\gamma :B\rightarrow \mathbb {G}$, is said to be a $C(e,\sigma )$-fragment (or simply a *C*-fragment) if

6 $$\begin{aligned} \pi _1(\gamma (s))-\pi _1(\gamma (t))\in C(e,\sigma )\setminus \{0\}\text { for every }t,s\in B\text { with }t<s. \end{aligned}$$

If the domain of a $C(e,\sigma )$-fragment $\gamma$ is a compact interval, we will say that $\gamma$ is a $C(e,\sigma )$-curve (or simply a *C*-curve).

#### Proposition 2.8

Let *B* be a Borel subset of the real line and $\gamma :B\rightarrow \mathbb {G}$ be a Lipschitz map. Then, the measures  and  are mutually absolutely continuous.

#### Proof

Since $|x-y|\le d_c(x,y)$ for every $x,y\in \mathbb {G}$ the definition of Hausdorff measure immediately implies that $\mathscr {H}^1_{\textrm{eu}}\le \mathscr {H}^1$. For the converse, let us note that for every Lipschitz fragment $\gamma :B\rightarrow \mathbb {G}$ the area formula [39, Theorem 4.4] implies that for every Borel set $A\subseteq \mathbb {G}$ we havewhere the last identity follows from Lemma 2.4. This concludes the proof. $\square$

#### Remark 2.6

Thanks to Lemmas 2.6 and 2.7 if $\gamma :B\rightarrow \mathbb {G}$ is a *C*-fragment, then for $\mathscr {L}^1$-almost every $t\in B$ we have

$$\begin{aligned} (\pi _1\circ \gamma )^\prime (t)=\pi _1(\gamma ^\prime (t))=\pi _1(\mathscr {C}(x)[h(t)])=D\gamma (t), \end{aligned}$$

where *h* is the map of canonical coordinates associated to $\gamma$, see Remark 2.4.

#### Remark 2.7

Note that any $C(e,\sigma )$-fragment is injective. Indeed, if we suppose by contradiction that $\gamma (s)=\gamma (t)$ for some $t<s$ we would infer that $\pi _1(\gamma (s))=\pi _1(\gamma (t))$. This however is not possible thanks to (6).

In the next lemma, given a Lipschitz curve $\Gamma$ we construct a Borel map that at $\mathscr {H}^1$-almost every point of $\Gamma$, selects a vector that spans the tangent to the curve at that point.

#### Lemma 2.9

Let $\gamma :K\rightarrow \mathbb {G}$ be a Lipschitz fragment. Then, there exists a Borel map $\mathfrak {v}_\gamma :\mathbb {G}\rightarrow \mathbb {G}$ such that $\mathfrak {v}_\gamma (x)\in \{D\gamma (t):t\in \gamma ^{-1}(x)\}$ and $\mathfrak {v}_\gamma (x)\ne 0$ for -almost every $x\in \mathbb {G}$.

#### Proof

Just apply Lemma A.6 to the singleton measure family . $\square$

#### Definition 2.9

A Borel set $E\subset \mathbb {G}$ is called 1-*rectifiable* if there exists a countable family of Lipschitz maps $\gamma _i:K_i\rightarrow \mathbb {G}$, where $K_i$ are compact subsets of $\mathbb {R}$ such that $\mathscr {H}^1(E\setminus \bigcup _{i=1}^{\infty }\gamma _i(K_i))=0$. A Radon measure $\phi$ on $\mathbb {G}$ is said to be 1-rectifiable if there exists a 1-dimensional rectifiable set *E* such that .

### Euclidean and Horizontal Currents

We recall here the basic notions and terminology from the theory of Euclidean currents. A *k*-*dimensional current* (or *k*-current) in $\mathbb {R}^n$ is a continuous linear functional on the space of smooth and compactly supported differential *k*-forms on $\mathbb {R}^n$, endowed with the topology of test functions.

The boundary of a *k*-current $\textbf{T}$ is the $(k-1)$-current $\partial \textbf{T}$ defined by $\langle \partial \textbf{T}\, ; \, \omega \rangle := \langle \textbf{T}\, ; \, d\omega \rangle$ for every smooth and compactly supported $(k-1)$-form $\omega$ on $\mathbb {R}^n$, and where $d\omega$ denotes the exterior derivative of $\omega$. The *mass* of $\textbf{T}$, denoted by $\mathbb {M}(\textbf{T})$, is the supremum of $\langle \textbf{T}\, ; \, \omega \rangle$ over all forms $\omega$ such that $|\omega |\le 1$ everywhere. A current $\textbf{T}$ is called *normal* if both $\textbf{T}$ and $\partial \textbf{T}$ have finite mass.

By Riesz theorem a current $\textbf{T}$ with finite mass can be represented as a finite measure with values in the space  of *k*-vectors in $\mathbb {R}^n$, and therefore it can be written in the form $\textbf{T}=\tau \mu$ where $\mu$ is a finite positive measure and $\tau$ is a *k*-vector field such that $\int |\tau |d\mu < +\infty$. In particular the action of $\textbf{T}$ on a form $\omega$ is given by

$$\begin{aligned} \langle \textbf{T}\, ; \, \omega \rangle = \int _{\mathbb {R}^n} \langle \tau (x)\, ; \, \omega (x)\rangle \, d\mu (x) \, , \end{aligned}$$

and the mass $\mathbb {M}(\textbf{T})$ is the total mass of $\textbf{T}$ as a measure, that is, $\mathbb {M}(\textbf{T})=\int |\tau | d\mu$. Note that 0-dimensional currents with locally finite mass are signed Radon measures and the mass coincides with the total variation.

In the following, whenever we write a current $\textbf{T}$ as $\textbf{T}=\tau \mu$ we tacitly assume that $\tau (x)\ne 0$ for $\mu$-almost every  *x*; in this case we say that $\mu$ is a measure *associated* to the current $\textbf{T}$.

Moreover, if $\textbf{T}$ is a *k*-current with finite mass and $\mu$ is an arbitrary measure, we can write $\textbf{T}$ as $\textbf{T}=\tau \mu +\varvec{\nu }$ where $\tau$ is a *k*-vector field in $L^1(\mu )$, called the Radon-Nikodym density of $\textbf{T}$ w.r.t. $\mu$, and $\varvec{\nu }$ is a measure with values in *k*-vectors which is singular with respect to $\mu$.

Let $\mathbb {G}$ be a Carnot group. In the previous subsection we have already observed that $\mathbb {G}$ can be identified with $\mathbb {R}^n$, the underlying vector subspace of its Lie algebra, endowed with the operation given by the Baker-Campbell-Hausdorff formula. The 1-dimensional currents of finite mass in $\mathbb {R}^n$ that are of particular importance for this paper and for the geometry of $\mathbb {G}$ are those that are *tangent to the horizontal distribution of* $\mathbb {G}$ or simply *horizontal*.

#### Definition 2.10

(Horizontal 1-dimensional currents of finite mass) Let $\mathbb {G}=(\mathbb {R}^n,*)$. A 1-dimensional current of finite mass $\textbf{T}=\tau \mu$ on $\mathbb {R}^n$ is said to be $\mathbb {G}$-*horizontal*, or simply horizontal, if for $\mu$-almost every $x\in \mathbb {R}^n$ we have $\tau (x)\in H\mathbb {G}(x)$.

The following definition is a central concept throughout the paper, which is the integration of a family of measures:

#### Definition 2.11

(Integration of measures) Let (*I*, *dt*) be a ($\sigma$-)finite measure space and for every $t\in I$ let $\mu _t$ be a real- or vector-valued measure on $\mathbb {G}\cong \mathbb {R}^n$ such that

- for every Borel set *E* in $\mathbb {G}$ the function $t\mapsto \mu _t(E)$ is measurable;
- $$\int _I \mathbb {M}(\mu _t) \, dt <+\infty $$.

Then we denote by $\int _I \mu _t\, dt$ the measure on $\mathbb {G}$ defined by

$$\begin{aligned} {\textstyle \big [ \int _I \mu _t\, dt \big ]}(E) := \int _I \mu _t(E) \, dt \quad \text {for every Borel set }E\text { in }\mathbb {G}. \end{aligned}$$

Note that for every Borel set *E* in $\mathbb {G}$ the function $t\mapsto \mu _t(E)$ is measurable (Borel) if and only if $t\mapsto \mu _t$ is a measurable (Borel) map from *I* to the space of finite measures on $\mathbb {G}$ endowed with the weak* topology.

We now introduce some notation that will be used throughout the paper.

#### Definition 2.12

Let *B* be a Borel subset of $\mathbb {R}$ and $\gamma :B\rightarrow \mathbb {G}$ be a Lipschitz fragment. We denote by $\llbracket \gamma \rrbracket$ the current of finite mass that acts on compactly supported smooth 1-forms $\omega$ as

$$\begin{aligned} \langle \llbracket \gamma \rrbracket \, ; \, \omega \rangle :=\int _B \langle \gamma '(t)\, ; \, \omega (\gamma (t))\rangle dt. \end{aligned}$$

In the following it will be also useful to write , where $\rho$ is a suitable non-negative function in  and $\tau _\gamma (x)$ is a unitary Borel vector field that coincides with $\mathscr {C}(x)[\mathfrak {v}_\gamma (x)]$, up to a real (non-zero) multiple, -almost everywhere. For the definition of the vector field $\mathfrak {v}_\gamma$, see Lemma 2.9.

With this notation at hand we can introduce the following result (essentially due to Smirnov, see [49]):

#### Theorem 2.10

Let $\mathbb {G}$ be a Carnot group and let $\textbf{T}=\tau \mu$ be a 1-dimensional normal and horizontal current with $|\tau (x)|=1$ for $\mu$-almost every $x\in \mathbb {G}$. Then, there exists a family of vector-valued measures $t\mapsto \varvec{\mu }_t$ satisfying the hypothesis (a) and (b) of Definition 2.11 such that

(i) for almost every $t\in I$, where *I* is the real line with the Lebesgue measure $\mathscr {L}^1$, there exists a Lipschitz curve $\gamma _t:[0,1]\rightarrow \mathbb {G}$ for which $\varvec{\mu }_t=\llbracket \gamma _t\rrbracket$ and  for every smooth and compactly supported 1-form $\omega$;
(ii) if holds that  and, in particular, $\tau (x)=\tau _{\gamma _t}(x)$ for $\mathscr {H}^1$-almost every $x\in \textrm{im}(\gamma _t)$ and for almost every $t\in I$;
(iii) the measure $\mu$ can be written as .

Further, one can also rewrite $\textbf{T}$ as7where $E_{t,s}:=\{x\in \textrm{im}(\gamma _t):\rho _t(x)\ge s\}$ and the map  satisfies the hypothesis (a) and (b) of Definition 2.11 relative to $I\times [0,\infty )$. In addition,
8with $\tau _{\gamma _t}(x)=\tau (x)$ for $\mathscr {H}^1$-almost every $x\in \textrm{im}(\gamma _t)\cap E_{t,s}$ and for almost every $(s,t)\in \mathbb {R}\times I$.

#### Proof

Thanks to [44, Theorem 3.1], there exists a family of vector-valued measures $t\mapsto \mu _t$ satisfying the hypothesis (a) and (b) of Definition 2.11 such that for $\mathscr {L}^1$-almost every $t\in [0,\mathbb {M}(T)]$ there exists a Lipschitz curve $\gamma _t:[0,1]\rightarrow \mathbb {G}$ such that $\varvec{\mu }_t=\llbracket \gamma _t\rrbracket$ and

9 $$\begin{aligned} \begin{aligned} {{\textbf {T}}}=\int _0^{\mathbb {M}( {{\textbf {T}}})}\llbracket \gamma _t\rrbracket dt\quad \text{ and }\quad \mathbb {M}( {{\textbf {T}}})=\int _0^{\mathbb {M}( {{\textbf {T}}})}\mathbb {M}(\llbracket \gamma _t\rrbracket ) dt =\int _0^{\mathbb {M}( {{\textbf {T}}})}\int _0^1|\gamma _t^\prime (s)|ds\, dt. \end{aligned}\nonumber \\ \end{aligned}$$

The proof of items (i), (ii) and (iii) can be obtained from the above discussion with the same argument used for [3, Theorem 5.5]. The only variation on [3, Theorem 5.5] is how to prove that the fragments $\gamma _t$ used to decompose the current are Lipschitz, where the codomain is endowed with the Carnot-Carathéodory metric. This can be obtained as follows. The argument in [3, Theorem 5.5] implies that for $\mathscr {H}^1$-almost every $x\in \textrm{im}(\gamma _t)$ and almost every $t\in I$ we have

$$\begin{aligned} H\mathbb {G}(x)\ni \tau (x)=\tau _{\gamma _t}(x), \end{aligned}$$

which coincides with $\mathfrak {v}_{\gamma _t}(x)$ up to real, non-zero multiples, for $\mathscr {H}^1$-almost every $x\in \textrm{im}(\gamma _t)$ and almost every $t\in I$, see for instance Definition 2.12. This implies thanks to [42, Proposition 1.3.3] that for -almost every $x\in \mathbb {R}^n$ and almost every *t* the curve $\gamma _t$ is horizontal and thus Lipschitz if seen as curve $\gamma _t:[0,1]\rightarrow \mathbb {G}$.

The proof of (7) and (8) can be obtained defining applying the Cavalieri formula writing . $\square$

#### Remark 2.8

Let $\textbf{T}$ be a horizontal normal current such that $\partial \textbf{T}=0$. Then, for every smooth compactly supported 1-form we have

10 $$\begin{aligned}  &   \langle \partial \textbf{T}; \omega \rangle =\int _{\mathbb {R}^n}\langle \tau (x);d\omega (x)\rangle d\mu (x)\nonumber \\  &   \quad =\int \langle \tilde{\tau }(x);\,d_H \omega (x)\rangle _{\mathbb {R}^{n_1}} d\mu (x)=\langle {\bar{\tau }} \mu \, ; \, d_H\omega \rangle , \end{aligned}$$

where $\langle \cdot \, ; \, \cdot \rangle _{\mathbb {R}^{n_1}}$ denotes the dual coupling in $\mathbb {R}^{n_1}$ (we will drop the subscript $\mathbb {R}^{n_1}$ in the scalar product in the first layer if not otherwise specified) and

11 $$\begin{aligned} \begin{aligned} \tilde{\tau }(x):=\sum _{i=1}^{n_1}\tau _i(x)\partial _i\qquad \text {and}\qquad d_H\omega (x):=\sum _{i=1}^{n_1} X_i\omega (x)dx_i, \end{aligned} \end{aligned}$$

where $\tau (x)=\sum _{i=1}^{n_1}\tau _i(x)X_i(x)$. It will be convenient in the following to view horizontal finite mass 1-dimensional currents as Radon measures $\textbf{T}\in \mathcal {M}(\mathbb {G},\mathbb {R}^{n_1})$ which acts by duality on vector-valued smooth function $\omega \in C^\infty (\mathbb {G},\mathbb {R}^{n_1})$.

## The Decomposability Bundle

In this section we introduce an intrinsic notion of decomposability bundle to the setting of Carnot groups and we prove some of its elementary properties.

### Proposition 3.1

([9, Proposition 2.3]) Fix $1\le h\le Q$. For every $W_1, W_2\in \textrm{Gr}(h)$ let

$$\begin{aligned} d_{\mathbb {G}}(W_1, W_2):=d_{\mathscr {H},\mathbb {G}}( W_1\cap B(0,1), W_2\cap B(0,1)), \end{aligned}$$

where $d_{\mathscr {H},\mathbb {G}}$ is the Hausdorff distance of sets induced by some homogenous left invariant distance *d* on $\mathbb {G}$. Then, $d_{\mathbb {G}}$ is a metric on $\textrm{Gr}(h)$. Moreover $(\textrm{Gr}(h),d_{\mathbb {G}})$ is a compact metric space for every $h\in \{1,\ldots ,Q\}$ and thus $(\textrm{Gr}(\mathbb {G}),d_{\mathbb {G}})$ is a compact metric space as well.

### Lemma 3.2

Let $\mathbb {G}$ be a Carnot group. Let $\mu$ be a Radon measure on $\mathbb {G}$ and let $\mathscr {G}$ be a family of Borel maps from $\mathbb {G}$ to $\textrm{Gr}(\mathbb {G})$ which is closed under countable intersection, in the sense that for every countable family $\{V_i\} \subset \mathscr {G}$ the map *V* defined by $V(x):=\cap _i V_i(x)$ for every $x\in \mathbb {G}$ belongs to $\mathscr {G}$.

Then $\mathscr {G}$ admits an element *V* which is $\mu$-minimal, in the sense that every other $V'\in \mathscr {G}$ satisfies $V(x) \subset V'(x)$ for $\mu$-almost every *x*. Moreover this $\mu$-minimal element is unique modulo equivalence $\mu$-almost everywhere.

### Proof

The proof of this lemma is identical to its Euclidean counterpart, see [3, Lemma 2.4]. $\square$

### Definition 3.1

Let $\mu$ be a Radon measure on $\mathbb {G}$, let $\mathscr {F}$ be a family of Borel vector fields on $\mathbb {G}$ and let $\mathscr {G}$ be the class of all Borel maps $V:\mathbb {G}\rightarrow \textrm{Gr}(\mathbb {G})$ such that, for every $\tau \in \mathscr {F}$ if holds that

$$\begin{aligned} \tau (x) \subseteq V(x) \quad \text {for }\mu \text { -almost every}~x. \end{aligned}$$

Since $\mathscr {G}$ is closed under countable intersection, see Proposition A.1, by Lemma 3.2 it admits a $\mu$-minimal element which is unique modulo equivalence $\mu$-almost everywhere. We call *any* of these minimal elements the $\mu$-*essential span* of $\mathscr {F}$.

### Definition 3.2

(Decomposability bundle) Let $\mathbb {G}$ be a Carnot group. Given a Radon measure $\mu$ on $\mathbb {G}$ we denote by $\mathscr {F}_{\mu }$ the class of all families of measures $\{\mu _t : t\in I\}$ where *I* is a measured space endowed with a $\sigma$-finite measure *dt* and

- each $\mu _t$ is the restriction of $\mathscr {H}^1$ to a 1-Lipschitz fragment $\gamma _t:K_t\rightarrow \mathbb {G}$ with $K_t\subset \mathbb {R}$ compact;
- the map $t\mapsto \mu _t$ satisfies the assumptions (a) and (b) in Definition 2.11;
- the measure $\int _I \mu _t \, dt$ is absolutely continuous with respect to $\mu$.

We denote by $\mathscr {G}_{\mu }$ the class of all Borel maps $V:\mathbb {G}\rightarrow \textrm{Gr}(\mathbb {G})$ such that for every  it holds that

12 $$\begin{aligned} \mathfrak {v}_{\gamma _t}(x)\in V(x) \quad \text {for }\mu _t\text { -almost every}~x\text { and almost every}~t\in I, \end{aligned}$$

where $\mathfrak {v}_{\gamma _t}$ was introduced in Lemma 2.9 and the map $(t,x)\mapsto \mathfrak {v}_{\gamma _t}(x)$ is Borel thanks to Lemma A.6. Since $\mathscr {G}_{\mu }$ is closed under countable intersection, by Lemma 3.2 it admits a $\mu$-minimal element. We call *any* of these minimal elements the *decomposability bundle of* $\mu$, and denote it by $x\mapsto V(\mu ,x)$.

### Remark 3.1

If we substitute (a) with

- (a*) each $\mu _t$ is absolutely continuous with respect to the restriction of $\mathscr {H}^1$ to a Lipschitz fragment $\gamma _t$ in $\mathbb {G}$,

the definition of decomposability bundle does not change. Let us denote with $V^*(\mu ,\cdot )$ the decomposability bundle that arises from the assumptions (a*), (b) and (c). The inclusion $V(\mu ,x)\subseteq V^*(\mu ,x)$ is immediately seen to hold $\mu$-almost everywhere. Therefore, we only need to prove the converse inclusion, i.e. that for every family of measures $\mu _t$ satisfying (a*), (b) and (c) we have that

13 $$\begin{aligned} \mathfrak {v}_{\gamma _t}(x)\in V(\mu ,x) \quad \text {for } \mu _t\text {-almost every}~x\text { and almost every}~t\in I. \end{aligned}$$

In order to see this, let $\gamma : K\rightarrow \mathbb {G}$ be a Lipschitz fragment and suppose $\mu$ is a finite measure on $\mathbb {G}$ such that . The Radon-Nikodym’s decomposition theorem implies that there exists a  such that . Let $A\subseteq \mathbb {G}$ be any Borel set and note that the map $t\mapsto \mathscr {H}^1(A\cap \{x:\rho (x)\ge t\})$ is monotone. Hence, the measures $\nu _t:=\mathscr {H}^1(A\cap \{x:\rho (x)\ge t\})$ satisfy the hypothesis (a) and (b) of Definition 2.11 and thus their integral $\tilde{\mu }:=\int _0^\infty \nu _t e^{-t}dt$ is well defined. It is an easy task to check that the measures $\mu$ and $\tilde{\mu }$ are mutually absolutely continuous.

Thus, let $\{\mu _t\}_{t\in I}$ be a family of measures satisfying the hypothesis (a*), (b) and (c). For every $t\in I$ and any $s\in [0,\infty )$ we denote by $\nu _{s,t}$ the measure . It is immediate to see that the measures $(s,t)\mapsto \nu _{s,t}$ satisfy item (a) and a standard argument shows that they also satisfy item (b). In addition, the above discussions proves that the measures $\int \int \nu _{s,t}e^{-s}dtds$ and $\int \mu _tdt$ are mutually absolutely continuous and thus the $\nu _{s,t}$ satisfy also (c). By definition of $V(\mu ,x)$ this implies that

14 $$\begin{aligned} \mathfrak {v}_{\gamma _t}(x)\in V(\mu ,x) \quad \text {for } \nu _{s,t}\text {-almost every}~x\text { and almost every}~(s,t)\in [0,\infty )\times I. \end{aligned}$$

In fact, again by the above discussion we know that $\int \nu _{s,t}e^{-s}ds$ and $\mu _t$ are mutually absolutely continuous, and thus from (14) we infer that (13) holds. This shows that $V^*(\mu ,\cdot )=V(\mu ,\cdot )$.

### Lemma 3.3

Let $\mu$ be a Radon measure on $\mathbb {G}$. Then, $V(\mu ,x)\in \textrm{Gr}_\mathfrak {C}(\mathbb {G})$ for $\mu$-almost every $x\in \mathbb {G}$. In other words $V(\mu ,x)$ coincides with the closed subgroup of $\mathbb {G}$ generated by $V_1\cap V(\mu ,x)$ for $\mu$-almost every $x\in \mathbb {G}$.

### Proof

Since $\mathfrak {v}_{\gamma _t}(x)\in V_1$ for $\mu _t$-almost every *x*, by definition of $V(\mu , x)$ we have that

$$\begin{aligned} \mathfrak {v}_{\gamma _t}(x)\in V(\mu , x)\cap V_1 \quad \text {for } \mu _t\text {-almost every}~x\text { and almost every}~t\in I. \end{aligned}$$

Furthermore since $\Psi (x):=\mathfrak {S}(V_1\cap V(\mu , x))$, the homogeneous subgroup generated by $V_1\cap V(\mu , x)$, is contained in $V(\mu , x)$ for every $x\in \mathbb {G}$, we just need to show that the map $\Psi$ is Borel and thus it is a competitor in the definition of $V(\mu , x)$. The map $x\mapsto \mathbb {H}V(x):=V_1\cap V(\mu ,x)$ is Borel measurable thanks to Proposition A.1 and hence, since every element *W* of $\textrm{Gr}_\mathfrak {C} (\mathbb {G})$ is uniquely determined by $W\cap V_1$ and $\mathfrak {S}(V_1\cap V(\mu , x)) \in \textrm{Gr}_\mathfrak {C} (\mathbb {G})$, we infer that for every closed set $C\subseteq \textrm{Gr}(\mathbb {G})$ we have

15 $$\begin{aligned} \Psi ^{-1}(C)= &   \Psi ^{-1}(C\cap \textrm{Gr}_\mathfrak {C} (\mathbb {G})) =\mathbb {H}V^{-1}(\{V_1\cap W\in \textrm{Gr}(V_1):W\in C\cap \textrm{Gr}_\mathfrak {C}(\mathbb {G})\})\nonumber \\= &   \mathbb {H}V^{-1}(\{V_1\cap W\in \textrm{Gr}(V_1):W\in C\}). \end{aligned}$$

Since *C* is closed, the set $\{V_1\cap W\in \textrm{Gr}(V_1):W\in C\}$ is easily proved to be closed. Finally, thanks to the Borelianity of $\mathbb {H}V$ and (15) we thus infer that $\mathfrak {S}(V_1\cap V(\mu , x))^{-1}(C)$ is Borel as well and the proof of the proposition is achieved. $\square$

### Definition 3.3

Let us fix a Radon measure $\mu$ on $\mathbb {G}$. For every element $F\in \mathscr {F}_{\mu }$ we consider the family of all Borel maps $V:\mathbb {G}\rightarrow \textrm{Gr}_\mathfrak {C}(\mathbb {G})$ for which (12) holds. Since this class by Proposition A.1 is closed by countable intersection, by Lemma 3.2 it admits a $\mu$-minimal element $\mathfrak {V}(\mu ,F,\cdot )$ that is unique modulo equivalence $\mu$-almost everywhere.

### Proposition 3.4

Let $\mu$ be a Radon measure on $\mathbb {G}$, then

(i) for every $F \in \mathscr {F}_{\mu }$ there holds $\mathfrak {V}(\mu ,F,x) \subseteq V(\mu ,x)$ for $\mu$-almost every *x*;
(ii) there exists $G \in \mathscr {F}_{\mu }$ such that $\mathfrak {V}(\mu , G, x) = V(\mu , x)$ for $\mu$-almost every *x*.

### Proof

The proof of the proposition is identical to its Euclidean counterpart, see [3, Proposition 2.8]. $\square$

### Proposition 3.5

Let $\mu$, $\mu '$ be Radon measures on $\mathbb {G}$. Then, the following statements hold:

(i) [strong locality principle] if $\mu ' \ll \mu$ then $V(\mu ', x) = V(\mu , x)$ for $\mu '$-almost every *x*. More generally, if $1_E \, \mu ' \ll \mu$ for some Borel set $E\subset \mathbb {G}$, then $V(\mu ', x) = V(\mu , x)$ for $\mu '$-almost every $x\in E$,
(ii) there exists a $G=\{\mu _t : t\in I\} \in \mathscr {F}_{\mu }$ such that for $\mu$-almost every *x* we have $\mathfrak {V}(\mu , G, x) = V(\mu , x)$ and

### Proof

The proof of (i) is identical to its Euclidean counterpart, see [3, Proposition 2.9]. In order to prove (ii) let $G\in \mathscr {F}_{\mu }$ be the family of measures given by Proposition 3.4 (ii). The Radon-Nikodym decomposition of  with respect to $\nu :=\int _I\mu _t dt$ yields a Borel set *E* such that  and $\nu (\mathbb {G}\setminus E)=0$. Observe that the choice of *E* implies that $\mu _t(\mathbb {G}\setminus E)=0$ for almost every $t\in I$. We need to prove that $\mu '(\mathbb {G}\setminus E)=0$. Assume by contradiction that this is not the case, and observe that by point (i) the family  satisfies thatfor $\mu '$-almost every $x\in \mathbb {G}\setminus E$. This contradicts the fact that $V(\mu ,x)\ne \{0\}$ for $\mu '$-almost every $x\in \mathbb {G}$. $\square$

### Proposition 3.6

Let $\mu$ be a Radon measure on $\mathbb {G}$ and let $\textbf{T}$ be an horizontal 1-dimensional normal current, see Definition 2.10. Then

16 $$\begin{aligned} \pi _1(\tau (x))\subseteq V(\mu ,x)\qquad \text {for }\mu \text {-almost every } x\in \mathbb {G}, \end{aligned}$$

where we write $\textbf{T}=\tau \mu +\varvec{\sigma }$, with $\mu$ and $\varvec{\sigma }$ being mutually singular, and $\tau$ is the Radon-Nikodym derivative of $\textbf{T}$ with respect to $\mu$.

### Remark 3.2

Note that if $\Vert \textbf{T}\Vert$ and $\mu$ are mutually singular, then $\tau =0$ for $\mu$-almost every $x\in \mathbb {G}$ and therefore the inclusion is trivially satisfied.

### Proof

Let $\textbf{T}=\tau '\mu '$ with $|\tau ^\prime (x)|=1$ for $\mu '$-almost every $x\in \mathbb {G}$ and let $\{\varvec{\mu }_t\}_{t\in I}$ be the family of rectifiable measures yielded by Theorem 2.10. Thanks to Theorem 2.10, we know that for every $t\in I$ we have $\varvec{\mu }_t=\llbracket \gamma _t\rrbracket$ and for almost every $t\in I$ and $\mathscr {H}^1$-almost every $x\in \textrm{im}(\gamma _t)$ we have

17 $$\begin{aligned} \tau '(x)=\tau _{\gamma _t}(x)=\lambda \mathscr {C}(x)[\mathfrak {v}_{\gamma _t}(x)]\qquad \text {for some }\lambda \ne 0. \end{aligned}$$

However, since $\mu '=\int _I\Vert \varvec{\mu }_t\Vert dt$, this implies by definition of decomposability bundle that

18 $$\begin{aligned} \pi _1(\tau '(x))\in V(\mu ',x)\qquad \text {for }\mu '\text {-almost every } x\in \mathbb {G}. \end{aligned}$$

In order to conclude the proof, we need to check that

$$\begin{aligned} \pi _1(\tau '(x))\in V(\mu ,x)\qquad \mu \text {-almost every }x\in \mathbb {G}. \end{aligned}$$

In order to do so, we just need to check that $\tau (x)/|\tau (x)|=\tau '(x)$ and $V(\mu ',x)=V(\mu ,x)$ for $\mu$-almost every $x\in \mathbb {G}$. To this end, write $\textbf{T}=\tau '\mu '=\tau \mu +\varvec{\sigma }$, where $\mu$ and $\varvec{\sigma }$ are mutually singular and where here the vector field $\tau$ is the Radon-Nikodym derivative of ${\textbf {T}}$ with respect to $\mu$ as in the statement of the proposition. Let *A* be a Borel set, yielded by the Radon-Nikodym theorem such that $\mu (A)=0$ and $\varvec{\sigma }(A^c)=0$ and denote by ${\bar{\tau }}$ a $\mu$-measurable representative $\tau$. Finally, let $E\subseteq A^c$ be the $\mu$-measurable where ${\bar{\tau }}\ne 0$. It is immediate to see that  and hence by the uniqueness of the polar decomposition, we infer that $\tau '(x)=\tau (x)/|\tau (x)|$ on $\mu$-almost every $x\in E$. Since $\mathbb {1}_E\mu '\ll \mu$, Proposition 3.5 (i) implies that $V(\mu ,x)=V(\mu ',x)$ for $\mu$-almost every $x\in E$. This, together with (18) implies that

$$\begin{aligned} \pi _1(\tau (x))\subseteq V(\mu ,x)\qquad \text {for }\mu \text {-almost every } x\in E. \end{aligned}$$

This concludes the proof. $\square$

## Integrals of Lipschitz Fragments are Pieces of Horizontal Normal Currents

This section is devoted to the proof of Proposition 4.1. Proposition 4.1 shows that any vector-valued measure $\varvec{\mu }$ which can be represented by integration of natural vector-valued measures associated to Lipschitz fragments can be closed to a horizontal normal current by adding to $\varvec{\mu }$ another integral of Lipschitz fragments $\varvec{\sigma }$ whose total variation can be taken singular with respect to any given Radon measure $\eta$. The strategy of the proof partially follows that of [4, Theorem 1.1], but here the necessity to construct a *horizontal* normal current introduces substantial additional difficulties.

### Proposition 4.1

Let (*I*, *dt*) be a $\sigma$-finite measure space, $\eta$ be a positive Radon measure and $t\mapsto \varvec{\mu }_t$ be a family of vector-valued measures satisfying the hypothesis (a) and (b) of Definition 2.11 and such that for almost every $t\in I$ there exists a 1-Lipschitz fragment $\gamma _t:K_t\rightarrow \mathbb {G}$ defined on a compact set $K_t$ of $\mathbb {R}$ such that $\varvec{\mu }_t=\llbracket \gamma _t\rrbracket$. Further more, we let

19 $$\begin{aligned} \varvec{\mu }:=\int _I \varvec{\mu }_t\,dt. \end{aligned}$$

Then, for every $\varepsilon _0>0$ there exists a horizontal normal 1-current $\textbf{T}$ on $\mathbb {G}\cong \mathbb {R}^n$ such that $\partial \textbf{T}=0$, $\mathbb {M}(\textbf{T})\le 2\int _I\mathbb {M}(\varvec{\mu }_t)\,dt+\varepsilon _0$ and $\textbf{T}=\varvec{\mu }+\varvec{\sigma }$, where $\varvec{\sigma }$ and $\eta$ are mutually singular and $\varvec{\sigma }$ is an integration of horizontal Lipschitz fragments as in (19). In particular one can choose $\varvec{\sigma }\perp \Vert \varvec{\mu }\Vert$.

### Remark 4.1

Given a positive Radon measure $\mu$ as in Theorem 7.6, we will construct vector valued measures $\varvec{\mu }$ as in (19) so that $\mu \ll \Vert \varvec{\mu }\Vert$. However, in order to apply the machinery of Section 7, we will need to improve the regularity of $\varvec{\mu }$ to that of a horizontal normal 1-current $\textbf{T}$ without boundary, such that $\mu \ll \Vert \textbf{T}\Vert$. The possibility of this improvement is guaranteed by Proposition 4.1 with the choice $\eta :=\Vert \varvec{\mu }\Vert$.

### Remark 4.2

**Fig. 1:**
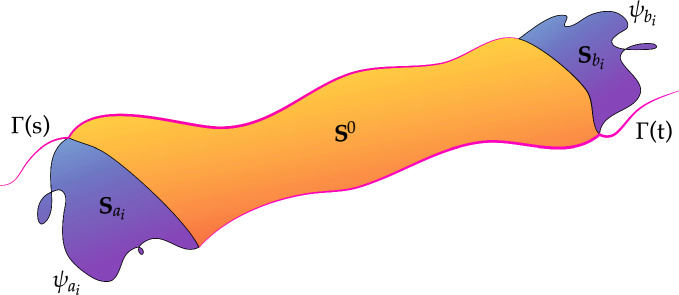
The image shows the filling 2-dimensional surfaces with horizontal boundaries attaching the curves $\Gamma (s)$ to $\Gamma (t)$

(Heuristic for the proof of Proposition 4.1) We describe the strategy of the proof in the simplified case in which the sets $K_t$ on which the fragments $\gamma _t$ are parametrized are finite unions of closed intervals (see Proposition A.4 for the correct reduction). For each interval, one would like to concatenate the fragment $\gamma _t$ with its reverse path. Of course the corresponding measure $\varvec{\sigma }$ could fail to be singular with respect to $\eta$. This however could be fixed by shifting the reverse paths by a miniscule amount, (see Proposition 4.2 for the formal construction) and then reconnecting in a suitable way to the original path to create loops, see Fig. 1.

### Definition 4.1

(Distance on fragments) Denote by $\mathfrak {F}$ the set of all 1-Lipschitz fragments, i.e. the set of all those 1-Lipschitz maps $\gamma :K\rightarrow \mathbb {G}$, where *K* is a compact subset of the real line. Denoted with $d_{eu,\mathscr {H}}$ the Hausdorff distance of the graphs $\textrm{gr}(\gamma ):=\{(t,\gamma (t)):t\in \textrm{dom}(\gamma )\}$, where $\textrm{dom}(\gamma )$ is the domain of $\gamma$. It is immediate to see that $\mathfrak {F}$ is a complete and separable metric space.

Let $N\in \mathbb {N}$. In the following we denote by $\mathcal {X}_N\subseteq \mathfrak {F}$ the family of the fragments $\gamma :\textrm{dom}(\gamma )\rightarrow \mathbb {G}$ where $\gamma$ is a 1-Lipschitz fragment and $\textrm{dom}(\gamma )$ is a union of at most *N* disjoint compact intervals. In addition, we let $\mathcal {X}:=\cup _{N\in \mathbb {N}} \mathcal {X}_N$. Note that $\mathcal {X}_N\subseteq \mathcal {X}_M$ whenever $N\le M$ and that $\mathcal {X}_N$ is closed for every $N\in \mathbb {N}$.

In the next proposition we show how to approximate any element of $\mathfrak {F}$, that is, any 1-Lipschitz fragment $\gamma :K\mapsto \mathbb {G}$, with an element $\tilde{\gamma }$ of $\mathcal {X}_N$ for some *N*, in such a way that the current $\llbracket \gamma \rrbracket -\llbracket \tilde{\gamma }\rrbracket$ has small mass. This is done by first extending $\gamma$ to a 1-Lipschitz curve defined on $I_K:=[\min K,\max K]$ and then restricting the extension to the complement in $I_K$ of those intervals that constitute $I_K\setminus K$ and that have sufficiently large measure.

### Remark 4.3

By Remark 2.2, the Euclidean metric on $\mathbb {G}\cong \mathbb {R}^n$ and any left-invariant homogeneous distance on $\mathbb {G}$ are locally Hölder equivalent therefore the topologies respectively induced by their Hausdorff distances of graphs on $\mathcal {X}_N$ are equivalent as well.

### Proposition 4.2

Let *K* be a compact subset of the real line of positive $\mathscr {L}^1$-measure and assume that $\gamma :K\rightarrow \mathbb {G}$ is a Lipschitz fragment, let $\eta$ be a positive and finite Radon measure on $\mathbb {G}$. Then, there exists a set of full measure of vectors $v\in \mathbb {G}$ such that $\eta$ and  are mutually singular.

### Proof

Without loss of generality we can prove that $\eta$ and  are mutually singular for almost every $v\in B(0,1)$ and we can assume that $\eta$ is finite by restricting $\eta$ to a ball that compactly contains $\cup _{v\in B(0,1)}v*\textrm{im}(\gamma )$. We can further assume that $\eta$ and $\mathscr {L}^n$ are mutually singular. Indeed, if we write $\eta =\eta _a+\eta _s$ where $\eta _a\ll \mathscr {L}^n$ and $\eta _s\perp \mathscr {L}^n$, for every Lipschitz fragment $\gamma$ and for every $v\in B(0,1)$ we have that  and $\eta _a$ are mutually singular. Let $A\subset \mathbb {G}$ be a Borel set such that $\mathscr {L}^n(A)=0$ and $\eta (A^c)=0$ and observe that by Tonelli’s theoremwhere $\tau _v$ is the left translation and the last equality follows from the right-invariance of $\mathscr {L}^n$. The Borelianity of the map  can be checked with the standard techniques and it is omitted. We deduce that  for $\mathscr {L}^n$-almost every $v\in B(0,1)$, so that for those *v*’s the measures $\eta$ and  are mutually singular. $\square$

### Proof of Proposition 4.1

We divide the proof in several steps. As this is one of the most technical proof of the paper, let us anticipate here the content of each step, before entering into the technical details, see also Remark 4.2. In Step 1, we approximate in mass the vector measure $\varvec{\mu }$ with an integral of fragments in $\mathcal {X}_N$. In Step 2 we further approximate in flat norm such integral with a finite sum of fragments in $\mathcal {X}_N$. In Step 3 we perform the “shifting” described in Remark 4.2. In Step 4 we iterate such construction and conclude the proof.

Throughout the proof we fix $0<\varepsilon <\int _I\varvec{\mathbb {M}}(\mu _t)dt/10$. Without loss of generality, we can assume that the $\varvec{\mu }_t$’s are supported on the closed ball *B*(0, *R*) for some $R>0$ and that *I* is $\mathbb {R}$ and *dt* is the Lebesgue measure, see [3, Remark 2.7 (iii)]. Thanks to the assumption that the masses of the $\varvec{\mu }_t$ are summable, i.e. $\int _\mathbb {R}\mathbb {M}(\varvec{\mu }_t)dt<\infty$, for every $\varepsilon >0$ there exists a compact interval $\tilde{I}$ such that $\int _{\mathbb {R}\setminus \tilde{I}}\mathbb {M}(\varvec{\mu }_t)dt<\varepsilon /12$.

**Step 1** (Approximation of $\varvec{\mu }$ in mass with a continuous integral of fragments in $\mathcal {X}_N$) Since the family of measures $\{\varvec{\mu }_t\}_{t\in \tilde{I}}$ satisfies the hypothesis (a) and (b) of Definition 2.11, Proposition A.4 implies that for every $\varepsilon >0$ there exists a Borel set $I_\varepsilon \subset {\tilde{I}}$, $N\in \mathbb {N}$ and a Borel map $\mathfrak {c}_\varepsilon :I_\varepsilon \rightarrow \mathscr {M}(\mathbb {R}^n,\mathbb {R}^n)$ such that

- for every $t\in I_\varepsilon$ we have $\mathfrak {c}_\varepsilon (t)=\llbracket \Gamma (t)\rrbracket$ where the map $\Gamma :I_\varepsilon \rightarrow \mathcal {X}_N$ is a Borel map with respect to the metric $d_{eu,\mathscr {H}}$ introduced in Definition 4.1;
- $$\int _{\tilde{I}\setminus I_\varepsilon }\mathbb {M}(\varvec{\mu }_s)\,ds\le \varepsilon /12$$, $$\int _{I_\varepsilon } \mathbb {M}(\varvec{\mu }_s-\mathfrak {c}_\varepsilon (s))\,ds<\varepsilon /12$$ and $$\Vert \mathbb {M}(\mathfrak {c}_\varepsilon (s))\Vert _{L^\infty (I_\varepsilon )} <\infty $$.

By property (b) of Definition 2.11 and the assumption that each $\varvec{\mu }_t$ is supported on *B*(0, *R*), we may assume, that $\mathfrak {c}_\varepsilon (s)$ are supported in the ball *B*(0, 2*R*). Furthermore, since the measures $\mathfrak {c}_\varepsilon (s)$ have uniformly bounded masses, we deduce that $\mathfrak {c}_\varepsilon$ takes values in a complete separable metric space. Thanks to Lusin’s theorem and by the absolute continuity of the integral, guaranteed by property (b) of Definition 2.11, we can find a closed subset $J\subset I_\varepsilon$ and a (possibly new and larger) $N\in \mathbb {N}$ such that $\int _{I_\varepsilon \setminus J} \mathbb {M}({\varvec{\mu }}_s)\,ds\le \varepsilon /12$, $\mathscr {L}^1( I_\varepsilon \setminus J)\le \varepsilon \mathscr {L}^1(I_\varepsilon )$ and

(i) the maps $\mathfrak {c}_\varepsilon :J\rightarrow \mathscr {M}(\mathbb {R}^n,\mathbb {R}^n)$ and $\Gamma :J\rightarrow \mathcal {X}_N$ are continuous with respect to the weak* topology and $d_{eu,\mathscr {H}}$ respectively, see Definition 4.1 and Remark 4.3;
(ii) analogously the function $t\mapsto \mathbb {M}(\Gamma (t))$ can be supposed to be continuous on *J*;
(iii) $$\Gamma (J)\subseteq \{\gamma \in \mathcal {X}_N:\textrm{dom}(\gamma )\subseteq [-N,N]\}$$.
(iv) denoting $\bar{\varvec{\mu }}_1:=\int _J \mathfrak {c}_\varepsilon (t)\,dt$, we see that the vector-valued measure $\varvec{\mu }-\bar{\varvec{\mu }}_1$ is an integral of (horizontal) Lipschitz fragments in the sense of (19) and that if holds that
  20 $$\begin{aligned} \int _{I\setminus J}\mathbb {M}(\varvec{\mu }_s)\,ds\le \varepsilon /6\qquad \text {and}\qquad \int _{J} \mathbb {M}(\varvec{\mu }_s-\mathfrak {c}_\varepsilon (s))\,ds\le \varepsilon /6. \end{aligned}$$

**Step 2** (Flat approximation of $\varvec{{\bar{\mu }}}_1$ with a finite sum $\textbf{N}$ of fragments in $\mathcal {X}_N$) In this step we will prove the following claim. There exists $\delta =\delta (\varepsilon )$ such that for every $\tau _0\in J$ and every Borel set $A\subset J\cap (\tau _0-\delta ,\tau _0+\delta )$ there exists $t_0\in A$ such that denoting $\textbf{N}_A:=\mathscr {L}^1(A)\llbracket \Gamma (t_0)\rrbracket$ and $\textbf{T}_A:=\int _A\llbracket \Gamma (s)\rrbracket ds$ the following holds:

21 $$\begin{aligned} \mathbb {M}(\textbf{N}_A)\le \int _A \mathbb {M}(\llbracket \Gamma (t)\rrbracket )\,dt. \end{aligned}$$

Moreover, there are a horizontal 1-current $\textbf{R}_A$ and an Euclidean 2-current $\textbf{S}_A$ such that

22 $$\begin{aligned} \textbf{N}_A-\textbf{T}_A=\textbf{R}_A+\partial \textbf{S}_A \qquad \text{ with }\qquad \mathbb {M}(\textbf{S}_A)<{ \varepsilon \frac{\mathscr {L}^1(A)}{\mathscr {L}^1(J)}}, \end{aligned}$$

and $\textbf{R}_A=\int _I \llbracket \varvec{\nu }_t\rrbracket \,dt$ , where $t\mapsto \varvec{\nu }_t$ satisfies the hypothesis of Proposition 4.1 and

23 $$\begin{aligned} \int _I\mathbb {M}(\varvec{\nu }_t)\,dt\le { \varepsilon \frac{\mathscr {L}^1(A)}{\mathscr {L}^1(J)}}. \end{aligned}$$

In the next paragraph, we reduce the construction of $t_0$, $\textbf{R}_A$ and $\textbf{S}_A$ satisfying (22) and (23) to the following claim. For every $0<d<1$ and for every $s,t\in J$ such that $d_{eu,\mathscr {H}}(\Gamma (s),\Gamma (t))\le d$ there are a horizontal 1-current $\textbf{R}(t,s)$ and an Euclidean 2-current $\textbf{S}(t,s)$ for which

24 $$\begin{aligned} \llbracket \Gamma (t)\rrbracket -\llbracket \Gamma (s)\rrbracket =\textbf{R}(t,s)+\partial \textbf{S}(t,s),\qquad \text{ with }\qquad \mathbb {M}(\textbf{S}(t,s)) \le C(N)d^{\frac{1}{\mathfrak {s}}}, \end{aligned}$$

where *C*(*N*) is a constant depending only on *N* and $\textbf{R}(t,s):=\sum _{i=1}^{L(t,s)}\varvec{\nu }^i_{t,s}$ with $L(t,s)\le C(N)$ and $\varvec{\nu }^i_{t,s}$ is (the current associated to) a Lipschitz fragment defined on a compact interval with values in $\mathbb {G}$, and

25 $$\begin{aligned} \sum _{i=1}^{L(t,s)}\mathbb {M}(\varvec{\nu }^i_{t,s})\le C(N)d^{\frac{1}{\mathfrak {s}}}. \end{aligned}$$

Further, for every $t\in J$ fixed, the currents $\textbf{R}(t,s)$ and $\textbf{S}(t,s)$ can be chosen in a Borel way on a sufficiently small neighbourhood of *t* in *J*.

Taking for granted that the above claim holds true, we pick $t_0\in J$ in such a way thatWe thus define that

$$\begin{aligned} \textbf{R}_A:=\int _A\textbf{R}(t_0,s)\,ds\qquad \text {and}\qquad \textbf{S}_A:=\int _A\textbf{S}(t_0,s)\,ds. \end{aligned}$$

With these definitions, let us note that

$$\begin{aligned} \textbf{N}_A-\textbf{T}_A=\mathscr {L}^1(A)\llbracket \Gamma (t_0)\rrbracket -\int _A\llbracket \Gamma (s)\rrbracket \,ds\\ =\int _A\textbf{R}(t_0,s)+\partial \textbf{S}(t_0,s)\,ds=\textbf{R}_A+\partial \textbf{S}_A, \end{aligned}$$

where the last identity follows from the fact that the boundary commutes with integration. Note that thanks to the choice of $t_0$ we immediately infer that $\mathbb {M}(\textbf{N}_A)\le \int _A \mathbb {M}(\llbracket \Gamma (s)\rrbracket )ds$ and hence (21) follows. Let us choose $\delta >0$ sufficiently small in such a way that $d_{eu,\mathscr {H}}(\Gamma (s),\Gamma (t_0))\le (\mathscr {L}^1(J)^{-1}C(N)^{-1}\varepsilon )^\mathfrak {s}$ for all $|s-t_0|<\delta$. Note that $\delta$ is independent on $t_0$ by the uniform continuity of $s\mapsto \Gamma (s)$ on *J*. Then

$$\begin{aligned} \mathbb {M}(\textbf{S}_A)= \mathbb {M}\Big (\int _A\textbf{S}(t_0,s)ds\Big )\le C(N)d^{\frac{1}{\mathfrak {s}}}\mathscr {L}^1(A)\le \varepsilon \frac{\mathscr {L}^1(A)}{\mathscr {L}^1(J)}. \end{aligned}$$

This shows that the claim implies (22). Finally, let us note that $\textbf{R}_A=\int _A\sum _{i=1}^{L(s)}\varvec{\nu }_s^i\,ds$ and thus $\textbf{R}_A$ is a horizontal 1-current and

$$\begin{aligned} \int _A\sum _{i=1}^{L(t)}\mathbb {M}(\varvec{\nu }_t^i)\le C(N)d^{\frac{1}{\mathfrak {s}}}\mathscr {L}^1(A)\le \varepsilon \frac{\mathscr {L}^1(A)}{\mathscr {L}^1(J)}. \end{aligned}$$

This concludes the proof of the fact that our claim implies the sought conclusion of step 2.

Let us move to the proof of (24) and (25). Let $s,t\in J$ such that $d_{eu,\mathscr {H}}(\Gamma (s),\Gamma (t))\le d$, and observe that, for every $z\in K_{s,t}:=\textrm{dom}(\Gamma (s))\cap \textrm{dom}(\Gamma (t))$, if holds that

26 $$\begin{aligned} |\Gamma (s)(z)-\Gamma (t)(z)|\le 2d. \end{aligned}$$

Note that if $K_{s,t}=\varnothing$, then (25) follows easily from $d_{eu,\mathscr {H}}(\Gamma (s),\Gamma (t))\le d$. Indeed, since $K_{s,t}=\varnothing$, on the one hand we know that $\textrm{dom}(\Gamma (t))$ and $\textrm{dom}(\Gamma (s))$ are disjoint. On the other, that they are the union of at most *N* intervals and

$$\begin{aligned} d_{eu,H}(\textrm{dom}(\Gamma (t)),\textrm{dom}(\Gamma (s)))\le d_{eu,H}(\Gamma (s),\Gamma (t))\le d. \end{aligned}$$

This implies in particular that

$$\begin{aligned} \sup _{x\in \textrm{dom}(\Gamma (s))}\textrm{dist}(x,\textrm{dom}(\Gamma (t)))\le d \text {and}\sup _{y\in \textrm{dom}(\Gamma (s))}\textrm{dist}(y,\textrm{dom}(\Gamma (t)))\le d. \end{aligned}$$

The fact that the two sets are disjoint with the above estimates implies that $\mathscr {L}^1(\textrm{dom}(\Gamma (t))\cup \textrm{dom}(\Gamma (s)))\le 2Nd$. Therefore, they satisfy $\llbracket \Gamma (t)\rrbracket -\llbracket \Gamma (s)\rrbracket =\textbf{R}(t,s)$, with

$$\begin{aligned} \mathbb {M}(\textbf{R}(t,s))\le 2Nd, \end{aligned}$$

thanks to the 1-Lipschitzianity of the curves $\Gamma (s)$ and $\Gamma (t)$, which is the sought estimate (25).

Suppose now $K_{s,t}\ne \varnothing$. Consider now the map $g:K_{s,t}\times [0,d]\rightarrow \mathbb {G}$ given by

$$\begin{aligned} g(\sigma ,\tau )=\left( 1-\frac{\tau }{d}\right) \Gamma (s)(\sigma )+\frac{\tau }{d}\Gamma (t)(\sigma ). \end{aligned}$$

Let us observe that thanks to our choice of *J* and since $s,t\in J$ we have that $K_{s,t}$ has the form $K_{s,t}=\bigcup _{i=1}^{M}[a_i,b_i]$, for some number $M\le 2N$. Moreover it is easy to check that *g* is 3-Lipschitz, because it is 1-Lipschitz in the variable $\sigma$, being an Euclidean convex combination of 1-Lipschitz maps, and it is 2-Lipschitz in the variable $\tau$, due to (26). Let us further denote that27where $g_\#$ denotes the pushforward of currents through the map *g*, see [34, §7.4.2]. Note that since $\llbracket K_{s,t}\times [0,d]\rrbracket$ is a 2-dimensional normal current in $\mathbb {R}^2$, the 2-current $\textbf{S}^0$ is well defined thanks to [22, §4.1.14] or [34, Lemma 7.4.3]. Notice also that by definition of $\textbf{S}^0$ and $\textbf{R}^0$ we have

28 $$\begin{aligned} \llbracket \Gamma (t)\rrbracket -\llbracket \Gamma (s)\rrbracket =\textbf{R}^0+\partial \textbf{S}^0, \end{aligned}$$

and this can be seen by recalling that pushforward and boundary are two commuting operators, i.e. $\partial g_\#\textbf{T}=g_\#\partial \textbf{T}$ for every 2-dimensional normal current $\textbf{T}$. However $\textbf{S}^0$ and $\textbf{R}^0$ do not satisfy (24) and (25) since $\textbf{R}^0$ is not horizontal.

Therefore, for every $i=1,\ldots , M$ let us denote $\psi _{a_i}$ a geodesic joining $g(a_i,0)$ to $g(a_i,d)$ and $\psi _{b_i}$ a geodesic joining $g(b_i,0)$ to $g(b_i,d)$ and define

$$\begin{aligned} { \textbf{R}^i:=\llbracket \psi _{a_i}\rrbracket -g(a_i,\cdot )_\sharp \llbracket [0,d]\rrbracket -\llbracket \psi _{b_i}\rrbracket +g(b_i,\cdot )_\sharp \llbracket [0,d]\rrbracket ,} \end{aligned}$$

for $i=1,\ldots , M$. Since we are working inside the fixed compact set *B*(0, *R*), there exists a constant $C(R,\mathbb {G})$ such that

29 $$\begin{aligned} \mathbb {M}(\textbf{R}^i)\le C(R,\mathbb {G}) d^{\frac{1}{\mathfrak {s}}}, \end{aligned}$$

and this is a consequence of Remark 2.2. Let $\textbf{S}_{a_i}$ and $\textbf{S}_{b_i}$ be 2-dimensional currents with boundary $g(a_i,\cdot )_\sharp \llbracket [0,d]\rrbracket -\llbracket \psi _{a_i}\rrbracket$ and $g(b_i,\cdot )_\sharp \llbracket [0,d]\rrbracket -\llbracket \psi _{b_i}\rrbracket$, respectively, and

30 $$\begin{aligned} \mathbb {M}(\textbf{S}_{a_i})+\mathbb {M}(\textbf{S}_{b_i})\le Cd^{\frac{1}{\mathfrak {s}}}. \end{aligned}$$

The choice of such $\textbf{S}_{a_i}$’s and $\textbf{S}_{b_i}$’s, with the above control on their mass, is achievable thanks to the classical cone construction, see for instance [48, (26.26)]. We further define $\textbf{S}^i:=\textbf{S}_{a_i}-\textbf{S}_{b_i}$ and

31 $$\begin{aligned} \textbf{S}(t,s):=\textbf{S}^0+\sum _{i=1}^{M}\textbf{S}^i\qquad \text {and}\qquad \textbf{R}(t,s):=\textbf{R}^0+\sum _{i=1}^{M}\textbf{R}^i. \end{aligned}$$

Let us note that $\textbf{S}(t,s)$ is an Euclidean 2-current, and sincewe infer that $\textbf{R}(t,s)$ is a horizontal 1-current andwhere the last inequality follows from (29). Hence, the above bound shows that (25) holds. In addition, (28) together with the definition of $\varvec{S}_{a_i},\varvec{S}_{b_i}$, $R_0$ and $R_i$ we have $\llbracket \Gamma (t)\rrbracket -\llbracket \Gamma (s)\rrbracket =\textbf{R}(t,s)+\partial \textbf{S}(t,s)$. Thanks to [22, §4.1.14] we have that

$$\begin{aligned} \mathbb {M}(\textbf{S}^0)\le \textrm{Lip}(g)^2\mathbb {M}(\llbracket K_{s,t}\times [0,d]\rrbracket )\le 18Nd, \end{aligned}$$

and thus by (30), we infer that $\mathbb {M}(\textbf{S}(t,s))\le C(N)d^\frac{1}{\mathfrak {s}}$. This concludes the proof of (24) and hence of the Step 2.

**Step 3** (Translating the curves of $\textbf{N}$ infinitesimally to make them singular with respect to $\eta$) Let us take $\delta$ as in the beginning of Step 2 and finitely many disjoint Borel sets $A_j$ of diameter less than $2\delta$ such that $\bigcup _j A_j=J$. Denote $\textbf{N}_{A_j}$ and $t_{0,j}\in A_j$ be the corresponding currents and times constructed in Step 2 and $\textbf{T}_1:=\sum _j \textbf{N}_{A_j}=\sum _j\mathscr {L}^1(A_j)\llbracket \Gamma (t_{0,j})\rrbracket$. Notice that by (20) and (21) there are a horizontal 1-current $\textbf{R}_1^\alpha$ and an Euclidean 2-current $\textbf{S}_1^\alpha$ such that

32 $$\begin{aligned} \mathbb {M}(\textbf{T}_1)\le \sum _j\mathscr {L}^1(A_j)\mathbb {M}(\llbracket \Gamma (t_{0,j})\rrbracket )\le \int _J\Vert \varvec{\mu }_t\Vert \, dt \end{aligned}$$

and by (22)

33 $$\begin{aligned} \bar{\varvec{\mu }}_1-\textbf{T}_1={\textbf{R}}_1^\alpha +\partial {\textbf{S}}_1^\alpha , \qquad \text{ with }\qquad \mathbb {M}({\textbf{S}}_1^\alpha )<\varepsilon , \end{aligned}$$

where $\bar{\varvec{\mu }}_1$ is the vector-valued measure defined in (iv) in Step 1, $\textbf{S}_1^\alpha$ is a 2-dimensional (Euclidean) normal current, $\textbf{R}_1^\alpha =\int _I\varvec{\nu }_t\,dt$ and here $\varvec{\nu }_t$ are horizontal 1-currents associated to Lipschitz fragments with values in $\mathbb {G}$ such that

$$\begin{aligned} \int _I\mathbb {M}(\varvec{\nu }_t)\,dt\le \varepsilon . \end{aligned}$$

Let $\gamma :[0,1]\rightarrow \mathbb {G}$ be a Lipschitz curve. Note that applying Proposition 4.2 to the curve $\gamma$ we infer that for $\mathscr {L}^n$-almost every $v\in B(0,\varepsilon ^{2\mathfrak {s}})$ we have that $\llbracket v*\gamma \rrbracket$ and $\eta$ are mutually singular since $\llbracket v*\gamma \rrbracket$ and  are mutually absolutely continuous and that $d_{eu,\mathscr {H}}(\gamma ,v*\gamma )\le \varepsilon ^{2\mathfrak {s}}$. In addition, we can choose such $\delta _\gamma <\varepsilon ^{2\mathfrak {s}}$ so small that the argument in Step 2 with the choice $d=\delta _\gamma$ implies that we can find a horizontal 1-current $\textbf{R}_\gamma$ and an Euclidean 2-current $\textbf{S}_\gamma$ such that

34 $$\begin{aligned} \llbracket \gamma \rrbracket -\llbracket v*\gamma \rrbracket =\textbf{R}_\gamma +\partial \textbf{S}_\gamma ,\qquad \text{ with }\qquad \mathbb {M}(\textbf{S}_\gamma ) \le \varepsilon /\mathscr {L}^1(J), \end{aligned}$$

where $\textbf{R}_\gamma :=\sum _{i=1}^L\varvec{\nu }^i_{t,s}$ and each $\varvec{\nu }^i_{t,s}$ is a finite sum of Lipschitz curves defined on compact intervals with values in $\mathbb {G}$, and $\sum _{i=1}^L\mathbb {M}(\varvec{\nu }^i_{t,s})\le \varepsilon /6\mathscr {L}^1(J)$. Since ${\varvec{T}}_1=\sum _{j}\mathscr {L}^1(A_j)\llbracket \Gamma (t_{0,j})\rrbracket$, applying the above argument for each *j* we infer that there are vectors $v_j\in B(0,\varepsilon ^{2\mathfrak {s}})$ as above. Therefore, defined $\textbf{Z}_1:=\sum _{j}\mathscr {L}^1(A_j)\llbracket v_j*\gamma _j\rrbracket$ we infer that

$$\begin{aligned} \begin{aligned} \varvec{\mu }-{\textbf {Z}}_1&=(\varvec{\mu }-\bar{\varvec{\mu }}_1)+(\bar{\varvec{\mu }}_1-\textbf{Z}_1)=(\varvec{\mu }-\bar{\varvec{\mu }}_1)+\bar{\varvec{\mu }}_1-{\textbf {T}}_1+{\textbf {T}}_1-\textbf{Z}_1\\&=(\varvec{\mu }-\bar{\varvec{\mu }}_1)+{\textbf{R}}_1^\alpha +\partial {\textbf{S}}_1^\alpha +\sum _{j}\mathscr {L}^1(A)(\llbracket \gamma _j\rrbracket -\llbracket v*\gamma _j\rrbracket )\\&=(\varvec{\mu }-\bar{\varvec{\mu }}_1)+{\textbf{R}}_1^\alpha +\partial {\textbf{S}}_1^\alpha +\sum _j\mathscr {L}^1(A)(\textbf{R}_{\gamma _j}+\partial \textbf{S}_{\gamma _j})\\&=(\varvec{\mu }-\bar{\varvec{\mu }}_1)+\Big ({\textbf{R}}_1^\alpha +\sum _j\mathscr {L}^1(A)\textbf{R}_{\gamma _j}\Big )+\partial \Big ( {\textbf{S}}_1^\alpha +\sum _j\mathscr {L}^1(A)\partial \textbf{S}_{\gamma _j}\Big )\\&=:(\varvec{\mu }-\bar{\varvec{\mu }}_1)+{\textbf{R}}_1+\partial {\textbf{S}}_1. \end{aligned} \end{aligned}$$

In addition, note that

$$\begin{aligned} \mathbb {M}(\mathbf {Z_1})\le \sum _{j}\mathscr {L}^1(A_j)\mathbb {M}(\llbracket v_j*\Gamma (t_{0,j})\rrbracket )\\ \le \sum _{j}\mathscr {L}^1(A_j)\mathbb {M}(\llbracket \Gamma (t_{0,j})\rrbracket )\le \int _I\mathbb {M}(\varvec{\mu }_t)\,dt. \end{aligned}$$

Define for future convenience ${\varvec{\mu }}_2:=(\varvec{\mu }-\bar{\varvec{\mu }}_1)+{\textbf{R}}_1$ and note that ${\textbf{R}}_1$ can be written as $\int _I\llbracket \sigma _t\rrbracket \,dt$ where $t\mapsto \sigma _t$ is a suitable Borel map with values in $\mathfrak {F}$ defined on compact intervals and such that $\int _I\mathbb {M}(\llbracket \sigma _t\rrbracket )\,dt\le \varepsilon /3$. Note that $t\mapsto \llbracket \sigma _t\rrbracket$ coincides with a Borel thanks to Lemma A.3 up to negligible sets. With abuse of notations we will denote by $t\mapsto \llbracket \sigma _t\rrbracket$ such Borel map. This implies that

$$\begin{aligned} \varvec{\mu }_2=\varvec{\mu }-\bar{\varvec{\mu }}_1+\textbf{R}_1=\int _{I\setminus J}\varvec{\mu }_t\, dt+\int _{J}(\varvec{\mu }_t-\mathfrak {c}_\varepsilon (t))\,dt+\int _I\llbracket \sigma _t\rrbracket \,dt=\int _I \varvec{\mu }_{2,t}\,dt, \end{aligned}$$

where in the last identity we reparametrized the integrals in the second term thanks to [3, Remark 2.7 (iii)]. Furthermore, we note that

$$\begin{aligned} \int _I \mathbb {M}(\varvec{\mu }_{2,t})\,dt\le \varepsilon . \end{aligned}$$

Finally it is immediately appart that $\mathbb {M}(\textbf{S}_1)\le 2\varepsilon .$

**Step 4** (Iteration of the previous steps) We obtain the current $\textbf{T}$ by iterating on the previous steps, as follows. Chosen $\varepsilon =\varepsilon _0/4$ and applying Steps 1, 2 and 3 to the fragments $\{\varvec{\mu }_t\}_{t\in I}$ we obtain currents $\textbf{ Z}_1$, $\textbf{R}_1$, $\textbf{S}_1$ and a measurable family $\{\varvec{\mu }_{2,t}\}_{t\in I}$ of currents associated Lipschitz fragments with values in $\mathbb {G}$ with the properties described in Step 3 above. In particular, $\textbf{ Z}_1$ and $\eta$ are mutually singular.

We can thus apply again Steps 1, 2 and 3 with the choice $\varepsilon =\varepsilon _0/4^2$ to the fragments $\{\varvec{\mu }_{2,t}\}_{t\in I}$ obtaining currents $\textbf{ Z}_2$, $\textbf{R}_2$, $\textbf{S}_2$ and a vector-valued measure $\bar{\varvec{\mu }}_2$ such that

$$\begin{aligned} \varvec{\mu }_2-\textbf{Z}_2=(\varvec{\mu }_2-\bar{\varvec{\mu }}_2) +{\textbf{R}}_2+\partial {\textbf{S}}_2,\quad \mathbb {M}({\textbf{S}}_2)\le 2\varepsilon _0/4^2,\quad \mathbb {M}(\textbf{Z}_2)\le \int _I\mathbb {M}(\varvec{\mu }_{2,t})\,dt, \end{aligned}$$

where $\varvec{\mu }_2=\int _I\varvec{\mu }_{2,t}dt$ and $\varvec{\mu }_3:=(\varvec{\mu }_2-\bar{\varvec{\mu }}_2) +{\textbf{R}}_2$ can be represented by $\varvec{\mu }_3=\int _I\varvec{\mu }_{3,t}\,dt$ where $\{\varvec{\mu }_{3,t}\}_{t\in I}$ is a measurable family of currents associated Lipschitz fragments with values in $\mathbb {G}$ such that

$$\begin{aligned} \mathbb {M}(\varvec{\mu }_3)\le \int _I\mathbb {M}(\varvec{\mu }_{3,t})\,dt\le \varepsilon _0/4^2. \end{aligned}$$

Note further that

$$\begin{aligned} \varvec{\mu }=(\textbf{Z}_1+\textbf{Z}_2)+\varvec{\mu }_3+\partial (\textbf{S}_1+\textbf{S}_2). \end{aligned}$$

Iterating the procedure, we construct a sequence of horizontal 1-currents $\{\textbf{Z}_i\}_{i\in \mathbb {N}}$, a sequence of Euclidean 2-currents $\{\textbf{S}_i\}_{i\in \mathbb {N}}$ and a sequence of family of currents $\{\varvec{\mu }_{j,t}:t\in I\}$ such that, defined $\varvec{\mu }_j:=\int _I\varvec{\mu }_{j,t}\,dt$ for every $j\in \mathbb {N}$, we have

35 $$\begin{aligned} \varvec{\mu }-\sum _{i=1}^j\textbf{Z}_i=\varvec{\mu }_{j+1}+\partial \Big (\sum _{i=1}^j\textbf{S}_i\Big ), \end{aligned}$$

we have $\mathbb {M}(\textbf{S}_i)\le 2\varepsilon _0/4^i$ for every $i\in \mathbb {N}$ and

$$\begin{aligned} \mathbb {M}(\textbf{Z}_i)\le \int _I \mathbb {M}(\varvec{\mu }_{i,t})\,dt\le \varepsilon _0/4^i\qquad \text {and}\qquad \mathbb {M}(\varvec{\mu }_i)\le \int _I\mathbb {M}(\varvec{\mu }_{i,t})\,dt\le \varepsilon _0/4^i, \end{aligned}$$

for every natural $i\ge 2$. Note that defined $\varvec{\sigma }:=-\sum _{i\in \mathbb {N}}\textbf{Z}_i$ and $\textbf{S}:=\sum _{i\in \mathbb {N}}\textbf{S}_i$ these conditions imply that

$$\begin{aligned} \varvec{\mu }+\varvec{\sigma }=\partial \textbf{S},\qquad \mathbb {M}(\varvec{\sigma })\le \int _I\mathbb {M}(\varvec{\mu }_t)dt+\varepsilon _0,\qquad \mathbb {M}(\textbf{S})\le \varepsilon _0, \end{aligned}$$

where the first identity holds since $\mathbb {M}(\varvec{\mu }_j)$ converges to 0 as $j\rightarrow \infty$. Finally, denoted $\textbf{T}:=\varvec{\mu }+\varvec{\sigma }$ we get the sought conclusion since by construction each $\textbf{Z}_i$ is mutually singular with respect to $\eta$ and since $\partial \textbf{T}=0$ as $\textbf{T}$ is already the boundary of $\textbf{S}$. $\square$

### Corollary 4.3

Let (*I*, *dt*) be a $\sigma$-finite measure space, $\eta$ be a positive Radon measure and $t\mapsto \varvec{\mu }_t$ be a family of vector-valued measures satisfying the hypothesis (a) and (b) of Definition 2.11 and such that for almost every $t\in I$ there exists a 1-Lipschitz fragment $\gamma _t:K_t\rightarrow \mathbb {G}$ defined on a compact set $K_t$ of $\mathbb {R}$ such that $\varvec{\mu }_t=\llbracket \gamma _t\rrbracket$. Suppose, further, that there exists $\vartheta \in (0,1)$ and $e\in V_1$ such that

$$\begin{aligned} D\gamma _t(s)\in C(e,\vartheta )\qquad \text {for }\mathscr {L}^1\text {-almost every } s\in K_t\text { and almost every }t\in I. \end{aligned}$$

Then, defined $\varvec{\mu }:=\int _I\varvec{\mu }_t\,dt$ we have $\Vert \varvec{\mu }\Vert$ and $\int _I\Vert \varvec{\mu }_t\Vert \,dt$ are mutually absolutely continuous and for every $\varepsilon _0>0$ there exists a horizontal normal 1-current $\textbf{T}$ on $\mathbb {G}\cong \mathbb {R}^n$ such that $\partial \textbf{T}=0$, $\mathbb {M}(\textbf{T})\le 2\int _I\mathbb {M}(\varvec{\mu }_t)\,dt+\varepsilon _0$ and $\textbf{T}=\varvec{\mu }+\varvec{\sigma }$, where $\varvec{\sigma }$ and $\eta$ are mutually singular and $\varvec{\sigma }$ is an integration of horizontal Lipschitz fragments as in (19) and

36 $$\begin{aligned} \frac{d\textbf{T}(x)}{d\Vert \textbf{T}\Vert }\in \mathscr {C}(x)[C(e,\vartheta )]\setminus \{0\}\qquad \text {for }\Vert \varvec{\mu }\Vert \text {-almost every }x\in \mathbb {G}. \end{aligned}$$

### Proof

First of all, it is immediate to see that $\Vert \varvec{\mu }\Vert \ll \nu$ where $\nu :=\int _I\varvec{\Vert }\varvec{\mu }_t\Vert \,dt$. Let us then prove that $\nu \ll \Vert \varvec{\mu }\Vert$. Note that for every $t\in I$, for every Borel set we have that $\Vert \varvec{\mu }_t\Vert$ is the measure that acts as

$$\begin{aligned} \Vert \varvec{\mu }_t\Vert (E)=\int _{\gamma _t^{-1}(E)} |\gamma _t'(s)|\,ds\qquad \text {for every Borel set }E\subseteq \mathbb {G}. \end{aligned}$$

Therefore let *E* be any bounded Borel set. By the very definition of total variation we know that

37 $$\begin{aligned} \Vert \varvec{\mu }\Vert (E)\ge \Big |\int _I\int _{\gamma _t^{-1}(E)}\gamma _t'(s)\,ds\,dt\Big |\ge \Big \langle \int _I\int _{\gamma _t^{-1}(E)}\gamma _t'(s)\,ds\,dt\,;e\Big \rangle . \end{aligned}$$

It is furthermore clear that38However, let us note that for every $s\in \gamma _t^{-1}(E)$ such that $\gamma _t'(s)\ne 0$, we have

39 $$\begin{aligned} \Big \langle \frac{\gamma _t'(s)}{|\gamma _t'(s)|};\,e\Big \rangle =\frac{\langle D\gamma (t);\,e\rangle }{|\gamma _t'(s)|}\ge (1-\vartheta ^2)\frac{|D\gamma _t(t)|}{|\gamma _t'(s)|}\ge \frac{(1-\vartheta ^2)}{\Vert \mathscr {C}\Vert _{\infty ,E}}. \end{aligned}$$

Therefore, from (37), (38) and (39) we infer thatThis shows that if $\Vert \varvec{\mu }\Vert (E)=0$, we deduce that $\int _I\Vert \varvec{\mu }_t\Vert (E)\,dt=0$. This shows that ${\nu }\ll \Vert \varvec{\mu }\Vert$.

Thanks to Proposition 4.1 we know that for every $\varepsilon _0>0$ there exists a horizontal normal 1-current $\textbf{T}$ on $\mathbb {G}\cong \mathbb {R}^n$ such that $\partial \textbf{T}=0$, $\mathbb {M}(\textbf{T})\le 2\int _I\mathbb {M}(\varvec{\mu }_t)\,dt+\varepsilon _0$ and $\textbf{T}=\varvec{\mu }+\varvec{\sigma }$, where $\varvec{\sigma }$ and $\varvec{\mu }$ are mutually singular and $\varvec{\sigma }$ is an integration of horizontal Lipschitz fragments as in (19).

We are left to check (36). Thanks to [6, Theorem 2.22], we know that

$$\begin{aligned} \frac{d\textbf{T}(x)}{d\Vert \varvec{\mu }\Vert }= &   \lim _{r\rightarrow 0}\frac{\textbf{T}(U(x,r))}{\Vert \varvec{\mu }\Vert (U(x,r))}=\lim _{r\rightarrow 0}\frac{\varvec{\mu }(U(x,r))+\varvec{\sigma }(U(x,r))}{\Vert \varvec{\mu }\Vert (U(x,r))}\\= &   \lim _{r\rightarrow 0}\frac{\varvec{\mu }(U(x,r))}{\Vert \varvec{\mu }\Vert (U(x,r))},\qquad \text {for } \Vert \varvec{\mu }\Vert \text {-almost every }x\in \mathbb {G}, \end{aligned}$$

where as usual *U*(*x*, *r*) denotes the closed Euclidean ball with centre *x* and radius $r>0$. Reasoning as above one immediately infers that

40 $$\begin{aligned} \frac{d\textbf{T}(x)}{d\Vert \textbf{T}\Vert }=\lim _{r\rightarrow 0}\frac{\varvec{\mu }(U(x,r))}{\Vert \varvec{\mu }\Vert (U(x,r))},\qquad \text {for } \Vert \varvec{\mu }\Vert \text {-almost every }x\in \mathbb {G}. \end{aligned}$$

And further, since $\textbf{T}$ is horizontal, we deduce by Proposition 4.1 that $\frac{d\textbf{T}(x)}{d\Vert \textbf{T}\Vert }\in H\mathbb {G}(x)$ for $\Vert \textbf{T}\Vert$-almost every $x\in \mathbb {G}$. Finally, for $\Vert \varvec{\mu }\Vert$-almost every $x\in \mathbb {G}$ and every $r>0$ we have

$$\begin{aligned} \begin{aligned} \langle \varvec{\mu }(U(x,r));\,e\rangle&=\Big \langle \int _I\int _{\gamma _t^{-1}(U(x,r))}\gamma _t'(s)\,ds\,dt\,;e\Big \rangle =\int _I\int _{\gamma _t^{-1}(U(x,r))}\langle \gamma _t'(s);\,e\rangle \,ds\,dt\\&\ge (1-\vartheta ^2)\int _I\int _{\gamma _t^{-1}(U(x,r))}|\pi _1(\gamma _t'(s))|\,ds\,dt \\&\ge (1-\vartheta ^2)\Big |\pi _1\Big (\int _I\int _{\gamma _t^{-1}(U(x,r))}\gamma _t'(s)\,ds\,dt \Big )\Big |\\&=(1-\vartheta ^2)|\pi _1\big (\varvec{\mu }(U(x,r))\big )|. \end{aligned} \end{aligned}$$

The above computation and (40) show immediately that $\frac{d\textbf{T}(x)}{d\Vert \textbf{T}\Vert }\in \mathscr {C}(x)[C(e,\vartheta )]\setminus \{0\}$ for $\Vert \varvec{\mu }\Vert$-almost every $x\in \mathbb {G}$. $\square$

## Auxiliary Decomposability Bundle

In this section we relate the decomposability bundle to the existence of suitable horizontal normal currents, which is the key step in the proof of the main theorem; compare with [3, Section 6].

### Definition 5.1

(Auxiliary decomposability bundle) Let $\mu$ be a Radon measure on $\mathbb {G}$. For every $x\in \textrm{supp}(\mu )$ we denote with $N(\mu ,x)$ the set of all vectors of $v\in H\mathbb {G}(x)$, see (5), for which there exists a horizontal 1-dimensional normal current $\textbf{T}$ with $\partial \textbf{T}=0$ such thatwhere we recall that *U*(*x*, *r*) denotes the closed Euclidean ball centred at *x* of radius *r*.

### Remark 5.1

Let us recall that throughout the paper we have identified $\mathbb {G}$ with $\mathbb {R}^n$ by means of the exponential map. Therefore, the elements of $N(\mu ,x)$ are vectors of $\mathbb {R}^n$ and hence $N(\mu ,x)$ it is easily seen to be a linear subspace of $\mathbb {G}\cong \mathbb {R}^n$. More specifically, $N(\mu ,x)$ is a vector subspace of $\mathscr {C}(x)[V_1]=H\mathbb {G}(x)$.

### Lemma 5.1

For every Radon measure $\mu$, the map $x\mapsto N(\mu ,x)$ seen as a map from $\mathbb {G}\cong \mathbb {R}^n$ to $\textrm{Gr}_{eu}(\mathbb {G})$, see Definition 2.4, is universally measurable.

### Proof

The proof can be achieved following the argument used to prove [3, Lemma 6.9]. (the only difference is that here the vector *v* is forced to lie in the smooth distribution of $n_1$-dimensional planes $\mathscr {C}(x)[V_1]=H\mathbb {G}(x)$): $\square$

### Proposition 5.2

For every Radon measure $\mu$ on $\mathbb {G}$ and every 1-dimensional horizontal normal current $\textbf{T}$ with $\partial \textbf{T}=0$, if we denote by $\tau$ the Radon-Nikodym derivative of $\textbf{T}$ with respect to $\mu$, we have

$$\begin{aligned} \tau (x)\in N(\mu ,x)\qquad \text { for }\mu \text {-almost every }x\in \mathbb {G}. \end{aligned}$$

### Proof

Indeed, let $\textbf{T}=\tau \mu +\varvec{\sigma }$, where $\varvec{\sigma }$ and $\mu$ are mutually singular. Then by Lebesgue-Besicovitch differentiation theorem, see [6, Theorem 2.22], we havefor $\mu$-almost every $x\in \mathbb {G}$, which in turn implies that $\tau (x)\in N(\mu ,x)$. $\square$

The next lemma is the counterpart of [3, Lemma 6.11]. The main difference in the proof of these two statements is that in Lemma 5.3 we are adding the requirement that $\textbf{T}$ is horizontal. In the proof we find disjoint Euclidean balls *U* where the measure $\tau \mu$ is well approximated by some boundary-less current $\textbf{T}_U$. In [3, Lemma 6.11] the idea is to close each $\textbf{T}_U$ on the boundary of *U*. This however is not possible to obtain here, still maintaining $\textbf{T}$ horizontal. Therefore, we must employ Proposition 4.1 to suitably patch together all the $\textbf{T}_U$s.

### Lemma 5.3

Let $\mu$ be a Radon measure on $\mathbb {G}$ and suppose $\tau$ is an $L^1(\mu )$ vector field such that $\tau (x)\in N(\mu ,x)$ for $\mu$-almost every $x\in \mathbb {G}$. Then, for every $\varepsilon _0>0$ there exists an horizontal normal current $\textbf{T}$ on $\mathbb {G}$ such that

(i) $\Vert \tilde{\tau }-\tau \Vert _{L^1(\mu )}\le \Vert \tau \Vert _{L^1(\mu )}/2$ where $\tilde{\tau }$ is the Radon-Nikodym derivative of $\textbf{T}$ with respect to $\mu$;
(ii) $$\partial \textbf{T}=0$$ and $$\mathbb {M}(\textbf{T})\le 2(1+2\varepsilon _0) \Vert \tau \Vert _{L^1(\mu )}$$.

### Proof

If $\tau (x)=0$ for $\mu$-almost every $x\in \mathbb {G}$ there is nothing to prove and hence we may assume that $\tau$ is non-trivial. Let $0<\varepsilon \le \Vert \tau \Vert _{L^1(\mu )}\varepsilon _0/(4\mathbb {M}(\mu )+2)$ and note that thanks to Lebesgue’s differentiation theorem, see for instance [22, Corollary 2.9.9], for $\mu$-almost every $x\in \mathbb {G}$ there exists an $r_0(x)>0$ such that for every $0<s<r_0(x)$ we haveTherefore, thanks to [22, Corollary 2.8.15] and the fact that $\tau (x)\in N(\mu ,x)$ for $\mu$-almost every $x\in \mathbb {G}$, we can find countably many closed and disjoint Euclidean balls $\{U(x_i,r_i)\}_{i\in \mathbb {N}}$ such that

1. $$\mu (\mathbb {G}\setminus \bigcup _iU(x_i,r_i))=0$$,
2. for every $i\in \mathbb {N}$ we have  and we can find a 1-dimensional horizontal normal current $\textbf{T}_i=\tau _i\mu _i$ such that $\partial \textbf{T}_i=0$ and 41

In the following we will take the currents $\textbf{T}_i$, we will decompose each one in curves thanks to Smirnov’s theorem Theorem 2.10 and we will restrict to each ball $U(x_i,r_i)$ the curves of the decomposition of $\textbf{T}_i$. Then, we will be able to apply Proposition 4.1 to patch together these curves and get the normal current $\textbf{T}$.

Thanks to Theorem 2.10 and [3, Remark 2.7 (iii)], for every $i\in \mathbb {N}$ we can find a family of vector-valued measures $t\mapsto \varvec{\eta }_t^i$ satisfying the hypothesis (a) and (b) of Definition 2.11 with the measure space $(\mathbb {R},\mathscr {L}^1)$ and such that for every $i\in \mathbb {N}$ and for almost every $t\in \mathbb {R}$ there exists a 1-Lipschitz curve $\gamma _t^i:K_t\rightarrow \mathbb {G}$ such that , recall that $\tau _{\gamma _t^i}$ and $\rho _{t,i}$ were introduced in Definition 2.12, and that

$$\begin{aligned} \langle \textbf{T}_i\, ; \, \omega \rangle =&\int _\mathbb {R}\langle \llbracket \gamma _t^i\rrbracket \, ; \, \omega \rangle \,dt\quad \!\text {for every smooth and compactly supported }1\text {-form }\omega , \end{aligned}$$

In addition, Theorem 2.10 tells us also that $\tau _i=\tau _{\gamma ^i_t}$ for -almost every $x\in \mathbb {R}^n$ and almost every $t\in \mathbb {R}$. Let *I* be the measure space defined as $\mathbb {N}\times \mathbb {R}$ endowed with the measure $\mathscr {H}^0\otimes \mathscr {L}^1$. Here the parameter $i\in \mathbb {N}$ indexes the ball $U(x_i,r_i)$ and for a fixed $i\in \mathbb {N}$, the parameter $t\in \mathbb {R}$ indexes the curve $\gamma _t^i$. Let . Note, further, that Therefore, thanks to Proposition 4.1 there exists a 1-dimensional horizontal normal current $\textbf{T}$ such that $\partial \textbf{T}=0$, $\mathbb {M}(\textbf{T})\le (4\mathbb {M}(\mu )+1)\varepsilon +2\Vert \tau \Vert _{L^1(\mu )}$ and $\textbf{T}=\varvec{\nu }+\varvec{\sigma }$ where

$$\begin{aligned} \varvec{\nu }:=\int _{\mathbb {N}\times \mathbb {R}}\varvec{\eta }(i,t)d\mathscr {H}^0(j)\otimes \mathscr {L}^1(t), \end{aligned}$$

and the measures  and $\varvec{\sigma }$ are mutually singular. This guarantees that the Radon-Nikodym derivative of $\textbf{T}$ with respect to $\mu$ inside the ball $U(x_i,r_i)$ coincides with $\tau _i$. More precisely, thanks to the choice of $\varvec{\sigma }$ and to Radon-Nikodym’s decomposition theorem we can write $\textbf{T}$ as42where the $\varvec{\sigma }_i$’s are vector valued measures supported on $U(x_i,r_i)$ singular with respect to , such that . Hence, if we write $\textbf{T}$ as $\textbf{T}=\tilde{\tau }\mu +\varvec{\tilde{\sigma }}$, where $\mu$ and $\varvec{\tilde{\sigma }}$ are mutually singular, then

$$\begin{aligned} \tilde{\tau }(y)=\frac{d \textbf{T}_i}{d\mu }(y)\qquad \text {for } \mu \text {-almost every }y\in U(x_i,r_i). \end{aligned}$$

Hence

43 $$\begin{aligned} \begin{aligned} \int |\tilde{\tau }(y)-\tau (y)|d\mu (y)=&\sum _{i=1}^\infty \int _{U(x_i,r_i)} |\tilde{\tau }(y)-\tau (y)|d\mu (y) \\ \le&\sum _{i=1}^\infty \int _{U(x_i,r_i)}|\tilde{\tau }(y)-\tau (x_i)|d\mu (y)+\varepsilon \mathbb {M}(\mu )\\ =&\sum _{i=1}^\infty \int _{U(x_i,r_i)}\Big |\frac{d\textbf{T}_i}{d\mu }(y)-\tau (x_i)\Big |d\mu (y)+\varepsilon \mathbb {M}(\mu ). \end{aligned} \end{aligned}$$

Inequality (41) can be rewritten, thanks to (42), as44Putting together (43) and (44) we conclude that

$$\begin{aligned} \int |\tilde{\tau }(y)-\tau (y)|d\mu (y)\le 2\varepsilon \mathbb {M}(\mu ). \end{aligned}$$

Finally, thanks to the choice of $\varepsilon >0$, we have

$$\begin{aligned} \mathbb {M}(\textbf{T})\le (4\mathbb {M}(\mu )+1)\varepsilon +2\Vert \tau \Vert _{L^1(\mu )}\le 2(1+2\varepsilon _0)\Vert \tau \Vert _{L^1(\mu )}. \end{aligned}$$

$$\square $$

### Proposition 5.4

Let $\mu$ be a finite measure on $\mathbb {G}$ and suppose $\tau$ is an $L^1(\mu )$ vector field such that $\tau (x)\in N(\mu ,x)$ for $\mu$-almost every $x\in \mathbb {G}$. Then there exists an horizontal normal current $\textbf{T}$ on $\mathbb {G}$ such that

(i) the Radon-Nikodym derivative of $\textbf{T}$ with respect to $\mu$ coincides $\mu$-almost everywhere with $\tau$, that is $\textbf{T}=\tau \mu +\varvec{\sigma }$ where $\sigma$ and $\mu$ are mutually singular;
(ii) $$\partial \textbf{T}=0$$ and $$\mathbb {M}(\textbf{T})\le 4\Vert \tau \Vert _{L^1(\mu )}$$.

### Proof

The proof of the proposition follows verbatim that of [3, Proposition 6.3] where we replace [3, Lemma 6.11] with Lemma 5.3. $\square$

### Remark 5.2

Note that if we substitute to item (a) of Definition 3.2 the assumption

- (a**) each $\mu _t$ is absolutely continuous with respect to the restriction of $\mathscr {H}^1$ to the image of a fragment $\gamma _t\in \mathfrak {F}$ such that $\gamma _t$ is 2-bi-Lipschitz as well, i.e. $d_c(\gamma _t(\sigma ),\gamma _t(\tau ))\ge |\sigma -\tau |/2$ for every $\sigma ,\tau \in \textrm{dom}(\gamma _t)$.

then the notion of decomposability bundle does not change. Denote by $$V^{**}(\mu ,\cdot )$$ the decomposability bundle arising from the assumption (a**) and items (b) and (c) of Definition 3.2. Note that thanks to Remark 3.1 the inclusion $$ V^{**}(\mu ,\cdot )\subseteq V(\mu ,\cdot )$$ holds $$\mu $$-almost everywhere. Therefore, we just need to check the converse. In other words, for every family of measures $$\mu _t$$ satisfying (a), (b) and (c) we need to show that

45 $$\begin{aligned} \mathfrak {v}_{\gamma _t}(x)\subset V^{**}(\mu ,x) \quad \text {for } \mu _t\text {-almost every}~x\text { and almost every~}t\in I. \end{aligned}$$

However this is an immediate consequence of Step 1 of the proof of Lemma A.6.

The following is the Carnot counterpart of [3, Theorem 6.4]:

### Theorem 5.5

Let $\mu$ be a Radon measure on $\mathbb {G}$. Then, for $\mu$-almost every $x\in \mathbb {G}$ we have $V_1\cap V(\mu ,x)=\pi _1(N(\mu ,x))$.

### Proof

Let us first prove the inclusion $\pi _1(N(\mu ,x))\subseteq V_1\cap V(\mu ,x)$. Assume by contradiction that the inclusion does not hold on a set of positive $\mu$-measure. Then, by [51, Theorem 5.2.1] we can find a bounded Borel vector field $\tau :\mathbb {G}\cong \mathbb {R}^n\rightarrow \mathbb {R}^n$ such that $\pi _1(\tau (x))\in \pi _1(N(\mu ,x))\setminus V_1\cap V(\mu ,x)$ on a set of positive $\mu$-measure. Note that $\pi _1(\tau (x))\in \pi _1(N(\mu ,x))$ is equivalent to $\tau (x)\in N(\mu ,x)$ and therefore Proposition 5.4 can be applied. Note further that here we will make use of the measurability of $N(\mu ,x)$ provided by Lemma Lemma 5.1. Thanks to Proposition 5.4 there exists an horizontal 1-dimensional normal current $\textbf{T}$ such that $\textbf{T}=\tau \mu +\varvec{\sigma }$ where $\varvec{\sigma }$ and $\mu$ are mutually singular. Thanks to Proposition 3.6, we know that $\pi _1(\tau (x))\in V(\mu ,x)\cap V_1$ for $\mu$-almost every $x\in \mathbb {G}$ which is in contradiction with the choice of $\tau$.

Let us prove the converse inclusion. Denoting by *M* the universally measurable set of those $x\in \mathbb {G}$ where $\textrm{dim}(N(\mu ,x))=n_1$, thanks to the above discussion, we infer that $V_1=\pi _1(N(\mu ,x))\subseteq V_1\cap V(\mu ,x)=V_1$ for $\mu$-almost every $x\in M$. The locality of $V(\mu ,x)$, proved in Proposition 3.5 and that of $N(\mu ,x)$, that is apparent from its very definition, allow us to assume without loss of generality that $\textrm{dim}(N(\mu ,x))\equiv k<n_1$ for $\mu$-almost every $x\in \mathbb {G}$. Throughout the rest of the proof we will identify without further mention the measure space (*I*, *dt*) with $(\mathbb {R},\mathscr {L}^1)$ thanks to [3, Remark 2.7 (iii)]. In addition, since $x\mapsto N(\mu ,x)$ is universally measurable we can assume, up to modifying it on a $\mu$-null set, that $x\mapsto N(\mu ,x)$ is Borel. Thanks to Remark 5.2, we just need to show that for every family of measures $t\mapsto \mu _t$ that satisfies items (a), (b) and (c) of Definition 3.2 with the further constraint that the 1-Lipschitz fragments $\gamma _t$ such that  are 2-bi-Lipschitz, we have that46where we recall that $\mathfrak {v}_\gamma$ was introduced in Lemma 2.9. Let us assume by contradiction that there is such a family for which (46) fails. Let $\Theta$ be a family of one-sided cones cones $C=C(e,\alpha )\subseteq V_1$ with *e* ranging in a given countable dense subset of the unit sphere in $V_1$ and $\alpha$ ranging in a given countable dense subset of (0, 1) and define for every such $C\in \Theta$ the sets

$$\begin{aligned} F_C:= &   \big \{x\in \mathbb {G}:C\cap \pi _1(N(\mu ,x))=\{0\}\big \}\qquad \text {and}\\ \mathcal {T}_{C}:= &   \{(t,x)\in \mathbb {R}\times F_C:\mathfrak {v}_{\gamma _t(x)}\in C\setminus \{0\}\}. \end{aligned}$$

Let us discuss the Borelianity of such sets. First of all, let us note that $x\mapsto \pi _1(N(\mu ,x))$ is easily seen Borel, as $x\mapsto N(\mu ,x)$ is Borel. Further, $(t,x)\mapsto \mathfrak {v}_{\gamma _t(x)}$ is seen to be a Borel map thanks to Lemma A.6. The Borelianity of such maps directly implies the Borelianity of $F_C$ and $\mathcal {T}_C$ and observe that thanks to our reduction we have $\mu (\mathbb {G}\setminus \cup _{C\in \Theta }F_C)=0$. Let us note that the map , where $\delta _t$ is the Dirac delta at *t*, is easily seen to be Borel and therefore, we can define $\nu _C$ as the Radon measure on $\mathbb {G}$ that acts as

$$\begin{aligned} \nu _C(E):=\int \delta _t\otimes \mu _t(\mathcal {T}_C\cap (\mathbb {R}\otimes E))\,dt\qquad \text {for every Borel set }E\subseteq \mathbb {G}. \end{aligned}$$

In addition, for every Borel set $E\subseteq \mathbb {G}$ we get for $\nu _C$, the representationwhere $\mathcal {G}_{C,t}:=\{x\in F_C : \mathfrak {v}_{\gamma _t(x)}\in C\setminus \{0\}\}$. Note further that $\nu _C(F_C^c)=0$ and that the map  is Borel.

Let us check that our contradiction assumption implies that there exists $C_\flat \in \Theta$ such that $\nu _{C_\flat }$ is non-trivial. Suppose by contradiction that for every $C\in \Theta$ we have $\nu _C=0$. This implies that for every $C\in \Theta$ and for almost every $t\in I$ we have $\delta _t\otimes \mu _t(\mathcal {T}_C)=0$ and, in particular,

47 $$\begin{aligned}  &   \text {for every }C\in \Theta ,\text { almost every }t\in I\text { and for } \mu _t\text {-almost every }x\in F_C\text { we have }\nonumber \\  &   \quad \mathfrak {v}_{\gamma _t}(x)\not \in C, \end{aligned}$$

where we can exclude that $\mathfrak {v}_{\gamma _t}(x)=0$ on a set of $\mu _t$-positive measure thanks to Lemma 2.9.

Lusin’s theorem and the Borelianity of $N(\mu ,x)$ tell us that for every $\varepsilon >0$ there exists a Borel set $G_\varepsilon$ such that $\mu (\mathbb {G}\setminus G_\varepsilon )<\varepsilon$ and such that $N(\mu ,x)$ is continuous on $G_\varepsilon$. In order to fix notations for every $e\in V_1$ and $\sigma \in (0,1)$ we let $X(e,\sigma ):=C(e,\sigma )\cup C(-e,\sigma )$. Since $N(\mu ,\cdot )$ is supposed to have constant dimension *k* almost everywhere, we can write $G_\varepsilon$ as a disjoint countable union of Borel sets $A_j\subseteq \mathbb {G}$ such that for each $j\in \mathbb {N}$ there exists a *k*-dimensional plane $N_j$ of $V_1$ for which $\pi _1(N(\mu ,x))\in X(N_j,\varepsilon )$ for every $x\in A_j$, where

$$\begin{aligned} X(N_j,\varepsilon ):=V_1\setminus \bigcup \Big \{C(e,\sqrt{1-\varepsilon ^2}):e\text { is unitary and orthogonal to }N_j\text { in }V_1\Big \}. \end{aligned}$$

Let $\{w_1,\ldots ,w_{n_1-k}\}$ be a family of orthonormal vectors of $V_1$ orthogonal to $N_j$. Fix *j*, define $C_i:=C(w_i,\sqrt{1-\varepsilon ^2})$, and note that $A_j\subseteq \cup _{j=1}^{n_1-k}(F_{C_i}\cup F_{-C_i})$. This, together with (47), implies that

48 $$\begin{aligned}  &   \text {for almost every }t\in I\text { and for }\mu _t\text {-almost every }x\in A_j\text { we have }\nonumber \\  &   \quad \mathfrak {v}_{\gamma _t}(x)\not \in \bigcup _{i=1}^{n_1-j}(C_i\cup -C_i)\subseteq V_1\setminus X(N_j,4n_1\varepsilon ). \end{aligned}$$

This can be rephrased in the following more convenient way:

$$\begin{aligned}  &   \text {for almost every }t\in I\text { and }\mu _t\text {-almost every }x\in A_j\text { we have }\\  &   \quad \pi _1(N(\mu ,x))\cap C(\mathfrak {v}_{\gamma _t}(x),16n_1\varepsilon )\ne \{0\}. \end{aligned}$$

However, thanks to the arbitrariness of $\varepsilon >0$ we get a contradiction with our assumption that (46) fails. This proves the existence of a cone $C_\flat$ for which $\nu _{C_\flat }$ is non-trivial.

Since the fragments $\gamma _t$ are supposed to be bi-Lipschitz, we also infer thatTherefore by Lemma A.3(ii) we know that the map $t\mapsto \llbracket \gamma _t\vert _{\gamma _t^{-1}(\mathcal {G}_{C_\flat ,t})}\rrbracket =:\varvec{\mu }_{\flat ,t}$ is Borel and thanks to the fact that the $\gamma _t$s are bi-Lipschitz, we also infer thatThanks to Corollary 4.3 we can find a 1-dimensional horizontal normal current $\textbf{T}_\flat$ such that

$$\begin{aligned} \textbf{T}_{\flat }=\int _{I}\varvec{\mu }_{\flat ,t}\,dt+\varvec{\sigma }=:\varvec{\mu }_{\flat }+\varvec{\sigma }, \end{aligned}$$

where $\Vert \varvec{\mu }_{\flat }\Vert$ and  are mutually absoulutely continuous, $\partial \textbf{T}_{\flat }=0$ and $\textbf{T}_{\flat }=\varvec{\mu }_\flat +\varvec{\sigma }$, where $\varvec{\sigma }$ and $\mu$ are mutually singular and

49 $$\begin{aligned} \frac{d\textbf{T}_\flat }{d\Vert \textbf{T}_\flat \Vert }(x)\in \mathscr {C}(x)[C_\flat ]\setminus \{0\}\qquad \text {for }\Vert \varvec{\mu }_\flat \Vert \text {-almost every }x\in \mathbb {G}. \end{aligned}$$

Since $\Vert \varvec{\mu }_\flat \Vert$ and $\nu _{C_\flat }$ are mutually absolutely continuous, we infer by our choice of $C_\flat$ that $\Vert \varvec{\mu }_\flat \Vert$ is non-zero. In addition, since $\Vert \varvec{\mu }_\flat \Vert \ll \nu _{C_\flat }\ll \int \mu _t\,dt\ll \mu$ and $\Vert \varvec{\mu }_\flat \Vert (F_{C_\flat }^c)=0$, we infer that

$$\begin{aligned} \frac{d\textbf{T}_\flat }{d\Vert \textbf{T}_\flat \Vert }(x)\in \mathscr {C}(x)[C_\flat ]\setminus \{0\}\qquad \text { on a set of } \mu \text {-positive measure contained in }F_{C_\flat }. \end{aligned}$$

Thanks Proposition 5.2 we finally infer that $\frac{d\textbf{T}_\flat }{d\Vert \textbf{T}_\flat \Vert }(x)\in N(\mu ,x)$ on a set of $\mu$-positive measure contained in $F_{C_\flat }$. This however contradicts the definition of $F_{C_\flat }$ and we have reached our contradiction. $\square$

### Remark 5.3

Note that since by construction $N(\mu ,x)$ is contained in $\mathscr {C}(x)[V_1]$, we infer by Proposition 5.4 that $N(\mu ,x)=\mathscr {C}(x)[V_1\cap V(\mu ,x)]$ for $\mu$-almost every $x\in \mathbb {G}$.

## Differentiability Along the Decomposability Bundle

This section is devoted to the proof of Theorem 1.2. We shall remark here that the results proved in the present section are independent on the proof of Theorem 1.1. Finally, we remark that even though here we assume the target $\mathbb {H}$ to be a Carnot group, the proof of Theorem 1.2 extends to homogeneous groups, see Remark 6.2. *Throughout the rest of this section and if not otherwise specified*, $\mathbb {G}$ *and* $\mathbb {H}$ *will always be fixed Carnot groups endowed with a homogeneous and left invariant distance and we will always assume that the dimension of the first layer* $V_1$ *of the Lie algebra of* $\mathbb {G}$ is $n_1$.

### Construction of Vector Fields of Universal Differentiability

First of all let us introduce some notation.

#### Definition 6.1

A function $f:\mathbb {G}\rightarrow \mathbb {H}$ is said to have *derivative* $Df(x,\zeta )$ *at* *x* *along* $\zeta \in \mathbb {G}$ if the following limit exists:

$$\begin{aligned} Df(x,\zeta )=\lim _{r\rightarrow 0+}\delta _{1/r}(f(x)^{-1}*f(x\delta _r(\zeta )))\in \mathbb {G}. \end{aligned}$$

Furthermore, *f* is said to be *differentiable at the point* $x\in \mathbb {G}$ *along* $\zeta \in \mathbb {G}$, if $Df(x,\zeta )$ and $Df(x,\zeta ^{-1})$ exist and $Df(x,\zeta )^{-1}=Df(x,\zeta ^{-1})$.

#### Remark 6.1

Note that if $Df(x,\zeta )$ exists, then for every $\lambda >0$ the derivative $Df(x,\delta _\lambda (\zeta ))$ of *f* along $\delta _{\lambda }(\zeta )$ at *x* exists and $Df(x,\delta _\lambda (\zeta ))=\delta _\lambda Df(x,\zeta )$. Indeed

$$\begin{aligned} \lim _{r\rightarrow 0+}\delta _{1/r}(f(x)^{-1}f(x\delta _{\lambda r}(\zeta )))= &   \delta _{\lambda }\Big (\lim _{r\rightarrow 0+}\delta _{1/\lambda r}(f(x)^{-1}f(x\delta _{\lambda r}(\zeta )))\Big )\\  = &   \delta _\lambda (Df(x,\zeta )). \end{aligned}$$

Finally, note that a Lipschitz function $f:\mathbb {G}\rightarrow \mathbb {H}$ is Pansu differentiable at $x\in \mathbb {G}$ along a subgroup $V\in \textrm{Gr}(\mathbb {G})$, see Definition 2.7 if and only if $Df(x,\zeta )$ exists for all $\zeta \in V$ and $\zeta \mapsto Df(x,\zeta )$ is an homogeneous homomorphism on *V*.

#### Proposition 6.1

([9, Proposition 2.10]) Let $\mathcal {L}(\mathbb {G},\mathbb {G})$ be the set of linear maps from the vector space underlying $\mathbb {G}$ into itself, endowed with the operator norm. Then, the following are equivalent:

(i) $V:\mathbb {G}\rightarrow \textrm{Gr}_{\textrm{eu}}(\mathbb {G})$ is a Borel map, where $\textrm{Gr}_{\textrm{eu}}(\mathbb {G})$ was introduced in Definition 2.4;
(ii) the projection map $\pi _V:\mathbb {G}\rightarrow \mathcal {L}(\mathbb {G},\mathbb {G})$, defined as $\pi _V(x):=\Pi _{V(x)}$ where $\Pi _{V(x)}$ is the Euclidean orthogonal projection onto *V*(*x*), is Borel;
(iii) the projection map $\pi _{V^\perp }:\mathbb {G}\rightarrow \mathcal {L}(\mathbb {G},\mathbb {G})$ defined as $\pi _{V^\perp }(x):=\Pi _{V(x)^\perp }$ where $\Pi _{V^\perp (x)}$ is the Euclidean orthogonal projection onto $V^\perp (x)$, the Euclidean orthogonal space to *V*(*x*), is Borel.

Finally the Borelianity of $\pi _V$ is also equivalent to saying that for every $v,w\in \mathbb {G}$, seen as vectors of coordinates, the map $x\mapsto \langle v,\pi _V(x)[w]\rangle$ is Borel.

#### Proof

The proof of the proposition is omitted. It can be achieved by proving that the map $\Psi$ associating an element of the Grassmannian $V\in \textrm{Gr}_{\textrm{eu}}(\mathbb {G})$ to its Euclidean orthogonal projection $\Pi _V$ is an homeomorphism. Actually what can be shown is that $\Psi$ is bi-Hölder. $\square$

This subsection is devoted to the proof of the following:

#### Lemma 6.2

Let $\mu$ be a Radon measure on $\mathbb {G}$. Then, there are $n_1$ Borel maps $\zeta _1,\dots ,\zeta _{n_1}:\mathbb {G}\rightarrow V_1$ such that:

(i) $V(\mu ,x)=\mathfrak {S}(\{\zeta _1(x),\dots ,\zeta _{n_1}(x)\})$ for $\mu$-almost every $x\in \mathbb {G}$,
(ii) every $f\in \textrm{Lip}(\mathbb {G},\mathbb {H})$ is differentiable at *x* along $\zeta _i(x)$ for every $i=1,\ldots ,n_1$ and for $\mu$-almost every $x\in \mathbb {G}$.

#### Proof

For every $i=1,\ldots ,n_1$ define

$$\begin{aligned} \zeta _i(x):={\left\{ \begin{array}{ll}\frac{\pi _{V(\mu ,\cdot )}(x)[e_i]}{|\pi _{V(\mu ,\cdot )}(x)[e_i]|}&  \text {if } \pi _{V(\mu ,\cdot )}(x)[e_i]\ne 0,\\ 0&  \text {otherwise}.\end{array}\right. }\qquad \text {and}\qquad w_i(x):=\mathscr {C}(x)[\zeta _i(x)]. \end{aligned}$$

where the $e_i$s are the vectors of Definition 2.5 and the map $\pi _{V(\mu ,\cdot )}$ is the projection map associated to $V(\mu ,\cdot )$ yielded by Proposition 6.1. Note that $\mu$-almost every $x\in \mathbb {G}$ the vectors $\zeta _i(x)$ are contained $V(\mu ,x)\cap V_1$ and since $e_1,\ldots ,e_{n_1}$ are orthonormal, the Borel vector fields $\zeta _i$ span $V(\mu ,x)\cap V_1$ at $\mu$-almost every $x\in \mathbb {G}$. Furthermore, by Remark 2.3 for $\mu$-almost every $x\in \mathbb {G}$ on the one hand we have that the vector fields $w_1,\ldots ,w_{n_1}$ span the vector space $\mathscr {C}(x)[V(\mu ,x)\cap V_1]$ and we also have that the identity $\pi _1[w_i(x)]=\zeta _i(x)$ holds at every $x\in \mathbb {G}$. Further, let us note that by Theorem 5.5 that $w_1,\ldots ,w_{n_1}\in N(\mu ,x)$ for $\mu$-almost every $x\in \mathbb {G}$. Therefore, for every $i=1,\ldots ,n_1$ we can apply Proposition 5.4 to get horizontal normal 1-currents without boundary $\textbf{T}_i=\tau _i\eta _i=\tau _i\mu +\varvec{\sigma }_i$, where $\varvec{\sigma }_i$ and $\mu$ are mutually singular, and such that $\tau _i=w_i$ for $\mu$-almost every $x\in \mathbb {G}$.

Thanks to Theorem 2.10 for every *i* we can find a family of vector-valued measures $t\mapsto \varvec{\eta }_t^i$ satisfying the hypothesis (a) and (b) of Definition 2.11 and such that $\textbf{T}_i$ can be written as $\textbf{T}_i=\int _I \varvec{\eta }_t^i\, dt$. Thanks to Theorem 2.10 we infer that for every *i* and every *t* there exists a Lipschitz curve $\gamma _t^i:[0,1]\rightarrow \mathbb {G}$ such that $\varvec{\eta }_t^i=\llbracket \gamma _t^i\rrbracket$ and $\mathfrak {v}_{\gamma _t}$ and $\tau _i$ coincide up to a non-zero scalar for $\Vert \llbracket \gamma _t^i\rrbracket \Vert$-almost every $x\in \mathbb {G}$. It is elementary to see that every Lipschitz map $f\in \textrm{Lip}(\mathbb {G},\mathbb {H})$ is Pansu differentiable along $\pi _1(\tau _i)$ for $\Vert \llbracket \gamma _t^i\rrbracket \Vert$-almost every $x\in \mathbb {G}$ and almost every $t\in I$. This implies in particular that every $f\in \textrm{Lip}(\mathbb {G},\mathbb {H})$ is Pansu differentiable along $\pi _1(\tau _i)$ for $\int \Vert \llbracket \gamma _t^i\rrbracket \Vert \, dt$-almost every $x\in \mathbb {G}$. However, since by Theorem 2.10 we have that $\Vert {\varvec{T}}_i\Vert =\int \Vert \llbracket \gamma _t^i \rrbracket \Vert \, dt$ and that $\pi _1(\tau _i)=\pi _1(w_i)=\zeta _i$ for $\mu$-almost every $x\in \mathbb {R}^n$ we conclude that every $f\in \textrm{Lip}(\mathbb {G},\mathbb {H})$ is Pansu differentiable along $\zeta _i$ for $\mu$-almost every $x\in \mathbb {R}^n$. Thanks to Proposition 2.4 and the fact that $\zeta _1,\ldots \zeta _{n_1}$ span $V(\mu ,\cdot )\cap V_1$ $\mu$-almost everywhere, the proof of the lemma is achieved. $\square$

### Partial and Total Derivatives

In this subsection we relate the existence of partial derivatives to the Pansu differentiability along the decomposability bundle. Since the group operation is not commutative, we cannot follow the proof of the Euclidean counterpart, see [3, Section 3].

#### Proposition 6.3

Let $\mu$ be a Radon measure on $\mathbb {G}$ and $\zeta :\mathbb {G}\rightarrow \mathbb {G}$ be a Borel map such that any Lipschitz map $f:\mathbb {G}\rightarrow \mathbb {H}$ is differentiable $\mu$-almost everywhere along $\zeta (\cdot )$. Finally let *B* be any $\mu$-positive Borel subset of $\textrm{supp}(\mu )$. Then, for $\mu$-almost every $x\in B$, we have

$$\begin{aligned} \lim _{t\rightarrow 0}\frac{\textrm{dist}_c(B,x*\delta _t(\zeta (x)))}{t}=0. \end{aligned}$$

More precisely there exists a $t(x)>0$ and a map $x(\cdot ): (-t(x),t(x))\rightarrow B$ such that

50 $$\begin{aligned} \lim _{t\rightarrow 0}\frac{d_c(x(t),x*\delta _t(\zeta (x)))}{t}=0. \end{aligned}$$

#### Proof

Since in any Carnot group there is an isometrically embedded copy of $\mathbb {R}$ if we prove the claim for $\mathbb {H}=\mathbb {R}$, the result follows in full generality. The first step of the proof is to show that the function $g(x):=\inf \{r>0:\mu (B(x,r)\cap B)>0\}$ is a non-negative 1-Lipschitz function. Note that $g(x)=0$ for $\mu$-almost every $x\in B$. Let $x,y\in \mathbb {G}$ and note that $B(y,r)\subseteq B(x,r+d(x,y))$. Therefore, for every $\varepsilon >0$ we have

51 $$\begin{aligned} \mu (B(x,g(y)+d(x,y)+\varepsilon )\cap B)\ge \mu (B(y,g(y)+\varepsilon )\cap B)>0. \end{aligned}$$

Inequality (51) implies that $g(x)\le g(y)+d(x,y)$ and thus, interchanging *x* and *y*, *g* is seen 1-Lipschitz. Suppose by contradiction that there is a $\mu$-positive compact set $K\subseteq B$ for which (50) fails everywhere on *K*. This means that for every $x\in K$ there is an infinitesimal sequence $s_i(x)$ and a $\lambda (x)>0$ such that

52 $$\begin{aligned} \textrm{dist}(x*\delta _{s_i(x)}(\zeta (x)),B)\ge \lambda (x) s_i(x)\qquad \text {for every }i\in \mathbb {N}. \end{aligned}$$

In order to discuss why (52) is false, we shall fix a $z\in K$ where $g(z)=0$ and note that (52) implies that

53 $$\begin{aligned} \limsup _{r\rightarrow 0}\frac{\textrm{dist}\big (z*\delta _{r}(\zeta (z)),B\big )}{|r|}\ge \lambda (z). \end{aligned}$$

We can also assume without loss of generality that *g* is differentiable along $\zeta (z)$ at *z*. Therefore, since we are assuming that $Dg(z,\zeta (z))=-Dg(z,\zeta (z)^{-1})$, we infer that

$$\begin{aligned} Dg(z,\zeta (z))=\lim _{r\rightarrow 0}\frac{g(z*\delta _r(\zeta (z)))-g(z)}{|r|}=\limsup _{r\rightarrow 0}\frac{g(z*\delta _r(\zeta (z)))}{|r|}\ge \lambda (z). \end{aligned}$$

This, together with the fact that *g* is non-negative implies that *g* cannot be differentiable along $\zeta (z)$ at *z*, since the identity $Dg(z,\zeta (z))=-Dg(z,\zeta (z)^{-1})$ cannot be satisfied even if both $Dg(z,\zeta (z))$ and $Dg(z,\zeta (z)^{-1})$ existed. The Borel regularity of the measure $\mu$ yields the desired conclusion. $\square$

#### Proposition 6.4

Suppose $\mu$ is a Radon measure on $\mathbb {G}$ and assume $\zeta _1,\zeta _2:\mathbb {G}\rightarrow \mathbb {G}$ are two Borel vector fields such that every $f\in \textrm{Lip}(\mathbb {G},\mathbb {H})$ is differentiable along both $\zeta _1(x)$ and $\zeta _2(x)$ for $\mu$-almost every $x\in \mathbb {G}$. Then, $\mu$-almost everywhere, every *f* is differentiable along $\zeta _{i_1}(x)^{\beta _1}\zeta _{i_2}(x)^{\beta _2}$, where $i_j\in \{1,2\}$ and $\beta _j\in \{\pm 1\}$ as $j=1,2$. Furthermore, we have:

54 $$\begin{aligned} Df(x,\zeta _{i_1}(x)^{\beta _1}\zeta _{i_2}(x)^{\beta _2})=Df(x,\zeta _{i_1}(x))^{\beta _1}Df(x,\zeta _{i_2}(x))^{\beta _2}. \end{aligned}$$

#### Proof

Without loss of generality we can assume that the measure $\mu$ is supported on a compact set *K*. Therefore, thanks to Severini-Egoroff’s theorem and Lusin’s theorem we can find a compact set $K_1$ such that:

(i) $$\mu (K\setminus K_1)\le \varepsilon \mu (K)$$,
(ii) the incremental ratios $Rf(x,\zeta _i(x);t):=\delta _{1/t}(f(x)^{-1}f(x*\delta _t(\zeta _i(x))))$ converge uniformly to $Df(x,\zeta _i)$ on $K_1$ as *t* goes to 0 for $i=1,2$,
(iii) the maps $\zeta _i(\cdot )$ and $Df(x,\zeta _i(x)^\beta )$ are continuous on $K_1$ for every $i=1,2$ and $\beta \in \{\pm 1\}$.

Let $\beta _1,\beta _2\in \{\pm 1\}$ and $i_1,i_2\in \{1,2\}$ and note that

55 $$\begin{aligned} Rf(x,\zeta _{i_1}(x)^{\beta _1}\zeta _{i_2}(x)^{\beta _2};t)=Rf(x,\zeta _{i_1}(x)^{\beta _1};t)*Rf(x*\delta _t(\zeta _{i_1}(x))^{\beta _1},\zeta _{i_2}(x)^{\beta _2};t). \nonumber \\ \end{aligned}$$

By (ii) we immediately infer that $\lim _{t\rightarrow 0}Rf(x,\zeta _{i_1}(x)^{\beta _1};t)=Df(x,\zeta _{i_1}(x)^{\beta _1})$. This implies in particular that in order to conclude the proof of the proposition, we just need to show that:

56 $$\begin{aligned} \lim _{t\rightarrow 0+}Rf(x*\delta _t(\zeta _{i_1}(x))^{\beta _1},\zeta _{i_2}(x)^{\beta _2};t)=Df(x,\zeta _{i_2}(x)^{\beta _2}). \end{aligned}$$

Thanks to Proposition 6.3, for $\mu$-almost every $x\in K_1$ we can find a map *x*(*t*) taking values in $K_1$ for which

57 $$\begin{aligned} \lim _{t\rightarrow 0+}\frac{d_c(x(t),x\delta _t(\zeta _{i_1}(x)^{\beta _1}))}{t}=0. \end{aligned}$$

With the aid of the map *x*(*t*), we can rewrite $Rf(x\delta _t(\zeta _{i_1}(x))^{\beta _1},\zeta _{i_2}(x)^{\beta _2};t)$ as follows:

58 $$\begin{aligned} \begin{aligned}&Rf(x*\delta _t(\zeta _{i_1}(x))^{\beta _1},\zeta _{i_2}(x)^{\beta _2};t)\\&\quad =\underbrace{\delta _{1/t}\Big (f\big (x*\delta _t(\zeta _{i_1}(x)^{\beta _1})\big )^{-1}f(x(t))\Big )}_{\text {(I)}}* \underbrace{Rf\left( x(t),\zeta _{i_2}(x)^{\beta _2};t\right) }_{\text {(II)}}*\\&\quad \underbrace{\delta _{1/t}\Big (f\big (x(t)*\delta _t(\zeta _{i_2}(x)^{\beta _1}\big )^{-1}f\left( x*\delta _{t}(\zeta _{i_1}(x)^{\beta _1}*\zeta _{i_2}(x)^{\beta _2}\right) \Big )}_{\text {(III)}}. \end{aligned} \end{aligned}$$

Let us separately estimate the norm of the terms (I), (II) and (III). Using that *f* is Lipschitz we deduce that

59 $$\begin{aligned} \frac{\lim _{t\rightarrow 0+}\Vert \text {(I)}\Vert }{\text {Lip}(f)}\le \lim _{t\rightarrow 0+} \frac{d_c(x*\delta _t(\zeta _{i_1}(x)^{\beta _1}),x(t))}{t}=0. \end{aligned}$$

Furthermore, thanks to (57) we infer that

60 $$\begin{aligned} \begin{aligned} \frac{\lim _{t\rightarrow 0}\Vert \text {(III)}\Vert }{\text {Lip}(f)}\le&\lim _{t\rightarrow 0+} \frac{d\big (x(t)*\delta _t(\zeta _{i_2}(x)^{\beta _2}),x*\delta _{t}(\zeta _{i_1}(x)^{\beta _1}*\zeta _{i_2}(x)^{\beta _2})\big )}{t}\\ =&\lim _{t\rightarrow 0+} \Vert \zeta _{i_2}(x)^{-\beta _2}\delta _{1/t}\big (x(t)^{-1}*x*\delta _t(\zeta _{i_1}(x)^{\beta _1})\big )*\zeta _{i_2}(x)^{\beta _2}\Vert =0. \end{aligned} \end{aligned}$$

Finally, we can rewrite (II) in the following convenient way:

61 $$\begin{aligned}  &   \text {(II)}=Rf(x(t),\zeta _{i_2}(x(t))^{\beta _2};t)*\nonumber \\  &   \underbrace{\delta _{1/t}(f(x(t)*\delta _t(\zeta _{i_2}(x(t))^{\beta _2})^{-1}*f(x(t)*\delta _t(\zeta _{i_2}(x)^{\beta _2}))}_{\text {(IV)}}. \nonumber \\ \end{aligned}$$

Thanks to (ii) and the fact that $x(t)\in K_1$, for every $\varepsilon >0$ there exists a $t_\varepsilon >0$ such that

$$\begin{aligned} \Vert Df(x(t),\zeta _{i_2}(x(t))^{\beta _2})^{-1} Rf(x(t),\zeta _{i_2}(x(t))^{\beta _2};t)\Vert \le \varepsilon , \end{aligned}$$

for every $|t|\le t_\varepsilon$. Finally, the Lipschitzianity of *f* and (iii) imply that

62 $$\begin{aligned} \lim _{t\rightarrow 0+}\Vert \text {(IV)}\Vert \le \text {Lip}(f)\lim _{t\rightarrow 0+}\Vert \zeta _{i_2}(x(t))^{-\beta _2}\zeta _{i_2}(x)^{\beta _2}\Vert =0. \end{aligned}$$

Putting together the information we gathered, we infer that

$$\begin{aligned} \begin{aligned}&\limsup _{t\rightarrow 0+}\Vert Df(x,\zeta _{i_2}(x)^{\beta _2})^{-1}*Rf(x*\delta _t(\zeta _{i_1}(x)),\zeta _{i_2}(x)^{\beta _2};t)\Vert \\&\quad \underset{(58),(61)}{=}\limsup _{t\rightarrow 0+}\Vert Df(x,\zeta _{i_2}(x)^{\beta _2})^{-1}*\mathrm {(I)}*Df(x,\zeta _{i_2}(x)^{\beta _2})*Df(x,\zeta _{i_2}(x)^{\beta _2})^{-1}\\&\qquad *Rf(x(t),\zeta _{i_2}(x(t))^{\beta _2};t)*\mathrm {(IV)}*\mathrm {(III)}\Vert \\&\quad \underset{(59),(60),(62)}{=}\limsup _{t\rightarrow 0+}\Vert Df(x,\zeta _{i_2}(x)^{\beta _2})^{-1}*Rf(x(t),\zeta _{i_2}(x(t))^{\beta _2};t)\Vert \\&\quad \le \limsup _{t\rightarrow 0+}\Vert Df(x,\zeta _{i_2}(x)^{\beta _2})^{-1}*Df(x(t),\zeta _{i_2}(x(t))^{\beta _2})\Vert +\Vert Df(x(t),\zeta _{i_2}(x(t))^{\beta _2})^{-1}\\&\qquad *Rf(x(t),\zeta _{i_2}(x(t))^{\beta _2};t)\Vert \le \varepsilon , \end{aligned} \end{aligned}$$

where in the last identity we also used Lemma 2.3 and where the last inequality above comes from (iii). The arbitrariness of $\varepsilon$ concludes the proof. $\square$

#### Theorem 6.5

Suppose $\mathscr {D}$ is a finite family of Borel maps $\zeta :\mathbb {G}\rightarrow \mathbb {G}$ such that any $f\in \textrm{Lip}(\mathbb {G},\mathbb {H})$ is differentiable at $\mu$-almost every $x\in \mathbb {G}$ along $\zeta (x)$. Then, every Lipschitz map $f\in \textrm{Lip}(\mathbb {G},\mathbb {H})$ is Pansu differentiable with respect to the subgroup $\mathfrak {S}(\{\zeta (x):\zeta \in \mathscr {D}\})$ for $\mu$-almost any $x\in \mathbb {G}$ .

#### Proof

Let $v:\mathbb {G}\rightarrow \mathbb {G}$ be a map for which there exists an $N\in \mathbb {N}$, $\rho _i\in \mathbb {Q}$ and $v_i\in \mathscr {D}$ with $i=1,\ldots ,N$ such that

63 $$\begin{aligned} v(x)=\delta _{\rho _1}(v_1(x))*\cdots *\delta _{\rho _N}(v_N(x)). \end{aligned}$$

Let $\tilde{\mathfrak {S}}$ be the countable family of maps that satisfy identity (63) for some choice of *N*, $\rho _i$ and $v_i$ and let

$$\begin{aligned} \tilde{\mathfrak {S}}(x):=\{w\in \mathbb {G}:\text {there exists a }v\in \tilde{\mathfrak {S}}\text { such that }v(x)=w\}. \end{aligned}$$

Proposition 6.4 and Remark 6.1 immediately imply that for $\mu$-almost every $x\in \mathbb {G}$ every Lipschitz map is differentiable along *v*(*x*) whenever $v\in \tilde{\mathfrak {S}}$ and

64 $$\begin{aligned}  &   Df(x,u(x)v(x))=Df(x,u(x))Df(x,v(x))\qquad \nonumber \\  &   \quad \text {for }\mu \text {-almost every }x\in \mathbb {G}\text { and any }u,v\in \tilde{\mathfrak {S}}. \end{aligned}$$

In particular this can be rephrased as follows. For $$\mu $$-almost every $$x\in \mathbb {G}$$, every Lipschitz map is differentiable along any $$v\in \tilde{\mathfrak {S}}(x)$$ and $$ Df(x,u*v)=Df(x,u)Df(x,v)$$ for $$\mu $$-almost every $$x\in \mathbb {G}$$ and any $$u,v\in \tilde{\mathfrak {S}}(x)$$.

The next step in the proof is to show that for $\mu$-almost every $x\in \mathbb {G}$ and any $w\in \textrm{cl}(\tilde{\mathfrak {S}}(x))$ every Lipschitz function is differentiable along *w* at *x*. Thanks to the choice of *w* there exists a Cauchy sequence $\{w_i\}_{i\in \mathbb {N}}\subseteq \tilde{\mathfrak {S}}(x)$ such that for every $k\in \mathbb {N}$ there exists an $M\in \mathbb {N}$ such that for every $i,j\ge M$ we have $d_\mathbb {G}(w_j,w_i)\le 1/k$. Since $w_i^{-1}w_j\in \tilde{\mathfrak {S}}(x)$, thanks to (64) we infer that

65 $$\begin{aligned} \begin{aligned} d_\mathbb {H}(Df(x,w_i),Df(x,w_j))=&d_\mathbb {H}(Df(x,w_i^{-1}*w_j),0)\\ =&\lim _{t\rightarrow 0+} \frac{d_\mathbb {H}\big (f(x)^{-1}*f(x*\delta _t(w_i^{-1}*w_j)),0\big )}{t}\\ \le&\textrm{Lip}(f)d_\mathbb {G}(w_j,w_i)\le \textrm{Lip}(f)/k. \end{aligned}\nonumber \\ \end{aligned}$$

for every $i,j\ge M$. The bound (65) shows that the sequence $\{Df(x,w_i)\}_{i\in \mathbb {N}}$ is Cauchy in $\mathbb {H}$ and thus there exists an element of $\mathbb {H}$, that we denote by $\mathfrak {d}f(x,w)$, such that $\lim _{i\rightarrow \infty }Df(x,w_i)=\mathfrak {d}f(x,w)$. However, for every $i\in \mathbb {N}$ we have that

66 $$\begin{aligned} \begin{aligned}&\limsup _{t\rightarrow 0+}\frac{\Vert \delta _t(\mathfrak {d}f(x,w))^{-1}*f(x)^{-1}*f(x*\delta _t(w))\Vert _\mathbb {H}}{t}\\&\quad \le \Vert Df(x,w_i)^{-1}*\mathfrak {d}f(x,w)\Vert _\mathbb {H}\\&\qquad +\limsup _{t\rightarrow 0+}\frac{\Vert \delta _t(Df(x,w_i))^{-1}*f(x)^{-1}*f(x*\delta _t(w_i))\Vert _\mathbb {H}}{t}\\&\qquad +\limsup _{t\rightarrow 0+}\frac{\Vert f(x*\delta _t(w_i))^{-1}*f(x*\delta _t(w)) \Vert _\mathbb {H}}{t}\\&\quad \le \Vert Df(x,w_i)^{-1}*\mathfrak {d}f(x,w)\Vert _\mathbb {H}+\textrm{Lip}(f)d_\mathbb {G}(w_i,w). \end{aligned}\nonumber \\ \end{aligned}$$

The arbitrariness of *i* implies that

$$\begin{aligned} \mathfrak {d}f(x,w)=\lim _{t\rightarrow 0+}\delta _{1/t}(f(x)^{-1}* f(x*\delta _r(w))=Df(x,w), \end{aligned}$$

and this shows that *f* is differentiable at *x* along *w*. Note in particular that the above computations also prove that the function $Df(x,\cdot ):\textrm{cl}(\tilde{\mathfrak {S}}(x))\rightarrow \mathbb {H}$ is continuous.

Since it can be easily seen that $\mathfrak {S}(\{v(x):v\in \mathscr {D}\})=\textrm{cl}(\tilde{\mathfrak {S}}(x))$ for every $x\in \mathbb {G}$, the only thing left to prove is that the map $v\mapsto Df(x,v)$ is a homogeneous homomorphism on $\textrm{cl}(\tilde{\mathfrak {S}}(x))$. To do to this, let $v,w\in \textrm{cl}(\tilde{\mathfrak {S}}(x))$ and let $v_i,w_i\subseteq \tilde{\mathfrak {S}}(x)$ be two sequence converging to *v* and *w* respectively. Since the sequence $v_i*w_i\in \text {cl}(\tilde{\mathfrak {S}}(x))$ converges to $v*w$, by the continuity of $Df(x,\cdot )$, we infer that

$$\begin{aligned} Df(x,v*w)= &   \lim _{i\rightarrow \infty }Df(x,v_i*w_i)=\lim _{i\rightarrow \infty }Df(x,v_i)*Df(x,w_i)\\= &   Df(x,v)*Df(x,w). \end{aligned}$$

This concludes the proof, since the homogeneity of $Df(x,\cdot )$ is guaranteed by Remark 6.1. $\square$

#### Theorem 6.6

Let $\mu$ be a Radon measure on $\mathbb {G}$. Then, for every Carnot group $\mathbb {H}$ and for $\mu$-almost every $x\in \mathbb {G}$ every Lipschitz map $f\in \textrm{Lip}(\mathbb {G},\mathbb {H})$ is differentiable along the subgroup $V(\mu ,x)\in \textrm{Gr}_\mathfrak {C}(\mathbb {G})$, the decomposability bundle of $\mu$ defined in Definition 3.2, for $\mu$-almost every $x\in \mathbb {G}$.

#### Proof

The Theorem follows immediately from Lemma 6.2, which guarantees that every Lipschitz function admits directional derivatives along a family of Borel vector fields $\zeta _1,\dots ,\zeta _{n_1}:\mathbb {G}\rightarrow \mathbb {G}$ generating the decomposability bundle $V(\mu ,x)$ at $\mu$-almost every *x*, and Theorem 6.5 guarantees that these directional derivatives give rise to the Pansu differentiability with respect to the decomposability bundle. $\square$

#### Remark 6.2

Here below we list some observation on Theorem 6.6 and its proof.

1. Theorem 6.6 holds even if we suppose that $\mathbb {H}$ is just an homogeneous group.
  Indeed, let $\mathbb {H}'=\mathfrak {S}(V_1(\mathbb {H}))$, where $V_1(\mathbb {H})$ is the first layer of the Lie algebra of $\mathbb {H}$ and where $\mathfrak {S}$ was introduced in Definition 2.3. Let us remark that even though $\mathfrak {S}$ was just introduced in Carnot groups, its definition makes perfect sense in general homogeneous groups. Also, it can be easily checked that $\mathbb {H}'$ is a Carnot group, as by definition its Lie algebra is generated by the first layer.
  Let $\gamma$ be a Lipschitz curve connecting $0\in \mathbb {G}$ to any point $w\in \mathbb {G}$ and note that $f(0)^{-1}*f\circ \gamma$ is a Lipschitz curve in $\mathbb {H}$ connecting 0 to $f(0)^{-1}*f(w)$. It is not hard to see, for instance by approximating $\gamma$ with Lipschitz curves that are piece-wise flow lines of horizontal vector fields, that this implies that $f(0)^{-1}*f\circ \gamma$ must be contained in $\mathbb {H}'$ and hence $f(0)^{-1}*f(w)\in \mathbb {H}'$. This actually shows that $f(0)^{-1}*f(\mathbb {G})\subseteq \mathbb {H}'$ and hence $f(0)^{-1}*f$ can be seen as a Lipschitz map from $\mathbb {G}$ to $\mathbb {H}'$. Since a function $f\in \textrm{Lip}(\mathbb {G},\mathbb {H})$ is differentiable along $V(\mu ,x)$ if and only if $f(0)^{-1}*f$ is differentiable along $V(\mu ,x)$, applying Theorem 6.6 to $f(0)^{-1}*f$ the differentiability of *f* along $V(\mu ,x)$ is thus proved.
2. Further, with few modifications to the proofs, the statement of Theorem 6.6 can be strengthened to the following localized form.
  *Let *$\mu$ *be a Radon measure on* $\mathbb {G}$ *and* $B\subseteq \mathbb {G}$ *be a Borel set. Then, for every homogeneous group* $\mathbb {H}$ *and for* $\mu$-*almost every* $x\in \mathbb {G}$ *every Lipschitz map* $f\in \textrm{Lip}(B,\mathbb {H})$ *is differentiable along* $V(\mu ,x)$, *for* $\mu$-*almost every* $x\in B$, *i.e.*
    $$\begin{aligned} \lim _{B\ni y\rightarrow x} \frac{\Vert df(x)[x^{-1}y]^{-1}f(x)^{-1}f(y)\Vert _\mathbb {H}}{d_c(x,y)}=0, \end{aligned}$$
    *for some homogeneous homomorphism* $df(x):V\rightarrow \mathbb {H}$.
  where here $\textrm{Lip}(B,\mathbb {H})$ denotes the family of Lipschitz maps $f:B\subseteq \mathbb {G}\rightarrow \mathbb {H}$. This is a non-trivial extension as it is well known that maps between general Carnot groups do not enjoy any extension property, see for instance [12, Theorem 1].
3. At this stage it is not clear whether the decomposability bundle constructed here is sharp in the sense that on the directions *v* on $\mathbb {G}$ not contained in $V(\mu ,x)$ there are Lipschitz function $f:\mathbb {G}\rightarrow \mathbb {R}$ which are non-differentiable along *v* at *x*, compare with [3, Theorem 1.1(ii)]. It seems however plausible that the same techniques employed in [3] might yield the existence of a Lipschitz function $f\in \textrm{Lip}(\mathbb {G},\mathbb {R})$ such that *f* is non differentiable along any $v\in \mathbb {G}\setminus (V(\mu ,x)\cup \text {exp}( V_2\oplus \ldots \oplus V_s))$ for $\mu$-almost every $x\in \mathbb {G}$. This will be subject to further investigation.
  Finally, it is a simple observation to note, see for instance [30, Remark 1.2], that there are measures $\mu$ for which $V(\mu ,x)$ is the largest subspace of differentiability for Lipschitz functions, in the following sense: if $V:\mathbb {G}\rightarrow \textrm{Gr}(\mathbb {G})$ is a Borel map such that for every Carnot group $\mathbb {H}$ every $f\in \textrm{Lip}(\mathbb {G},\mathbb {H})$ is differentiable $\mu$-almost everywhere along *V*(*x*), then we have $V(x)\subseteq V(\mu ,x)$ for $\mu$-almost every $x\in \mathbb {G}$.

## The Reverse of Pansu’s Theorem

### Decompositions of a Measure Satisfying Pansu’s Theorem

#### Definition 7.1

(Lipschitz chart) Let $(X,d,\mu )$ be a metric measure space. Let $U\subset X$ be a Borel set, and let $\varphi :X\rightarrow \mathbb {R}^n$ be a Lipschitz function. We say that $(U,\varphi )$ is a *Lipschitz chart with target* $\mathbb {R}^n$, or simply *chart*, when the following holds. Every Lipschitz function $f:X\rightarrow \mathbb {R}$ is *differentiable* $\mu$-almost everywhere in $(U,\varphi )$; i.e., for $\mu$-almost every $x_0\in U$ there exists a unique linear map $Df(x_0):\mathbb {R}^n\rightarrow \mathbb {R}$ such that

67 $$\begin{aligned} \limsup _{X\ni x\rightarrow x_0}\frac{|f(x)-f(x_0)- Df(x_0)[\varphi (x)-\varphi (x_0)]|}{d(x,x_0)}=0. \end{aligned}$$

#### Definition 7.2

(Lipschitz differentiability space) A metric measure space $(X,d,\mu )$ is said to be a *Lipschitz differentiability space* if there exist Borel sets $U_i\subset X$ such that $\mu (X\setminus \cup _{i\in \mathbb {N}}U_i)=0$, an $N\in \mathbb {N}$, and Lipschitz functions $\varphi _i:X\rightarrow \mathbb {R}^{n_i}$ with $n_i\le N$, such that $(U_i,\varphi _i)$ is a chart for every $i\in \mathbb {N}$.

#### Definition 7.3

We say that a Carnot group $\mathbb {G}$ endowed with a Radon measure $\mu$ has the *Pansu property* with respect to a Carnot group $\mathbb {H}$ if for every Lipschitz function $f:\mathbb {G}\rightarrow \mathbb {H}$ and for $\mu$-almost every $x_0\in \mathbb {G}$ there exists a homogeneous homomorphism $\text {d}f(x_0):\mathbb {G}\rightarrow \mathbb {H}$ such that

68 $$\begin{aligned} \limsup _{x\rightarrow x_0}\frac{d_\mathbb {H}(f(x),f(x_0)*\text {d}f(x_0)[x_0^{-1}x])}{d_c(x,x_0)}=0. \end{aligned}$$

#### Remark 7.1

Since $df(x_0):\mathbb {G}\rightarrow \mathbb {R}$ is a group homomorphism then, thanks to [25, Proposition 2.5], for every $g\in \mathbb {G}$ we have $df(x_0)[g]=df(x_0)[\pi _1(g)]$.

#### Remark 7.2

Suppose $\mu$ is a Radon measure on $\mathbb {G}$ with the Pansu property with respect to some homogeneous group $\mathbb {H}$. Let $g:\mathbb {G}\rightarrow \mathbb {R}$ be a Lipschitz map and *e* be an element of the first layer $V_1$ of $\mathbb {G}$. It is easily seen that the map $f:\mathbb {G}\rightarrow \mathbb {H}$ defined as $f(x):=\delta _{g(x)}(e)$ is Lipschitz and

69 $$\begin{aligned} \begin{aligned} 0&=\limsup _{x\rightarrow x_0}\frac{\Vert \text {d}f(x_0)[x_0^{-1}x]^{-1}f(x_0)^{-1}f(x)\Vert _\mathbb {H}}{d_c(x,x_0)}\\&=\limsup _{x\rightarrow x_0}\frac{\Vert \text {d}f(x_0)[x_0^{-1}x]^{-1}\delta _{g(x)-g(x_0)}(e)\Vert _\mathbb {H}}{d_c(x,x_0)}. \end{aligned} \end{aligned}$$

This shows in particular that for every $v\in \mathbb {G}$ we have

$$\begin{aligned} \lim _{r\rightarrow 0}\frac{g(x_0\delta _r(v))-g(x_0)}{r} e=\textrm{d}f(x_0)[v]. \end{aligned}$$

Therefore, the image of the homogeneous homomorphism $\textrm{d}f(x_0)$ is contained in the 1-parameter subgroup generated by *e*. This immediately shows together with Remark 7.1 that $\textrm{d}f(x_0)=\delta _{\langle L(x_0),\pi _1(x_0^{-1}x)\rangle }( e)$, where $L(x_0)$ is a suitable element of $V_1$. It is thus immediate to see that defined $\textrm{d}g(x_0):=\langle L(x_0),\pi _1(x_0^{-1}x)\rangle$ we have

$$\begin{aligned} \limsup _{x\rightarrow x_0}\frac{|g(x)-g(x_0)-\textrm{d}g(x_0)[x_0^{-1}x]|}{d_c(x,x_0)}=0. \end{aligned}$$

This shows that in order to prove Theorem 1.1 it is sufficient to restrict ourselves to the case where $\mathbb {H}$ is the real line and that the definition of the Pansu property with real-valued functions is the weakest possible.

#### Proposition 7.1

Suppose the Carnot group $\mathbb {G}$ endowed with the measure $\mu$ has the Pansu property. Then, $(\mathbb {G},d,\mu )$ is a Lipschitz differentiability space with the global chart $\pi _1:\mathbb {G}\rightarrow V_1$.

#### Proof

Thanks to Remark 7.1, for every Lipschitz function $f:\mathbb {G}\rightarrow \mathbb {R}$ and $\mu$-almost every $x\in \mathbb {G}$ we have

$$\begin{aligned} \begin{aligned} 0&=\limsup _{x\rightarrow x_0}\frac{|f(x)-f(x_0)-\textrm{d}f(x_0)[x_0^{-1}x]|}{d_c(x,x_0)}\\&=\limsup _{x\rightarrow x_0}\frac{|f(x)-f(x_0)-\textrm{d}f(x_0)[\pi _1(x_0^{-1}x)]|}{d_c(x,x_0)}\\&=\limsup _{x\rightarrow x_0}\frac{|f(x)-f(x_0)-\textrm{d}f(x_0)[\pi _1(x)-\pi _1(x_0)]|}{d_c(x,x_0)}. \end{aligned} \end{aligned}$$

The above computation shows that the hypothesis of the axioms of Lipschitz differentiability space are satisfied by $(\mathbb {G},d,\mu )$ with the global chart $\pi _1:\mathbb {G}\rightarrow V_1$. $\square$

#### Proposition 7.2

([16, Theorem 9.5]) Suppose that $(X,d,\mu )$ is a Lipschitz differentiability space and assume that $(U,\varphi )$ is an *n*-dimensional chart. Let $w\in \mathbb {S}^{n-1}$ and $0< \varepsilon <1$. Then, there is a family of measures $t\mapsto \mu _t$ satisfying the hypothesis (a) and (b) of Definition 2.11 and such that

(i) for almost every $t\in I$ there exists a bi-Lipschitz fragment $\gamma _t$ defined on a compact set $K_t$ of $\mathbb {R}$ such that  and
(ii) for almost every $t\in I$ and almost every $s\in K_t$ we have $(\varphi \circ \gamma _t)^\prime (s)\in C(w,\varepsilon )$.

#### Corollary 7.3

Assume $\mu$ is a Radon measure on $\mathbb {G}$ with the Pansu property. Then, for every $e\in \mathbb {S}^{n_1-1}$ there is a family of measures $t\mapsto \mu _t$ satisfying the hypothesis (a) and (b) of Definition 2.11 and such that

(i) for almost every $t\in I$ there exists a bi-Lipschitz fragment $\gamma _t$ defined on a compact set $K_t$ of $\mathbb {R}$ such that  and
  $$\begin{aligned} \mu =\int \mu _t\,dt. \end{aligned}$$
(ii) for almost every $t\in I$ and almost every $s\in K_t$ we have $D\gamma _{t}(s)=(\pi _1\circ \gamma _t)^\prime (s)\in C(e,\varepsilon )$.

#### Proof

Thanks to Proposition 7.1 we know that $(\mathbb {G},d_c,\mu )$ is a Lipschitz differentiability space and that $(\mathbb {G},\pi _1)$ is a $n_1$-dimensional chart. Finally, Proposition 7.2 immediately yields the conclusion. $\square$

#### Proposition 7.4

Assume $\mu$ is a Radon measure on $\mathbb {G}$ with the Pansu property. Then

(i) $V(\mu ,x)=\mathbb {G}$ for $\mu$-almost every $x\in \mathbb {G}$,
(ii) for any $j=1,\ldots ,n_1$ we can find a 1-dimensional horizontal normal current $\textbf{T}_{j}=\tau _{j}\eta _{j}$ with $\partial \textbf{T}_{j}=0$ and such that $\mu \ll \eta _{j}$ and
  $$\begin{aligned} \tau _{j}(x)=\mathscr {C}(x)[e_j]\qquad \text {for }\mu \text {-almost every } x\in \mathbb {G}, \end{aligned}$$
  where as usual $\{e_1,\ldots ,e_{n_1}\}$ denotes an orthonormal basis of $V_1$.

#### Proof

Let $\{e_1,\ldots ,e_{n_1}\}$ be an orthonormal basis of $V_1$. Thanks to Corollary 7.3, for every $j=1,\ldots ,n_1$ there is a family of measures $t\mapsto \mu _{j,t}$ satisfying the hypothesis (a) and (b) of Definition 2.11 and such that

- ($\alpha$) for almost every $t\in I$ there exists a bi-Lipschitz fragment $\gamma _{j,t}$ defined on a compact set $K_{j,t}$ of $\mathbb {R}$ such that  and $\mu =\int \mu _{j,t}\,dt$;
- ($\beta$) for almost every $t\in I$ and almost every $s\in K_{j,t}$ we have $D\gamma _{t}^j(s)=(\pi _1\circ \gamma _{j,t})^\prime (s)\in C(e_j,\varepsilon )$.

Without loss of generality we can assume that . This can be seen by arguing as in the proof of (7) and (8). With this assumption, we note that $\mu _{j,t}=\Vert \llbracket \gamma _{j,t}\rrbracket \Vert$, thanks to the bi-Lipschitzianity of $\gamma _{j,t}$s. This, together with Lemma A.3(ii) implies that for every $j=1,\ldots , n_1$ we have that the map $t\mapsto \llbracket \gamma _{j,t}\rrbracket$ is Borel and satisfies items (a) and (b) of Definition 2.11. In addition, thanks to ($\beta$), all the hypothesis of Corollary 4.3 are satisfied. Therefore, Corollary 4.3 implies that for every $j=1,\ldots ,n_1$ and for every $\varepsilon _0>0$ the measures $\mu$ and $\Vert \int _I \llbracket \gamma _{j,t}\rrbracket \,dt\Vert$ are mutually absolutely continuous and there are horizontal normal 1-current $\textbf{T}_j=\tau _j\eta _j$ on $\mathbb {G}$ such that $\partial \textbf{T}_j=0$, $\textbf{T}_j=\int _I\llbracket \gamma _{j,t}\rrbracket +\varvec{\sigma }$, where $\varvec{\sigma }$ and $\sum _{j=1}^{n_1}\int _I\Vert \llbracket \gamma _{j,t}\rrbracket \Vert \,dt$ are mutually singular and

$$\begin{aligned} \tau _j(x)\in \mathscr {C}(x)[C(e_j,\varepsilon _0)]\setminus \{0\}\qquad \text {for } \Big \Vert \int _I\llbracket \gamma _{j,t}\rrbracket \,dt\Big \Vert \text {-almost every }x\in \mathbb {G}. \end{aligned}$$

However, since $\mu$ and $\Vert \int _I\llbracket \gamma _{j,t}\rrbracket \,dt\Vert$ are mutually absolutely continuous thanks to Proposition 3.6 we infer that

$$\begin{aligned} \mathfrak {S}(\{\tau _1(x),\ldots ,\tau _{n_1}(x)\})\subseteq V(\mu ,x)\qquad \text {for }\mu \text {-almost every }x\in \mathbb {G}. \end{aligned}$$

However, chosen $\varepsilon _0$ small we infer that the vectors $\tau _1(x),\ldots ,\tau _{n_1}(x)$ of $V_1$ are independent $\mu$-almost everywhere. This implies by Lemma 3.3 that $V(\mu ,x)=\mathbb {G}$ for $\mu$-almost every $x\in \mathbb {G}$. This concludes the proof of (i). In order to conclude the proof of (ii) it suffices to directly apply Proposition 5.4 and Theorem 5.5. $\square$

We are now ready to prove the main result, which states that the existence of $n_1$ independent representations for a Radon measure $\mu$ in a Carnot group $\mathbb {G}$ implies that $\mu$ is diffuse. This is the analogue of [21, Corollary 1.12] and the proof follows the same overall strategy of [21, Theorem 1.1], which was in turn inspired by the strong constancy lemma of Allard [5]. As explained in the introduction, we have however to adapt the proof to the “hypoelliptic setting”. As additional difficulties, we note that in this context we can not rely on a Besicovitch covering theorem and some classical Lebesgue point arguments need to be adapted. For the sake of readability we report these proofs in the appendix.

#### Proposition 7.5

Suppose $\mu$ is a Radon measure on $\mathbb {G}$ satisfying (ii) in Proposition 7.4. Then $\mu \ll \mathscr {L}^n$.

An immediate consequence of the above proposition is our main result which is

#### Theorem 7.6

Let $\mathbb {G}, \mathbb {H}$ be Carnot groups. Suppose further that $\mu$ is a Radon measure on $\mathbb {G}$ with the Pansu property with respect to $\mathbb {H}$. Then $\mu \ll \mathscr {L}^n$.

#### Proof

The claim follows immediately from Propositions 7.5, 7.4 and Remark 7.2. $\square$

#### Proof of Proposition 7.5

We divide the proof in several steps.

(i) notations. Thanks to Proposition 7.4 we know that

- ($*$) for any $j=1,\ldots ,n_1$ we can find a 1-dimensional horizontal normal current $\textbf{T}_{j}=\tau _{j}\eta _{j}$ with $\partial \textbf{T}_{j}=0$ and such that $\mu \ll \eta _{j}$ and $\tau _{j}(x) =\mathscr {C}(x)[e_j]\text { for }\mu \text {-almost every }x\in \mathbb {G}.$

Thanks to Remark 2.8, we can think of $\textbf{T}_j$ as a vector-valued measure $\textbf{T}_j\in \mathcal {M}(\mathbb {G},\mathbb {R}^{n_1})$ acting by duality with the scalar product of $\mathbb {R}^{n_1}$ on the smooth function $\omega \in C^\infty (\mathbb {G},\mathbb {R}^{n_1})$ and the boundary operator $\partial$ on these currents acts as shown in (10) and (11). Thus, the 1-currents $\textbf{T}_1,\ldots ,\textbf{T}_{n_1}$ above can be written in this notation as $\textbf{T}_j=e_j\eta _j$ for every $j=1,\ldots ,n_1$.

Throughout the proof, we define on the measures $\varvec{\nu }=(\varvec{\nu }_1,\ldots ,\varvec{\nu }_{n_1})\in \mathcal {M}(\mathbb {G},\mathbb {R}^{n_1\times n_1})$, i.e. the Radon measures taking values in $\mathbb {R}^{n_1\times n_1}$, the differential operator $\mathfrak {B}$ that acts as

$$\begin{aligned} \langle \mathfrak {B}\varvec{\nu },\varphi \rangle =(\langle \varvec{\nu }_1,d_H\varphi _1\rangle ,\ldots ,\langle \varvec{\nu }_{n_1},d_H\varphi _{n_1}\rangle ),\\ \qquad \text {on every test function }C^\infty (\mathbb {G},\mathbb {R}^{n_1}\times \mathbb {R}^{n_1}), \end{aligned}$$

where $d_H$ is as in (11). In the above notations, if we let $\mathbb {T}:=(\textbf{T}_1,\ldots ,\textbf{T}_{n_1})$ we easily see that $\mathfrak {B}\mathbb {T}=0$. Indeed, thanks to (10) and using the representation $\textbf{T}_j=e_j\eta _j$, we have

$$\begin{aligned} \langle \mathfrak {B}\mathbb {T}\, ; \, \varphi \rangle= &   (\langle e_1\eta _1\, ; \, d_H\varphi _1\rangle ,\ldots ,\langle e_{n_1}\eta _{n_1}\, ; \, d_H\varphi _{n_1}\rangle )\\= &   (\langle \partial \textbf{T}_1\, ; \, \varphi _1\rangle ,\ldots ,\langle \partial \textbf{T}_{n_1}\, ; \, \varphi _{n_1}\rangle )=0. \end{aligned}$$

In addition, we can write $\mathbb {T}$ as

$$\begin{aligned} \mathbb {T}=(\tau _1\eta _1,\ldots , \tau _{n_1}\eta _{n_1})=\mathfrak {T}\Xi =\mathfrak {T}\Xi ^a+\mathfrak {T}\Xi ^s, \end{aligned}$$

where $\mathfrak {T}:\mathbb {G}\rightarrow \mathbb {R}^{n_1\times n_1}$ is a Borel map in $L^1(\Xi )$ such that $|\mathfrak {T}|=1$ for $\Xi$-almost every $x\in \mathbb {G}$, $\Xi ^a\ll \mathscr {L}^n$ and $\Xi ^s$ is mutually singular with respect to $\mathscr {L}^n$. Here, given an $n_1\times n_1$ matrix *A* the norm $|A|$ is computed as follows: denoted by $a_1,\ldots ,a_{n_1}\in \mathbb {R}^{n_1}$ the columns of *A* we let $|A|^2:=n_1^{-1}\sum _{i=1}^{n_1}|a_i|^2$, where $|a_i|$ denotes the usual Euclidean norm of the vectors $a_i$. Note that since for every $j=1,\ldots ,n_1$ we have $\Xi \ge \eta _j$ and this shows in particular that $\mu \ll \Xi$.

(ii) localisation at a singular point. In the following, we will show by contradiction that the conditions $x_0\in \textrm{supp}(\Xi ^s)$ and the fact that $\mathfrak {T}(x_0)$ is invertible, are incompatible, or more precisely that the conditions (i)-(iv) below cannot hold together on a set of positive $\Xi ^s$-measure. Let us assume by contradiction that there exists an $x_0\in \textrm{supp}(\Xi ^s)$ for which there exists an infinitesimal sequence $r_k$ such that

(i)
(ii) $\displaystyle \limsup _{k\rightarrow \infty }\frac{\Xi (B(x_0,r_k/5))}{\Xi (B(x_0,r_k))} \ge \frac{2}{j_0}$ for some $j_0 \in \mathbb {N}$;
(iii) $$\displaystyle \lim _{k\rightarrow \infty }\frac{\Xi ^a(B(x_0, r_k ))}{\Xi ^s(B(x_0,r_k))} = 0$$;
(iv) $P_0:=\mathfrak {T}(x_0)=\textrm{diag}(\kappa _1,\ldots ,\kappa _{n_1})$ for some $\kappa _1,\ldots ,\kappa _{n_1}\in \mathbb {R}\setminus \{0\}$.

The contradiction that will prove the theorem will arise from the fact that the item (iv), that holds thanks to ($*$), cannot hold on a set of positive $\Xi ^s$ measure. Essentially, the fact that that $P_0$ is invertible has two consequences. First, it allows us to promote the weak* convergence $\Xi ^{-1}(B(x_0,r_k))T_{x_0,r_k}\mathbb {T}\rightharpoonup \varvec{\nu }$, to a stronger convergence in mass. Secondly, it will force $\varvec{\nu }\ll \mathscr {L}^n$. Therefore, the strong convergence in mass to $\nu$ will force $\Xi ^s$ to not be singular obtaining a contradiction, as $x_0$ was chosen to be a density point for $\Xi ^s$. This will be shown in the last line of the proof of the theorem, under (93).

First of all, let us note that Proposition B.2 that (i), (ii) and (iii) hold $\Xi ^s$-almost everywhere. Define the normalized blow-up sequence

70 $$\begin{aligned} \varvec{\nu }_k : = \frac{1}{\Xi (B(x_0,r_k))} T_{x_0,r_k}\mathbb {T}, \qquad \text {for every } k \in \mathbb {N}. \end{aligned}$$

and note that $\Vert \varvec{\nu }_k\Vert (B(0,1) )=1$ and $\liminf _{k\rightarrow \infty }\Vert \varvec{\nu }_k\Vert (B(0,1/5) ) \ge j_0^{-1} > 0$ for *k* sufficiently big. Without loss of generality we can assume that this holds for every $k\in \mathbb {N}$. Up to the extraction of a subsequence, by (ii) we can assume that

71 $$\begin{aligned} \Vert \varvec{\nu }_k\Vert \overset{*}{\rightharpoonup }\nu \quad \text {in }\mathscr {M}_+(B(0,1)) \end{aligned}$$

with $\nu (B(0,1) )\le 1$ and $\nu (B(0,1/5) ) \ge j_0^{-1}$. The vector fields $X_i$ are left-invariant, and thus their (formal) adjoints coincide with $-X_j$, and this implies that

72 $$\begin{aligned} \begin{aligned} \mathfrak {B}(\varvec{\mu }_1,\ldots ,\varvec{\mu }_{n_1})&=-\left( \sum _{i=1}^{n_1}X_i\left( \varvec{\mu }_1^i\right) ,\ldots ,\sum _{i=1}^{n_1}X_i\Big (\varvec{\mu }_{n_1}^i\Big )\right) ,\\&\quad \text {with } \left( \varvec{\mu }_1,\ldots ,\varvec{\mu }_{n_1}\right) \in \mathscr {M}(\mathbb {R}^n,\mathbb {R}^{n_1}), \end{aligned} \end{aligned}$$

where $\varvec{\mu }_j^i$ denotes the *i*th entry of $\varvec{\mu }_j$. In addition, since the vector fields $X_j$ are homogeneous, we also have

$$\begin{aligned} X_j\varphi (\delta _{1/r}(x^{-1}y)) = rX_j(\varphi (\delta _{1/r}(x^{-1}\cdot )))(y), \end{aligned}$$

for every $j=1,\ldots ,n_1$, every smooth functions $\varphi$ and every $r > 0$. This, together with an elementary computation shows in particular that $\mathfrak {B}[\varvec{\nu }_k]=0$ for every $k\in \mathbb {N}$. Then $\mathfrak {B}[P_0\Vert \varvec{\nu }_k\Vert ]=-P_0^T[\nabla _\mathbb {G}\Vert \varvec{\nu }_k\Vert ]$ where $\nabla _\mathbb {G}:=(X_1,\ldots ,X_{n_1})$. On the other hand,

73 $$\begin{aligned} \mathfrak {B}[P_0\Vert \varvec{\nu }_k\Vert ]=\mathfrak {B}[P_0\Vert \varvec{\nu }_k\Vert -\varvec{\nu }_k]+\mathfrak {B}\varvec{\nu }_k=\mathfrak {B}[P_0\Vert \varvec{\nu }_k\Vert -\varvec{\nu }_k]. \end{aligned}$$

Let $\Phi$ be a smooth positive function supported on *B*(0, 1) such that $\int \Phi d\mathscr {L}^n=1$ and $\Phi (\cdot )=\Phi (\cdot ^{-1})=\Phi (-\cdot )$. Let $\{\varepsilon _k\}_{k\in \mathbb {N}}$ be an infinitesimal sequence of positive real numbers to be fixed later, let $\Phi _{\varepsilon _k}(\cdot ):=\varepsilon _k^{-\mathcal {Q}}\Phi (\delta _{1/\varepsilon _k}(\cdot ))$ and define that

74 $$\begin{aligned} \begin{aligned} u_k&:= \Phi _{\varepsilon _k} * \Vert \varvec{\nu }_k\Vert \in C^\infty (B(0,1)), \\ V_k&:= \Phi _{\varepsilon _k} * \bigl [ P_0 \Vert \varvec{\nu }_k\Vert - \varvec{\nu }_k \bigr ] \in C^\infty (B(0,1),\mathbb {R}^{n_1\times n_1}), \end{aligned} \end{aligned}$$

where here $*$ denotes the convolution with respect to the group law of $\mathbb {G}$, i.e. $f*g:=\int f(xy^{-1})g(y)d\mathscr {L}^n(y)$. It will be clear from the context when $*$ denotes a convolution and when it denotes the group law of $\mathbb {G}$. Then, if we let $\chi \in C^\infty (\mathbb {G},[0,1])$ be such that $\chi =1$ on *B*(0, 1/2) and $\chi =0$ on $B(0,3/4)^c$, we infer from the above discussions that

75 $$\begin{aligned} \begin{aligned}&-P_0^T[\nabla _\mathbb {G}(\chi u_k)]=\mathfrak {B}[P_0\chi u_k]=-u_kP_0^T[\nabla _\mathbb {G}\chi ]-\chi P_0^T[\nabla _\mathbb {G}u_k]\\&\quad =-u_kP_0^T[\nabla _\mathbb {G}\chi ]-\chi P_0^T[\nabla _\mathbb {G}(\Phi _{\varepsilon _k}*\Vert \varvec{\nu }_k\Vert )]\\&\quad =-u_kP_0^T[\nabla _\mathbb {G}\chi ]-\chi P_0^T[\Phi _{\varepsilon _k}*\nabla _\mathbb {G}\Vert \varvec{\nu }_k\Vert ]=-u_kP_0^T[\nabla _\mathbb {G}\chi ]-\chi \Phi _{\varepsilon _k}*P_0^T[\nabla _\mathbb {G}\Vert \varvec{\nu }_k\Vert ]\\&\quad =-u_kP_0^T[\nabla _\mathbb {G}\chi ]+\chi \Phi _{\varepsilon _k}*\mathfrak {B}[P_0\Vert \varvec{\nu }_k\Vert ]\\&\quad =-u_kP_0^T[\nabla _\mathbb {G}\chi ]+\chi \Phi _{\varepsilon _k}*\mathfrak {B}[P_0\Vert \varvec{\nu }_k\Vert -\varvec{\nu }_k]+\chi \Phi _{\varepsilon _k}*\mathfrak {B}[\varvec{\nu }_k]\\&\quad =-u_kP_0^T[\nabla _\mathbb {G}\chi ]+\chi \Phi _{\varepsilon _k}*\mathfrak {B}[P_0\Vert \varvec{\nu }_k\Vert -\varvec{\nu }_k]. \end{aligned}\nonumber \\ \end{aligned}$$

Thanks to (72) and to the fact that for every $i=\{1,\ldots ,n_1\}$ we have $\langle X \psi _1,\varphi \rangle =-\langle \psi _1, X \varphi \rangle$ and $X(\psi _1*\psi _2)=\psi _1*X\psi _2$ for every distribution $\psi _1,\psi _2$ and every test function $\varphi$. It is possible to prove that

$$\begin{aligned} \Phi _{\varepsilon _k}*\mathfrak {B}[P_0\Vert \varvec{\nu }_k\Vert -\varvec{\nu }_k]=\mathfrak {B}[\Phi _{\varepsilon _k}*(P_0\Vert \varvec{\nu }_k\Vert -\varvec{\nu }_k)], \end{aligned}$$

and hence (75) can be rewritten as

76 $$\begin{aligned} -P_0^T[\nabla _\mathbb {G}(\chi u_k)]=-u_kP_0^T[\nabla _\mathbb {G}\chi ]+\chi \mathfrak {B}[V_k]\nonumber \\ =-u_kP_0^T[\nabla _\mathbb {G}\chi ]-V_k\mathfrak {B}[\chi ] +\mathfrak {B}[\chi V_k]. \end{aligned}$$

Define

77 $$\begin{aligned} R_k:=-u_kP_0^T[\nabla _\mathbb {G}\chi ]-V_k\mathfrak {B}[\chi ], \end{aligned}$$

and let us apply to both sides of (76) the differential operator $-\nabla ^T_\mathbb {G} P_0$, to obtain

$$\begin{aligned} \nabla ^T_\mathbb {G} P_0P_0^T\nabla _\mathbb {G}[\chi u_k]=-\nabla ^T_\mathbb {G}P_0[\mathfrak {B}[\chi V_k]+R_k]=-\nabla ^T_\mathbb {G}P_0[\mathfrak {B}[\chi V_k]]-\nabla ^T_\mathbb {G}P_0[R_k]. \end{aligned}$$

The matrix $\Gamma :=P_0^TP_0=\textrm{diag}(\kappa _1^2,\ldots ,\kappa _{n_1}^2)$ is positively definite and diagonal. Therefore, the operator $\nabla ^T_\mathbb {G} P_0P_0^T\nabla _\mathbb {G}$ can thus be rewritten as

$$\begin{aligned} \mathfrak {D}:=\nabla ^T_\mathbb {G} P_0P_0^T\nabla _\mathbb {G}=\sum _{i=1}^{n_1}\kappa _i^2 X_i^2=\sum _{i=1}^{n_1}(|\kappa _i|X_i)^2. \end{aligned}$$

Since $\mathfrak {D}$ is a sub-Laplacian it is well known (see e.g. [17, Proposition 5.3.2] and [17, Proposition 5.3.11]) that $\mathfrak {D}$ admits a fundamental solution $K_0$ satisfying $K_0\in C^{\infty }(\mathbb {G}\setminus \{0\})$, $K_0\in L^1_{loc}(\mathbb {G})$ and $K_0(x)=K_0(-x)$. In addition $K_0$ is $(2-Q)$-homogeneous and hence the distribution $X_iK_0$ is $(1-Q)$-homogeneous for every $i=1,\ldots ,n_1$. Let us first note that

78 $$\begin{aligned} 0\le \chi u_k= &   -\nabla ^T_\mathbb {G}P_0[\mathfrak {B}[\chi V_k]]*K_0-\nabla ^T_\mathbb {G}P_0[R_k]*K_0\nonumber \\= &   \mathcal {L}_1[\chi V_k]*K_0+\mathcal {L}_2[R_k]*K_0=:f_k+g_k, \end{aligned}$$

where we note that the convolutions above are well defined in the pointwise sense since both $-\nabla ^T_\mathbb {G}P_0[\mathfrak {B}[\chi V_k]]$ and $-\nabla ^T_\mathbb {G}P_0[R_k]$ have compact support.

precompactness of $\{g_k\}$ In this paragraph we prove that the sequence of functions $g_k:=\mathcal {L}_2[R_k]*K_0$, defined in (78), is precompact in $L^1(B(0,1))$. Define the operator $F_2(u):=\mathcal {L}_2[u]*K_0$ on $u\in C^\infty _c(\mathbb {G},\mathbb {R}^{n_1})$. Note that for every test function $\varphi$ we have

$$\begin{aligned} \langle F_2(u),\varphi \rangle= &   \langle \mathcal {L}_2[u],\varphi *K_0^\vee \rangle =\langle \mathcal {L}_2[u],\varphi *K_0\rangle =\sum _{j=1}^{n_1}\langle \kappa _ju^j*(X_jK_0)^\vee ,\varphi \rangle \\= &   \Big \langle \Big (-\sum _{j=1}^{n_1} \kappa _ju^j*(X_jK_0)\Big ),\varphi \Big \rangle , \end{aligned}$$

where we denoted by $u^j$ the *j*th component of the map $u\in C^\infty _c(\mathbb {G},\mathbb {R}^{n_1})$, and we used repeatedly the fact that $K_0=K_0^\vee$, where $\Psi ^\vee$ denotes the distribution that acts as $\langle \Psi ^\vee ,\varphi \rangle =\langle \Psi , \varphi (-\cdot )\rangle$. Since $X_iK_0$ is an $(1-Q)$-homogeneous distribution, by [23, Proposition 1.8(i),(ii)] we know that $X_iK_0\in L^1_{loc}(\mathbb {G})$ and that the following identity holds:

$$\begin{aligned} F_2(u)(x)= &   \int \Big (-\sum _{j=1}^{n_1}\kappa _j u^j\left( xy^{-1}\right) \Big )(X_jK_0)(y)d\mathscr {L}^n(y)\\  &   \quad \text {for every }u\in C^\infty _c(\mathbb {G},\mathbb {R}^{n_1})\text { and }x\in \mathbb {G}. \end{aligned}$$

Thanks to [24, Proposition 6.2], we know that $F_1$ is of weak type $(1,Q/(Q-1))$, i.e. $F_1$ extends to a continuous linear operator from $L^1(\mathbb {G})$ to the weak $L^{Q/(Q-1)}(\mathbb {G})$ space $L^{Q/(Q-1),\infty }(\mathbb {G})$.

In order to prove that the sequence $\{g_k:k\in \mathbb {N}\}$ is precompact in $L^1(\mathbb {G})$, we will employ Kolmogorov-Riesz-Frechet theorem. First of all, we prove that $g_k$ is bounded in $L^1(B(0,1))$ and secondly we will prove equi-continuity in $L^1$. For the exact statement of the theorem we are employing we refer to [28, Corollary 8].

**Step 1: boundness.** First of all, let us check that the sequence $V_k$ converges to 0 in $L^1(B(0,3/4))$. Thanks to the choice of $\chi$, we have

79 $$\begin{aligned} \begin{aligned}&\int \chi (y)|V_k(y)|d\mathscr {L}^n(y)\le \int _{B(0,3/4)} |V_k(y)|d\mathscr {L}^n(y)\\&\quad =\int _{B(0,3/4)} |\Phi _{\varepsilon _k}*( P_0\Vert \varvec{\nu }_k\Vert -\varvec{\nu }_k)|(y) d\mathscr {L}^n(y)\\&\quad \le \int _{B(0,1)} |P_0-\mathfrak {T}_k(y)|d\Vert \varvec{\nu }_k\Vert ( y), \end{aligned} \end{aligned}$$

where $\varvec{\nu }_k=\mathfrak {T}_k\Vert \varvec{\nu }_k\Vert$. On the other hand, recalling the definition of $\varvec{\nu }_k$ in (70), we conclude that

80 $$\begin{aligned} \begin{aligned} \lim _{k\rightarrow \infty }\int _{B(0,3/4)} |V_k(y)|d\mathscr {L}^n(y)\overset{(79)}{\le }&\lim _{k\rightarrow \infty }\frac{\int _{B(0,1)} |P_0-\mathfrak {T}(x_0\delta _{r_k}(y))|dT_{x_0,r_k}\Xi (y)}{\Xi (B(x_0,r_k))}\\ \le&\lim _{k\rightarrow \infty }\frac{\int _{B(x,r_k)}|P_0-\mathfrak {T}(z)|d\Xi (z)}{\Xi (B(x_0,r_k))}=0, \end{aligned} \end{aligned}$$

which shows that $V_k\rightarrow 0$ in $L^1(B(0,3/4))$ and in turn $\chi V_k\rightarrow 0$ in $L^1(\mathbb {G})$ by our choice of $\chi$. Secondly, we prove that $R_k$ is a bounded sequence in $L^1(\mathbb {G})$. Let us now give a uniform upper bound on the $L^1(\mathbb {G},\mathbb {R}^{n_1})$ norm of the functions $R_k$. It is easy to see that

$$\begin{aligned} \begin{aligned}&\int |R_k|d\mathscr {L}^n \le \int _{B(0,3/4)} u_k|P_0^T[\nabla _\mathbb {G}\chi ]|+\int _{B(0,3/4)} |V_k||\mathfrak {B}[\chi ]|d\mathscr {L}^n\\&\quad \le \Vert P_0^T[\nabla _\mathbb {G}\chi ] \Vert _\infty \int _{B(0,3/4)} u_kd\mathscr {L}^n+ \Vert \mathfrak {B}[\chi ]\Vert _\infty \int _{B(0,3/4)}|V_k|d\mathscr {L}^n \\&\quad \le \Vert P_0^T[\nabla _\mathbb {G}\chi ] \Vert _\infty + \Vert \mathfrak {B}[\chi ]\Vert _\infty \int _{B(0,3/4)}|V_k|d\mathscr {L}^n, \end{aligned} \end{aligned}$$

where the first inequality comes from the very definition of $R_k$, see (77). The above discussion together with the fact that $V_k\rightarrow 0$ in $L^1(B(0,3/4))$ implies that

81 $$\begin{aligned} \limsup _{k\rightarrow \infty } \Vert R_k\Vert _{L^1(\mathbb {G},\mathbb {R}^{n_1})}\le \Vert P_0^T[\nabla _\mathbb {G}\chi ] \Vert _\infty + \Vert \mathfrak {B}[\chi ]\Vert _\infty =:M. \end{aligned}$$

Finally, from (81) and the fact that $F_1$ is of weak type $(1,Q/(Q-1))$, we infer that $g_k=F_2(R_k)$ is a sequence bounded in $L^{Q/(Q-1),\infty }(\mathbb {G})$. However, an elementary computation, shows that $g_k$ is also bounded in $L^1(B(0,1))$.

**Step 2: equi-continuity.** The second and final step to prove the precompactness of $\{g_k\}$ in $L^1(B(0,1))$ is to show that for every $\rho \in (0,1)$ the sequence of functions $F_2(R_k)$ are equi-continuous in $L^1(B(0,\rho ))$. In other words, we aim to prove that for every $\epsilon >0$ and every $\rho \in (0,1)$ we want to find $0<\eta <\rho /2$ such that

$$\begin{aligned} \Vert g_k(\cdot *h)-g_k(\cdot )\Vert _{L^1(B(0,\rho ))}\le \varepsilon \qquad \text {whenever }\Vert h\Vert \le \eta . \end{aligned}$$

It is immediate to see that whenever $u\in C^\infty _c(\mathbb {G},\mathbb {R}^{n_1})$ is a smooth function support in *B*(0, 1) we have

82 $$\begin{aligned}  &   \Vert F_2(u)(\cdot *h)- F_2(u)(\cdot )\Vert _{L^1(B(0,\rho ))}\nonumber \\  &   \quad \le \sum _{j=1}^{n_1}|\kappa _j|\underbrace{\Vert u^j*(X_jK_0)(\cdot *h)-u^j*(X_jK_0)(\cdot )\Vert _{L^1(B(0,\rho ))}}_{(\Delta _j)}. \end{aligned}$$

For every $j=1$ we now estimate $\Delta _j$. In order to make the notation more readable we will write *u* instead of $u^j$, so that

83 $$\begin{aligned} \begin{aligned}&\Vert u*(X_jK_0)(\cdot *h)-u*(X_jK_0)(\cdot )\Vert _{L^1(B(0,\rho ))} \\&\quad =\int _{B(0,\rho )} |u*(X_jK_0)(yh)-u*(X_jK_0)(y)|d\mathscr {L}^n(y) \\&\quad = \int _{B(0,\rho )} \Big |\int _{B(0,\rho )} u(z)\Big ((X_jK_0)(z^{-1}yh)-(X_jK_0)(z^{-1}y)\Big ) d\mathscr {L}^n(z)\Big |d\mathscr {L}^n(y)\\&\quad \le \int _{B(0,\rho )} \int _{B(0,\rho )} |u(z)||(X_jK_0)(z^{-1}yh)-(X_jK_0)(z^{-1}y)|d\mathscr {L}^n(z) d\mathscr {L}^n(y)\\&\quad = \int _{B(0,\rho )} |u(z)|\Big (\int _{B(0,\rho )} |(X_jK_0)(z^{-1}yh)-(X_jK_0)(z^{-1}y)|d\mathscr {L}^n(y)\Big )d\mathscr {L}^n(z). \end{aligned} \end{aligned}$$

Let us study the inner integral above. Note that

$$\begin{aligned} \begin{aligned}&\int _{B(0,\rho )} |(X_jK_0)(z^{-1}yh)-(X_jK_0)(z^{-1}y)|d\mathscr {L}^n(y)\\&\quad \le \underbrace{\int _{\Vert z^{-1}y\Vert \le 2\Vert h\Vert } |(X_jK_0)(z^{-1}yh)-(X_jK_0)(z^{-1}y)|d\mathscr {L}^n(y)}_{\text {(I)}}\\&\qquad +\underbrace{\int _{2\Vert h\Vert \le \Vert z^{-1}y\Vert ,\, \Vert y\Vert \le \rho } |(X_jK_0)(z^{-1}yh)-(X_jK_0)(z^{-1}y)|d\mathscr {L}^n(y)}_{\text {(II)}} \end{aligned} \end{aligned}$$

In order to estimate (I) it suffices to recall that $X_jK_0$ is $(1-Q)$-homogeneous

84 $$\begin{aligned} |\mathrm {(I)}|\le &   \int _{\Vert z^{-1}y\Vert \le 2\Vert h\Vert } |(X_jK_0)(z^{-1}y)|d\mathscr {L}^n(y)\nonumber \\  &   +\int _{\Vert z^{-1}yh\Vert \le 3\Vert h\Vert } |(X_jK_0)(z^{-1}yh)|d\mathscr {L}^n(y) \le 20\sup _{\Vert p\Vert =1}|X_jK_0(p)|\,\Vert h\Vert , \end{aligned}$$

Let us estimate (II). Thanks to [23, Proposition 1.15], there exists a constant $C=C_j>0$ such that $|X_jK_0(a)-X_jK_0(a*h)|\le C\Vert a\Vert ^{-Q}\Vert h\Vert$ whenever $\Vert h\Vert \le \Vert a\Vert /2$, where we recall that *Q* is the homogeneous dimension of $\mathbb {G}$. This implies, in particular, that

85 $$\begin{aligned} \begin{aligned} |\mathrm {(II)}|&\le C\Vert h\Vert \int _{2\Vert h\Vert \le \Vert z^{-1}y\Vert ,\, \Vert y\Vert \le \rho }\Vert z^{-1}y\Vert ^{-Q} d\mathscr {L}^n(y)\\&\le C\Vert h\Vert \int _{2\Vert h\Vert \le \Vert z^{-1}y\Vert \le \rho +\Vert z\Vert }\Vert z^{-1}y\Vert ^{-Q} d\mathscr {L}^n(y). \end{aligned} \end{aligned}$$

Note now that we must impose $\Vert z\Vert \le \rho +2\Vert h\Vert$, otherwise the domain of integration of the second term of the above inequality chain would be empty and hence $|\mathrm {(II)}|=0$. This in turn implies that

86 $$\begin{aligned} \begin{aligned} |\mathrm {(II)}|\le {\tilde{C}}\Vert h\Vert \log \Big (\frac{\rho +\Vert h\Vert }{\Vert h\Vert }\Big ), \end{aligned} \end{aligned}$$

where ${\tilde{C}}$ depends on *C* and *Q*. Summing up all the information gathered above in (82), (83), (84) ,(86), we have discovered that there exists a constant $C_1$ depending on $\mathbb {G}$ and on $K_0$ such that

$$\begin{aligned} \Vert F_2(u)(\cdot *h)- F_2(u)(\cdot )\Vert _{L^1(B(0,\rho ))}\le C_1\Vert u\Vert _{L^1(B(0,\rho ))}\Vert h\Vert \Big (1+ \log \Big (\frac{\rho +\Vert h\Vert }{\Vert h\Vert }\Big )\Big ). \end{aligned}$$

Finally, specializing the above inequality to our case, we infer

$$\begin{aligned} \Vert g_k(\cdot *h)- g_k(\cdot )\Vert _{L^1(B(0,\rho ))}= &   \Vert F_2(R_k)(\cdot *h)- F_2(R_k)(\cdot )\Vert _{L^1(B(0,\rho ))}\\  &   \overset{(81)}{\le } C_1M\Vert h\Vert \Big (1+ \log \Big (\frac{\rho +\Vert h\Vert }{\Vert h\Vert }\Big )\Big ), \end{aligned}$$

proving the equi-continuity of the sequence $\{g_k\}$.

**Step 3.** Thanks to the Step 1 and Step 2 completed above, we can apply Kolmogorov-Riesz-Frechet theorem, see [28, Corollary 8], to infer that $\{g_k\}$ is precompact in $L^1(B(0,1))$.

(iii) strong convergence of $\{f_k\}$ to 0. In this paragraph we aim to prove that the sequence $f_k=-\nabla ^T_\mathbb {G}P_0[\mathfrak {B}[\chi V_k]]*K_0$ defined in (78) converges to 0 in $L^1_{loc}(\mathbb {G})$. The proof follows the following path. First we show that the $f_k$s converges to 0 as distributions, secondly in the weak $L^1$ space $L^{1,\infty }$ and finally in $L^1(B(0,1))$.

**Step 1. Convergence to 0 as distributions.** We can rewrite $f_k$ as

$$\begin{aligned} \langle f_k,\varphi \rangle =\langle \mathcal {L}_1[\chi V_k]*K_0,\varphi \rangle =\langle \chi V_k,\mathcal {L}^*_1[\varphi *K_0]\rangle , \end{aligned}$$

where $\mathcal {L}^*_1$ is the adjoint operator of $\mathcal {L}^1$. Since $\chi V_k$ converge to 0 in $L^1(\mathbb {G})$, it is immediately appart that

$$\begin{aligned} \lim _{k\rightarrow \infty }\langle f_k,\varphi \rangle =\lim _{k\rightarrow 0}\langle \chi V_k,\mathcal {L}^*_1[\varphi *K_0]\rangle =0 \qquad \text {for every test function }\varphi . \end{aligned}$$

Therefore, by definition of weak* convergence of distributions we conclude that $f_k\overset{*}{\rightharpoonup }0$.

**Step 2. Convergence to 0 weakly.** We can rewrite the action of $f_k$ on test functions as

$$\begin{aligned} \begin{aligned} \langle \mathcal {L}_1[\chi V_k]*K_0,\varphi \rangle&=\langle \mathcal {L}_1[\chi V_k],\varphi *K_0^\vee \rangle =\langle -\nabla ^T_\mathbb {G}P_0\mathfrak {B}(\chi V_k),\varphi *K_0\rangle \\&=\sum _{i,j=1}^{n_1}\kappa _j\langle \chi V_k^j*(X_iX_jK_0), \varphi \rangle , \end{aligned} \end{aligned}$$

where $\chi V_k^j$ denotes the *j*th entry of the vector valued function $\chi V_k$. It is easily checked that the distribution $X_iX_j K_0$ is $-Q$ homogeneous and it coincides with a smooth function away from 0. In the notations of [23, p.164], the distribution $X_iX_j K_0$ is said to be a *Kernel of type* 0 and by [23, Proposition 1.8] there is a constant $C>0$ such that

$$\begin{aligned} X_iX_jK_0=C\delta _0+PV(X_iX_jK_0), \end{aligned}$$

where the distribution $PV(X_iX_jK_0)$ acts on test functions $\varphi$ as

$$\begin{aligned} \langle PV(X_iX_jK_0),\varphi \rangle =\lim _{\varepsilon \rightarrow 0}\int _{\Vert x\Vert \ge \varepsilon } X_iX_jK_0(x)\varphi (x) dx. \end{aligned}$$

In order to see that such distribution is well defined we refer to [23, p.166]. In addition, [23, Proposition 1.9] tells us that the operator $T_{i,j}:u\mapsto u*X_iX_jK_0$ is bounded in $L^p(\mathbb {G})$ for every $1<p<\infty$ and thus the operator $F_1$ that acts as

$$\begin{aligned} F_1(u):=\sum _{i,j=1}^{n_1}\kappa _j T_{i,j}(u^j), \qquad \text {where }u\in C^\infty _c(\mathbb {G},\mathbb {R}^{n_1}), \end{aligned}$$

and $u^j$ denotes the *j*th component of *u*, extends to a bounded in $L^p(\mathbb {G},\mathbb {R}^{n_1})$ for every $1<p<\infty$.

For the sake of readability of the notation in the following we will let $T=T_{i,j}$. To be precise [23, Proposition 1.9] gives us a little more. Indeed, defined $T_\varepsilon [u]=u*(X_iX_jK_0)^\varepsilon +Cu$ where $(X_iX_jK_0)^\varepsilon$ is the function coinciding with $X_iX_jK_0$ on $B(0,\varepsilon )^c$ and 0 otherwise, we have that $T_\varepsilon$ are *uniformly* bounded in $L^p$ for every $1<p<\infty$ and

87 $$\begin{aligned} \lim _{\varepsilon \rightarrow 0}\Vert T_\varepsilon [u]-T[u]\Vert _{L^p(\mathbb {G})}=0\qquad \text {for every test function }u. \end{aligned}$$

Let us now show that the operator $u\mapsto u*X_iX_j K_0$ is of weak (1, 1)-type. The above discussion shows that

$$\begin{aligned} T[u]-Cu=\lim _{\varepsilon \rightarrow 0} T_\varepsilon [u]-Cu=\lim _{\varepsilon \rightarrow 0}\int _{\Vert w\Vert \ge \varepsilon }u(\cdot *w^{-1})X_iX_jK_0(w)d\mathscr {L}^n(w), \end{aligned}$$

where the limits above have to been understood in the $L^p$ sense. This in particular implies that the operator

$$\begin{aligned} u\mapsto \lim _{\varepsilon \rightarrow 0}\int _{\Vert w\Vert \ge \varepsilon }u(\cdot *w^{-1})X_iX_jK_0(w)d\mathscr {L}^n(w), \end{aligned}$$

defines an operator bounded on $L^p$. In addition, by [23, Proposition 1.15], there exists a constant $C>0$ such that

$$\begin{aligned}  &   |X_iX_j K_0(w^{-1}z)- X_iX_j K_0(w^{-1}{\bar{z}})|\le C\Vert w^{-1} z\Vert ^{-(Q+1)}\Vert z^{-1}{\bar{z}}\Vert \\  &   \quad \text {for every }\Vert z^{-1}{\bar{z}}\Vert \le \Vert w^{-1}{\bar{z}}\Vert /2. \end{aligned}$$

This implies that there exists a constant $A>0$ such that for every $\varepsilon >0$ we have

$$\begin{aligned} \int _{\Vert w^{-1}z\Vert \ge \varepsilon }|X_iX_j K_0(w^{-1} z)- X_iX_j K_0(w^{-1} {\bar{z}})|\le A\qquad \text {for every } \Vert z^{-1}{\bar{z}}\Vert \le \varepsilon /4, \end{aligned}$$

which, thanks to the fact that the topologies induced by the Euclidean metric and the sub-Riemannian one are the same together with the argument employed in the proof of [52, Chapter 1, §5 Theorem 3], allows us to conclude that the operator $T[u]-Cu$ is of weak (1, 1)-type. Thus, *T*[*u*] is of weak (1, 1)-type as well and so is $F_1$, that is a linear combination of linear operators of weak (1, 1)-type. This together with the fact that $\chi V_k$ converge to 0 in $L^1(\mathbb {G})$, see (79) and (80), implies that

88 $$\begin{aligned} \lim _{k\rightarrow \infty }\Vert f_k\Vert _{L^{1,\infty }(\mathbb {G})}=\lim _{k\rightarrow \infty }\Vert F_1(\chi V_k)\Vert _{L^{1,\infty }(\mathbb {G},\mathbb {R}^{n_1})}=0. \end{aligned}$$

**Step 3: strong convergence to** 0. We now promote the weak convergence to 0 in $L^{1,\infty }$ to a strong convergence to 0 in $L^{1}(B(0,1))$. Thanks to (78) we know that $f_k+g_k\ge 0$ and in particular $f_k^{-}:=\max \{0,-f_k\}\le |g_k|$. However, since $g_k$ is precompact in $L^1(B(0,1))$, the functions $|g_k|$ are locally uniformly integrable, namely for every $\varepsilon >0$ there exists a $\delta _0(\varepsilon )>0$ such that for every Borel set $E\subseteq B(0,1)$ such that $\mathscr {L}^n(E)<\delta _0(\varepsilon )$ we have $\int _E f_k^-\le \int _E|g_k|<\varepsilon$. In addition, since $f_k$ converges to 0 in $L^{1,\infty }(\mathbb {G})$, we have in particular that for every $\eta >0$ we have

89 $$\begin{aligned} \lim _{k\rightarrow \infty }\mathscr {L}^n(\{|f_k|> \eta \})=0. \end{aligned}$$

Let $\eta >0$ and pick a test function $\varphi$ supported on *B*(0, 1). Then

$$\begin{aligned} \begin{aligned} \lim _{k\rightarrow \infty } \int \varphi |f_k|d\mathscr {L}^n=&\lim _{k\rightarrow \infty }\int \varphi f_k d\mathscr {L}^n+2\int \varphi f_k^- d\mathscr {L}^n\\ =&\lim _{k\rightarrow \infty }\langle \chi V_k,\mathcal {L}_1^*(\varphi *K_0)\rangle +2\int \varphi f_k^- d\mathscr {L}^n \\ \le&\lim _{k\rightarrow \infty }\langle \sqrt{\chi } V_k,\sqrt{\chi }\mathcal {L}^*_1(\varphi *K_0)\rangle \\&+2\int _{\{|f_k|>\eta \}} |\varphi |f_k^-d\mathscr {L}^n+2\delta \Vert \varphi \Vert _{L^1(\mathbb {G})}\\ \le&\lim _{k\rightarrow \infty }\Vert \sqrt{\chi } V_k\Vert _{L^1(\mathbb {G})} \Vert \sqrt{\chi }\mathcal {L}^*_1(\varphi *K_0)\Vert _{L^\infty (\mathbb {G})}\\&+2\int _{\{|f_k|>\eta \}\cap B(0,1)} |g_k|d\mathscr {L}^n +2\eta \Vert \varphi \Vert _{L^1(\mathbb {G})}, \end{aligned} \end{aligned}$$

where $\mathcal {L}_1^*$ is the adjoint of the operator $\mathcal {L}_1$. Let us estimate the limit of the last line above. First of all, (80) together with the fact that $\chi$ is supported on *B*(0, 3/4) implies that $\chi V_k$ converges to 0 in $L^1(\mathbb {G})$. Secondly, by (89) we know that for every $\varepsilon >0$ and for every $\eta >0$ we have

$$\begin{aligned} \mathscr {L}^n(\{|f_k|> \eta \}\cap B(0,1))<\delta _0(\varepsilon ),\qquad \text {for every }k\text { sufficiently big.} \end{aligned}$$

This discussion implies that

$$\begin{aligned} \lim _{k\rightarrow \infty } \int \varphi |f_k|d\mathscr {L}^n\le 2\varepsilon +2\eta \Vert \varphi \Vert _{L^1(\mathbb {G})}. \end{aligned}$$

The arbitrariness of $\varepsilon$ and of $\eta$ imply that $\lim _{k\rightarrow \infty } \int \varphi |f_k|d\mathscr {L}^n=0$. This show in particular that the sequence $f_k$ is converging to 0 in $L^1(B(0,1))$.

(iv) final contradiction. Since $g_k$ is precompact in $L^1(B(0,1))$, thanks to step (III) above, we know that the sequence $\chi u_k=f_k+g_k$ is also precompact in $L^1(B(0,1))$. However, since $\chi u_k$ has support contained in *B*(0, 3/4), we infer that $\chi u_k$ is precompact in $L^1(\mathbb {G})$. This implies that there exists a $v\in L^1(\mathbb {G})$ supported on *B*(0, 3/4) such that $\chi u_k\rightarrow v$ in $L^1(\mathbb {G})$.

Let us show that $\Phi _{\varepsilon _k}*[\Vert \varvec{\nu }_k\Vert -\Vert \varvec{\nu }_k\Vert ^s]$ converges to 0 in $L^1(B(0,1/2))$. By definition, we have

90 $$\begin{aligned} \begin{aligned} \lim _{k\rightarrow \infty }\Vert u_k-\Phi _{\varepsilon _k}*\Vert \varvec{\nu }_k\Vert ^s\Vert _{L^1(B(0,1/2))}=&\lim _{k\rightarrow \infty }\int _{B(0,1/2)} \Phi _{\varepsilon _k}*[\Vert \varvec{\nu }_k\Vert -\Vert \varvec{\nu }_k\Vert ^s]d\mathscr {L}^n\\ \le&\lim _{k\rightarrow \infty }\Vert \varvec{\nu }_k\Vert ^a(B(0,1))\\ =&\lim _{k\rightarrow \infty }\frac{\Xi ^a(B(x_0,r_k))}{\Xi (B(x_0,r_k))}= 0, \end{aligned} \end{aligned}$$

where the last line follows from the fact that item (iii) holds at $x_0$. This implies in particular that the sequence $\Phi _{\varepsilon _k}*\Vert \varvec{\nu }_k\Vert ^s$ is precompact in $L^1(B(0,1/2))$. In addition, we also have that $\Phi _{\varepsilon _k}*\Vert \varvec{\nu }_k\Vert ^s\overset{*}{\rightharpoonup }\ \nu$, where $\nu$ was introduced in (71). Indeed, for every test function $\varphi$ supported in *B*(0, 1/2) we have91where the last identity comes from the fact that the sequence of functions $\Phi _{\varepsilon _k}*\varphi$ converges uniformly to $\varphi$. The above chain of identities also proves that

$$\begin{aligned} \langle v,\varphi \rangle =\lim _{k\rightarrow \infty }\langle \chi u_k,\varphi \rangle =\lim _{k\rightarrow \infty } \langle \Phi _{\varepsilon _k}*\Vert \varvec{\nu }_k\Vert , \varphi \rangle =\langle \nu ,\varphi \rangle , \end{aligned}$$

which means that on *B*(0, 1/2) the measure $\nu$ is (represented by) the $L^1(B(0,1/2))$ function *v*.

It is now the moment to choose the sequence $\{\varepsilon _k\}_{k\in \mathbb {N}}$ that are the scales of mollification used to define the functions $u_k$ and $V_k$, see (74). Thanks to the lower semicontinuity of the total variation we know that

$$\begin{aligned} |\Vert \varvec{\nu }_k\Vert ^s -\nu |(B(0,1/2))\le \liminf _{\varepsilon \rightarrow 0}|\Phi _\varepsilon *\Vert \varvec{\nu }_k\Vert ^s -\nu |(B(0,1/2)), \end{aligned}$$

this means that for every $k\in \mathbb {N}$ we can choose an $\varepsilon _k$ such that

92 $$\begin{aligned} |\Vert \varvec{\nu }_k\Vert ^s -\nu |(B(0,1/2))\le |\Phi _{\varepsilon _k}*\Vert \varvec{\nu }_k\Vert ^s -\nu |(B(0,1/2)) +k^{-1}. \end{aligned}$$

Let *E* be a Borel set of $\mathbb {G}$ such that $\mathscr {L}^n(E)=0$, $\Xi ^s(\mathbb {G}\setminus E)=0$. Thanks to (iii) and to (ii) we know that if *k* is sufficiently big, we have

93 $$\begin{aligned} \begin{aligned} 1/j_0\le&\frac{\Xi ^s(B(x_0,r_k/2))}{\Xi (B(x_0,r_k))}=\frac{\Xi ^s(B(x_0,r_k/2)\cap E)}{\Xi (B(x_0,r_k))}=\Vert \varvec{\nu }_k \Vert ^s(B(0,1/2)\cap \delta _{1/r_k}(x_0^{-1}E))\\ \le&|\Vert \varvec{\nu }_k \Vert ^s- \nu |(B(0,1/2)\cap \delta _{1/r_k}(x_0^{-1}E))) + \nu (B(0,1/2)\cap \delta _{1/r_k}(x_0^{-1}E))\\ =&|\Vert \varvec{\nu }_k \Vert ^s- \nu |(B(0,1/2)\cap \delta _{1/r_k}(x_0^{-1}E)))\overset{(92)}{\le }|\Phi _{\varepsilon _k}*\Vert \varvec{\nu }_k\Vert ^s -\nu |(B(0,1/2)) +k^{-1} \end{aligned} \end{aligned}$$

Since $|\Phi _{\varepsilon _k}*\Vert \varvec{\nu }_k\Vert ^s -\nu |(B(0,1/2))=\Vert \Phi _{\varepsilon _k}*\Vert \varvec{\nu }_k\Vert ^s-v\Vert _{L^1(B(0,1/2))}$, we see that if *k* is chosen big enough the inequality

$$\begin{aligned} 1/j_0\le \Vert \Phi _{\varepsilon _k}*\Vert \varvec{\nu }_k\Vert ^s-v\Vert _{L^1(B(0,1/2))} +k^{-1}, \end{aligned}$$

cannot be satisfied thanks to the fact that $u_k\rightarrow v$ in $L^1(B(0,1/2))$ and to (90). This shows that the points where (i), (ii), (iii) and (iv) hold together form a $\Xi ^s$-null set.

(v) conclusion. Thanks to Radon-Nikodym decomposition, we can write $\mu$ as $\mu =\mu ^a+\mu ^s$, where $\mu ^a\ll \mathscr {L}^n$ and $\mu ^s\perp \mathscr {L}^n$ and it is elementary to see that $\mu ^s\ll \Xi ^s$ since $\mu \ll \Xi$. Furthermore, since $\eta _i\ll \Xi$ there are $\alpha _i\in L^1(\Xi )$ such that $\eta ^i=\alpha _i\Xi$. Hence, it is easy to see that

$$\begin{aligned} \begin{aligned} \mathfrak {T}(x)&=\frac{d\mathbb {T}(x)}{d\Xi }=\Big (\frac{d(\tau ^1\eta ^1)}{d\Xi }(x),\ldots ,\frac{d(\tau ^{n_1}\eta ^{n_1})}{d\Xi }(x)\Big )\\&=\Big (\frac{d(\tau ^1\alpha _1\Xi )}{d\Xi }(x),\ldots ,\frac{d(\tau ^{n_1}\alpha _{n_1}\Xi )}{d\Xi }(x)\Big )\\&=(\alpha _1(x)\tau ^1(x),\ldots ,\alpha _{n_1}(x)\tau ^{n_1}(x)), \end{aligned} \end{aligned}$$

for $\Xi$-almost every $x\in \mathbb {G}$. It is thus immediate to see that this implies that

$$\begin{aligned} \mathfrak {T}(x)=(\alpha _1(x) e_1,\ldots ,\alpha _{n_1}(x) e_{n_1})\qquad \text {for }\mu \text {-almost every }x\in \mathbb {G}. \end{aligned}$$

We now show that for $\mu$-almost every $x\in G$ the matrix $\mathfrak {T}(x)$ is invertible. Let us recall that $\mu \ll \eta ^i$ for every $i=1,\ldots ,n_1$, see ($*$), and that $\eta ^i=\alpha _i\Xi$. This implies, in particular, that

$$\begin{aligned} \mu (\mathbb {G}\setminus \{|\alpha _i|>0\})=0\qquad \text {for every }i=1,\ldots ,n_1, \end{aligned}$$

and hence $\mu$ is supported on $\cap _{i=1}^{n_1}\{|\alpha _i|>0\}$, which therefore is a set of full $\mu$-measure. However, thanks to the discussion in the paragraphs (I) to (IV) of this proof, we know that

$$\begin{aligned} \Xi ^s(\{x\in \mathbb {G}:\mathfrak {T}(x)= &   \textrm{diag}(\alpha _1(x)e_1,\ldots ,\alpha _{n_1}(x)e_{n_1})\text { and }0<|\alpha _i|<\infty \\  &   \text { for every }i=1,\ldots ,n_1\})=0. \end{aligned}$$

This, however, concludes the proof of the fact that $\mu ^s=0$. $\square$

## Data Availability

Data sharing is not applicable to this article as no datasets were generated or analysed.

## Measurability Results

In this appendix we collect some measurability results that are proved here in order to make Section 3 and 4 more readable.

### Proposition A.1

For every couple of Borel distributions of homogeneous subgroups $V,W:\mathbb {G}\rightarrow \textrm{Gr}_{\textrm{eu}}(\mathbb {G})$, the intersection map $V\cap W(x):= V(x)\cap W(x)$ is Borel, where we recall that $\textrm{Gr}_{\textrm{eu}}(\mathbb {G})$ was introduced in Definition 2.4.

### Proof

Since for every $\mathbb {V}, \mathbb {W}\in \textrm{Gr}(\mathbb {G})$ we have $\mathbb {V}\cap \mathbb {W}=(\mathbb {V}^\perp +\mathbb {W}^\perp )^\perp$, Proposition 6.1 implies that in order to prove the claim it suffices to show the Borelianity of the sum (as vector subspaces of $\mathbb {G}\cong \mathbb {R}^n$) of the maps $V^\perp$ and $W^\perp$. Let $e_1,\ldots ,e_n$ be a basis of $\mathbb {R}^n$, that as recalled above is underlying vector space of $\mathbb {G}$, and define

$$\begin{aligned} \zeta _i(x):=\pi _{V^\perp (x)}[e_i]\qquad \textrm{and} \qquad \zeta _{i+n}(x):=\pi _{W^\perp (x)}[e_i]\qquad \text {for every } i=1,\ldots ,n. \end{aligned}$$

The vector fields $\zeta _1,\ldots ,\zeta _{2n}:\mathbb {G}\rightarrow \mathbb {G}$ are Borel thanks to Proposition 6.1. In addition by construction we know that the vector fields $\{\zeta _1,\ldots , \zeta _n\}$ span $V(x)^\perp$ while the $\{\zeta _{n+1},\ldots ,\zeta _{2n}\}$ span $W(x)^\perp$ for every $x\in \mathbb {G}$. This implies in particular that $\{\zeta _1(x),\ldots ,\zeta _{2n}(x)\}$ span $V^\perp (x)+ W^\perp (x)$ for every $x\in \mathbb {G}$. Now, from the $\zeta _i$s we construct some other vector fields $\omega _1,\ldots ,\omega _{2n}$ that still span $V^\perp +W^\perp$ defined in the following inductive way. As a first step we define

$$\begin{aligned} \omega _1(x):={\left\{ \begin{array}{ll}\zeta _1(x)/|\zeta _1(x)|&  \textrm{if }\,\zeta _1(x)\ne 0\textrm{,}\\ 0&  \mathrm {otherwise.}\end{array}\right. } \end{aligned}$$

Notice that the vector field $\omega _1(x)$ is trivially seen to be Borel. As a second step, suppose that we already defined the vector fields $\omega _1,\ldots ,\omega _{k-1}$. Define $\tilde{\omega }_k(x):= \zeta _k(x)-\sum _{i=1}^{k-1}\langle \zeta _k(x),\omega _i(x)\rangle \omega _i(x)$ and note that $\tilde{\omega }_k$ is a Borel vector field. Finally, we define $\omega _k$ as

$$\begin{aligned} \omega _k(x)={\left\{ \begin{array}{ll}\tilde{\omega }_k(x)/|\tilde{\omega }_k(x)|&  \textrm{if }\,\tilde{\omega }_k(x)\ne 0\textrm{,}\\ 0&  \mathrm {otherwise.}\end{array}\right. } \end{aligned}$$

Notice that $\omega _k$ is a Borel vector field as well. Thanks to its very definition, we see that for every fixed $x\in \mathbb {G}$ there are only $\textrm{dim}(V^\perp (x)+W^\perp (x))$ non-null elements of $\{\omega _1(x),\ldots ,\omega _{2n}(x)\}$ and those that are not null form an orthonormal basis of $V^\perp (x)+W^\perp (x)$. In particular, for every $x\in \mathbb {G}$ the map $\pi _{V(x)^\perp +W^\perp (x)}$ is easily seen to be represented as

$$\begin{aligned} \pi _{V(x)^\perp +W^\perp (x)}=\sum _{i=1}^{2n}\omega _i(x)\otimes \omega _i(x), \end{aligned}$$

which is a Borel matrix field. This concludes the proof by Proposition 6.1. $\square$

### Proposition A.2

Let *K* be a compact subset of $\mathbb {R}$ and $\gamma :K\rightarrow \mathbb {G}$ be a 1-Lipschitz fragment. Then, for every $\varepsilon >0$ there exists $N=N(\varepsilon )\in \mathbb {N}$, finitely many closed intervals $\{I_j\}_{j=1,\ldots , N}$ and a 1-Lipschitz fragment $\tilde{\gamma }\in \mathcal {X}_N$ such that

$$\begin{aligned} \mathscr {L}^1(\textrm{dom}(\gamma )\,\triangle \,\textrm{dom}(\tilde{\gamma }))\le \varepsilon \mathscr {L}^1(\textrm{dom}\tilde{\gamma }). \end{aligned}$$

### Proof

Since *K* is compact, we can find countably many disjoint open intervals $\{(a_j,b_j)\}_{j\in \mathbb {N}}$ such that

$$\begin{aligned} K\cup \bigcup \{(a_j,b_j):j\in \mathbb {N}\}=[\min K,\max K]. \end{aligned}$$

Let $\varepsilon >0$ arbitrary and choose $N=N(\varepsilon )\in \mathbb {N}$ in such a way that $\sum _{j>N}(b_j-a_j)<\varepsilon$. Then

$$\begin{aligned} \left[ {\min K,\max K} \right] \backslash \bigcup \limits _{{j \le N}} {(a_{j} ,b_{j} )} = K \cup \bigcup \limits _{{j > N}} {\left( {a_{j} ,b_{j} } \right) } , \end{aligned}$$

and in particular $K\cup \bigcup _{j>N}(a_j,b_j)$ is a finite union of closed intervals. Denote now by $\eta _j:[0,d(\gamma (a_j),\gamma (b_j))]\rightarrow \mathbb {G}$ a geodesic joining $\gamma (a_j)$ and $\gamma (b_j)$ and note that if we let

94 $$\begin{aligned} \tilde{\gamma }(t):={\left\{ \begin{array}{ll} \gamma (t) &  \text { if }t\in K,\\ \eta _j\Big (\frac{d(\gamma (a_j),\gamma (b_j))(t-a_j)}{b_j-a_j}\Big )&  \text { if }t\in (a_j,b_j), \end{array}\right. } \end{aligned}$$

then $\tilde{\gamma }$ satisfies $\mathscr {L}^1(\textrm{dom}(\gamma )\,\triangle \,\textrm{dom}(\tilde{\gamma }))\le \varepsilon \mathscr {L}^1(\textrm{dom}\tilde{\gamma })$. In order to conclude the proof, we just need to check that $\tilde{\gamma }$ is a 1-Lipschitz fragment. We check here only the most complicated case in which $s_i\in (a_{j_i},b_{j_i})$ for some $j_1\ne j_2$. Assume without loss of generality that $b_{j_1}<a_{j_2}$ and note that

$$\begin{aligned} \begin{aligned} d(\tilde{\gamma }(s_2),\tilde{\gamma }(s_1)) \le&d( \tilde{\gamma }(b_{j_1}),\tilde{\gamma }(s_1))+d( \tilde{\gamma }(b_{j_1}),\tilde{\gamma }(a_{j_2}))+d(\tilde{\gamma }(a_{j_2}),\tilde{\gamma }(s_2))\\ \le&\frac{d(\gamma (a_{j_1}),\gamma (b_{j_1}))}{b_{j_1}-a_{j_1}}(b_{j_1}-s_1)+(a_{j_2}-b_{j_1})\\&+\frac{d(\gamma (a_{j_2}),\gamma (b_{j_2}))}{b_{j_2}-a_{j_2}}(s_2-a_{j_2})\le (s_2-s_1). \end{aligned} \end{aligned}$$

This concludes the proof. $\square$

### Lemma A.3

Suppose *I* is a Borel subset of the real line an $\Gamma :I\rightarrow \mathfrak {F}$ is a Borel map, where $\mathfrak {F}$ was introduced in Definition 4.1. Then the map $\Psi :I\rightarrow \mathscr {M}(\mathbb {R}^n,\mathbb {R}^n)$ defined as $\Psi (t):=\llbracket \Gamma (t)\rrbracket$ and the map $t\mapsto \Vert \Psi (t)\Vert$ are Borel. Moreover

(i) Suppose $t\mapsto \varvec{\mu }_t$ is a family of vector-valued measures satisfying the hypothesis (a) and (b) of Definition 2.11 and such that for almost every $t\in I$ there exists a 1-Lipschitz fragment $\gamma _t:K_t\rightarrow \mathbb {G}$ defined on a compact set $K_t$ of $\mathbb {R}$ such that $\varvec{\mu }_t=\llbracket \gamma _t\rrbracket$. Then, there exists a Borel map $\Phi :I\rightarrow \mathfrak {F}$ such that $\varvec{\mu }_t=\llbracket \Phi (t)\rrbracket$ for almost every $t\in I$;
(ii) if $\mathcal {M}:I\rightarrow \mathscr {M}(\mathbb {R}^n)$ is a family of measures satisfying the hypothesis (a) and (b) of Definition 2.11 and such that for almost every $t\in I$ there exists a bi-Lipschitz fragment $\gamma _t:K_t\rightarrow \mathbb {G}$ such that . Then, there is a Borel map $\daleth :I\rightarrow \mathscr {M}(\mathbb {R}^n,\mathbb {R}^n)$ such that $\daleth (t)=\llbracket \gamma _t\rrbracket$ and $\mathcal {M}(t)=\Vert \daleth (t)\Vert$ for almost every $t\in I$.

### Proof

First of all, let us note that for every $N\in \mathbb {N}$ the set $\mathfrak {F}_N$ of those $\gamma \in \mathfrak {F}$ such that $\textrm{dom}(\gamma )\subseteq [-N,N]$ is closed in $\mathfrak {F}$ and that $\mathfrak {F}=\cup _{N\in \mathbb {N}}\mathfrak {F}_N$. Note that for every $\gamma \in \mathfrak {F}_N$, we have $\mathbb {M}(\llbracket \gamma \rrbracket )\le 2N$, since the elements of $\mathfrak {F}$ are supposed to be 1-Lipschitz. Let us remark that $\mathscr {M}_N(\mathbb {R}^n,\mathbb {R}^n):=\{\varvec{\nu }\in \mathscr {M}(\mathbb {R}^n,\mathbb {R}^n) :\mathbb {M}(\varvec{\nu })\le N\}$ and the space of the 1-currents in $\mathbb {R}^n$ with mass at most *N*, both endowed with the weak* topology, are isomorphic topological spaces and from now on we will identify them.

   In order to prove the proposition, we prove that the maps $\gamma \mapsto \llbracket \gamma \rrbracket$ and $\gamma \mapsto \Vert \llbracket \gamma \rrbracket \Vert$ are Borel and to do so, we need to introduce three maps. Let

(i) $\mathscr {E}:\mathfrak {F}_N\rightarrow \textrm{Lip}_1([-N,N],\mathbb {G})$ be a map that to each *t* assigns an extension to $[-N,N]$ of $\Gamma (t)$;
(ii) $D:[-N,N]\times \textrm{Lip}_1([-N,N],\mathbb {G})\rightarrow \mathbb {R}^n$ be the map defined as
  $$\begin{aligned} D(s,\gamma ):={\left\{ \begin{array}{ll} \gamma '(s) &  \text {if }\gamma '(s)\text { exists,}\\ 4e_1 &  \text {otherwise}. \end{array}\right. } \end{aligned}$$
(iii) $\mathfrak {M}:\mathscr {K}([-N,N])\rightarrow \mathscr {M}([-N,N])$ be the map defined as , where we recall that $\mathscr {K}([-N,N])$ is the family of compact sets of $[-N,N]$ endowed with the Hausdorff distance and $\mathscr {M}([-N,N])$ is endowed with the weak* topology of measures.

We now check that that we can find an extension map $\mathscr {E}$ that is Borel, and that $\mathfrak {M}$ and *D* are Borel.

borelianity of $\mathscr {E}$ Let us prove that we can construct an extension map $\mathscr {E}$ that is Borel. In order to do this, we will construct a multimap that assigns to each element of $\mathfrak {F}_N$ the family of all its extensions. We prove that this multimap is measurable and conclude by Kuratowski-Ryll-Nardzewski selection theorem that it admits a Borel selection that will be our sought map $\mathscr {E}$.

   As a first step, we construct the (graph of the) extension multimap. Let $\mathfrak {d}:\mathfrak {F}\times \textrm{Lip}_1([-N,N],\mathbb {G})\rightarrow \mathbb {R}$ be the map defined as

$$\begin{aligned} \mathfrak {d}(\gamma ,\eta ):=\sup _{z\in \textrm{gr}(\gamma )}\textrm{dist}_{eu}(z,\textrm{gr}(\eta )). \end{aligned}$$

Let us note that if $\mathfrak {d}(\gamma ,\eta )=0$ where $\gamma \in \mathfrak {F}$ and $\eta \in \textrm{Lip}_1([-N,N],\mathbb {G})$, then this implies that $\textrm{gr}(\gamma )\subseteq \textrm{gr}(\eta )$. Therefore, if $(\gamma ,\eta )\in \mathfrak {d}^{-1}(0)$, then $\gamma \in \mathfrak {F}_N$ and $\eta$ is an extension of the map $\gamma$ to $[-N,N]$. Let us check that $\mathfrak {d}$ is Lipschitz. Indeed

95 $$\begin{aligned} \begin{aligned} |\mathfrak {d}(\gamma _1,\eta _1)-\mathfrak {d}(\gamma _2,\eta _2)|&\le |\mathfrak {d}(\gamma _1,\eta _1)-\mathfrak {d}(\gamma _1,\eta _2)|+|\mathfrak {d}(\gamma _1,\eta _2)-\mathfrak {d}(\gamma _2,\eta _2)|\\&\le d_{eu,\mathscr {H}}(\eta _1,\eta _2)+d_{eu,\mathscr {H}}(\gamma _1,\gamma _2). \end{aligned}\nonumber \\ \end{aligned}$$

Therefore, $\mathfrak {d}^{-1}(0)$ is a closed set in $\mathfrak {F}\times \textrm{Lip}_1([-N,N],\mathbb {G})$. Finally, thanks to Proposition A.2, the projection of $\mathfrak {d}^{-1}(0)$ on $\mathfrak {F}$ coincides with $\mathfrak {F}_N$. This implies that that the multimap that assigns to each $\gamma \in \mathfrak {F}_N$ the family of all its extensions in $\textrm{Lip}_1([-N,N],\mathbb {G})$ is closed, i.e. it takes closed sets as values, as its (multi)graph coincides with $\mathfrak {d}^{-1}(0)$. If the extension multimap is shown to be Borel measurable, the Borelianity of $\mathscr {E}$ is proven thanks to Kuratowski-Ryll-Nardzewski selection theorem, see [51, Theorem 5.2.1].

   Now we check that the extension multimap is Borel. To do this, it is sufficient to prove that for every closed set $C\subseteq \textrm{Lip}_1([-N,N],\mathbb {G})$ the family of those $\gamma \in \mathfrak {F}_N$ that are extended by some $\eta \in C$ is Borel, see [51, p. 184]. Without loss of generality we can assume that *C* is compact. Indeed, we can write $\textrm{Lip}_1([-N,N],\mathbb {G})=\cup _{R\in \mathbb {N}}\textrm{Lip}_1([-N,N],B(0,R))$ and note that each $\textrm{Lip}_1([-N,N],B(0,R))$ is compact. This implies in particular that $C_R:=C\cap \textrm{Lip}_1([-N,N],B(0,R))$ is compact. Therefore, if we show that for every compact set $C\subseteq \textrm{Lip}_1([-N,N],\mathbb {G})$ the family of those $\gamma \in \mathfrak {F}_N$ that are extended by some $\eta \in C$ is Borel, the proof of the claim is achieved. However, if we take a sequence $\{\gamma _k\}_{k\in \mathbb {N}}\in \mathfrak {F}_N$ with the property that they are extended by some curve $\{\bar{\gamma }_k\}_{k\in \mathbb {N}}$ in the compact set *C*, up to subsequences there exist $\gamma \in \mathfrak {F}_N$ and $\bar{\gamma }\in C$ such that $\lim _{k\rightarrow \infty }d_{eu,\mathscr {H}}(\gamma _k,\gamma )=0$ and $\lim _{k\rightarrow \infty }d_{eu,\mathscr {H}}(\bar{\gamma }_k,\bar{\gamma })=0$. It is an elementary computation to see that $\bar{\gamma }$ is an extension of $\gamma$. This shows that the set of those $\gamma \in \mathfrak {F}_N$ that are extended by some $\eta \in C$ is compact. Therefore, this concludes the proof that the extension multimap is Borel and hence such multimap admits a Borel selection that we denote by $\mathscr {E}$.

   Let us further check that the map $(\gamma ,s)\mapsto \mathscr {E}(\gamma )(s)$ is Borel. First note that evaluation map $\mathbb {E}_s:\textrm{Lip}([-N,N],\mathbb {R}^n)\rightarrow \mathbb {R}^n$ defined as $\mathbb {E}_s(\gamma ):=\gamma (s)$ is continuous on $\textrm{Lip}([-N,N],\mathbb {R}^n)$. Therefore the map $s\mapsto \mathscr {E}(\gamma )(s)$ is continuous for every $\gamma$ and $\gamma \mapsto \mathscr {E}(\gamma )(s)=\mathbb {E}_s(\mathscr {E}(\gamma ))$ is Borel for every $s\in [-N,N]$. The check of the joint Borelianity is elementary. It suffices to observe that the above remarks imply that the Borel maps

$$\begin{aligned} \mathscr {E}_k(\gamma )(s):=\mathscr {E}(\gamma )(\lfloor ks\rfloor /k) \end{aligned}$$

converge pointwise to $\mathscr {E}(\gamma )(s)$.

borelianity of *D* Fix a closed set $C\subseteq \mathbb {R}^n$ and $\varepsilon ,\delta >0$. Let us note that the set $\mathscr {D}_{\varepsilon ,\delta ,C}$ of those $(s,\gamma )\in [-N,N]\times \textrm{Lip}_1([-N,N],\mathbb {G})$ for which there exists a $v\in C$ such that

96 $$\begin{aligned} |\gamma (\sigma )- \gamma (s)-(\sigma -s)v|\le \varepsilon |\sigma -s|,\qquad \text {for every }|\sigma -s|\le \delta \end{aligned}$$

is closed. Let $(s_i,\gamma _i)\in \mathscr {D}_{\varepsilon ,\delta ,C}$ satisfy (96) for some $v_i\in C$ and suppose $(s_i,\gamma _i)$ converge to $(s,\gamma )\in [-N,N]\times \textrm{Lip}_1([-N,N],\mathbb {G})$. Since the $\gamma _i$s are converging to $\gamma$ in $d_{eu,\mathscr {H}}$, we infer that there there exists a constant $\mathfrak {c}=\mathfrak {c}(\varepsilon ,\gamma )$ such that $\Vert v_i\Vert \le \mathfrak {c}$ and thus, up to subsequences, $v_i\rightarrow v$ for some $v \in C$. This together with an elementary computation shows also that $\mathscr {D}_{\varepsilon ,\delta ,C}$ is closed. Therefore, we infer that

$$\begin{aligned} \mathscr {D}_C:=\bigcap _{\varepsilon \in \mathbb {Q}^+}\bigcup _{\delta \in \mathbb {Q}^+}\mathscr {D}_{\varepsilon ,\delta ,C} \end{aligned}$$

is Borel. The set of those $(s,\gamma )$ where $\gamma '(s)$ does not exists is contained in $\mathscr {D}_{\mathbb {R}^n}^c$, which is clearly Borel.

   Let us check the Borelianity of *D*. First of all, let us observe since $\gamma \in \textrm{Lip}_1([-N,N],\mathbb {G})$ we have that whenever $\gamma '$ exists, we have $|\pi _1(\gamma ')|\le 1$ by the definition of the group operation. Therefore the image under *D* of those points $(s,\gamma )$ such that $\gamma$ is differentiable at *s* is contained in the cylinder $\kappa :=\{z\in \mathbb {R}^n:|\pi _1(z)|\le 1\}$. Therefore, the Borelianity of *D* follows by observing that if *C* is a closed set not intersecting $\{4e_1\}$, we have ${D}^{-1}(C)=\mathscr {D}_C$, which is a Borel set as shown above. If on the other hand $4e_1\in C$, we have

$$\begin{aligned} {D}^{-1}(C)={D}^{-1}(C\cap \kappa \cup \{4e_1\})={D}^{-1}(C\cap \kappa )\cup {D}^{-1}(\{4e_1\})=\mathscr {D}_{C\cap \kappa }\cup \mathscr {D}_{\{4e_1\}}, \end{aligned}$$

which is Borel.

borelianity of $\mathfrak {M}$ Let us check that the map $\mathfrak {M}$ is Borel. Recall that the sets

$$\begin{aligned} B(\psi ;\phi ,\varepsilon ):=\Big \{\mu \in \mathscr {M}([-N,N]):\Big |\int \phi \, d\mu -\int \phi \, d\psi \Big |< \varepsilon \Big \}, \end{aligned}$$

where $\psi \in \mathscr {M}([-N,N])$, $\phi \in C_c([-N,N])$ and $\varepsilon >0$, are a pre-basis of the weak* topology of $\mathscr {M}([-N,N])$. Therefore, in order to check the Borelianity of $\mathfrak {M}$ it is sufficient to prove that the sets $U(\psi ;\phi ,\varepsilon ):=\mathfrak {M}^{-1}(B(\psi ;\phi ,\varepsilon ))$ are Borel for every $\psi$, $\phi$ and $\varepsilon$ as above. Thanks to the following chain of identities

97

in order to prove the Borelianity of $U(\psi ;\phi ,\varepsilon )$ it is therefore sufficient to show that the maps

are upper semi-continuous, where as usual $\phi ^+$ and $\phi ^-$ are the positive and negative parts of $\phi$.

   Let us prove that $f_+$ is upper semi-continuous and the result for $f_-$ will follow similarly. Let $\eta >0$ and $\{K_\iota \}_{\iota \in \mathbb {N}}$ be a sequence of compact sets converging in the Hausdorff distance to some compact set *K*. Finally, fix an open set *A* containing *K* such that

$$\begin{aligned} \int _A \phi ^+ d\mathscr {L}^1 \le \int _K \phi ^+ d\mathscr {L}^1+\eta . \end{aligned}$$

Such open set *A* exists thanks to the continuity of $\phi ^+$ and the Borel regularity of the Lebesgue measure. On the other hand, it is immediately seen that the compact sets $K_\iota$, that converge to *K* in the Hausdorff distance, are contained in *A* for $\iota$ sufficiently big. Therefore

$$\begin{aligned} \limsup _{\iota \rightarrow \infty } f_+(K_\iota )\le \int _A \phi ^+d\mathscr {L}\le f_+(K)+\eta . \end{aligned}$$

The arbitrariness of $\eta$ concludes the proof of the fact that $U(\psi ;\phi ,\varepsilon )$ is Borel for every $\psi \in \mathscr {M}([-N,N])$, $\phi \in C_c(\mathbb {R})$ and every $\varepsilon >0$. This concludes the proof of the Borelianity of $\mathfrak {M}$.

construction and borelianity of $\Psi$ Let $\Psi _N:\mathfrak {F}_N\rightarrow \mathscr {M}(\mathbb {R}^n,\mathbb {R}^n)$ be the map defined as follows. For every $\gamma \in \mathfrak {F}_N$ and $\phi \in C^\infty _c(\mathbb {R}^n,\mathbb {R}^n)$ we let $\Psi _N(t)\in \mathscr {M}(\mathbb {R}^n,\mathbb {R}^n)$ be such that

$$\begin{aligned} \langle \Psi _N(\gamma )\, ; \, \phi \rangle :=\int \langle D(s,\mathscr {E}(\gamma ))\, ; \, \phi (\mathscr {E}(\gamma )(s))\rangle d\nu _\gamma (s)=:f_\phi (\gamma ), \end{aligned}$$

where $\nu _\gamma :=\mathfrak {M}(\textrm{dom}(\gamma ))$. Throughout the rest of this paragraph, in order to improve readability with abuse of notations, we will let $\Psi :=\Psi _N$ as *N* will be fixed. In the following we aim to prove that $\Psi$ is Borel. In order to do this, it is easy to check, in a similar way to what we did in the proof of the Borelianity of $\mathfrak {M}$, that it suffices to show that for every $\phi \in C_c(\mathbb {R}^n,\mathbb {R}^n)$ and every $r>0$ the set

$$\begin{aligned} \{\gamma \in \mathfrak {F}_N: \langle \Psi (\gamma )\, ; \, \phi \rangle \le r\}=\{\gamma \in \mathfrak {F}_N: f_\phi (\gamma )\le r\},\qquad \text {is Borel.} \end{aligned}$$

Hence, the Borelianity of the map $\Psi$ is equivalent to that of all the maps $f_\phi$. Therefore, here below we check that the maps $f_\phi$ are Borel.

   As a first step, let us check that the map $\gamma \mapsto \nu _\gamma$ is Borel. The map $\gamma \mapsto \textrm{dom}(\gamma )$ is Borel, and this can be checked by recalling that $\Gamma$ is Borel and that the projection of the graphs $\gamma$ on the first component is a 1-Lipschitz map with respect to the Hausdorff distance. Further, since $\mathfrak {M}$ was shown in the previous paragraph to be Borel, we infer that $\gamma \mapsto \nu _\gamma =\mathfrak {M}(\textrm{dom}(\gamma ))$ is a Borel map.

   Clearly, in order to prove that $f_\phi$ is Borel we now proceed in steps. Let us fix notations. For every Borel function $g:\mathfrak {F}_N\times [-N,N]\rightarrow \mathbb {R}$ bounded with bounded support we define the map

$$\begin{aligned} \Xi _g:\gamma \mapsto \int g(\gamma ,s)\,d\nu _\gamma (s). \end{aligned}$$

   First we note that for every Borel sets $E_1\subseteq \mathfrak {F}_N$ and $E_2\subseteq [-N,N]$ the map $\Xi _{\mathbb {1}_{E_1}\mathbb {1}_{E_2}}$ is Borel. This however is an immediate consequence of the fact that the map $\gamma \mapsto \nu _\gamma$ is Borel. Indeed, it can be checked that this implies that for every Borel set $E\subseteq \mathbb {R}$ we have that the map $\gamma \mapsto \nu _\gamma (E)$ is Borel. Compare with Definition 2.11.

   Now we prove that for every continuous function *g* with bounded support we have that the map $\Xi _g$ is Borel. Let us note here that bounded and closed subsets of $\mathfrak {F}_N$ are compact. First recall that *g* is uniformly continuous (having compact support) and therefore for every $\varepsilon >0$ there exists $\delta >0$ such that if $d_{eu,\mathscr {H}}(\gamma _1,\gamma _2)\le \delta$ and $|s_1-s_2|\le \delta$ then

$$\begin{aligned} |g(\gamma _1,s_1)-g(\gamma _2,s_2)|\le \varepsilon . \end{aligned}$$

Let $\{A_j^\delta \}_{j\in \mathbb {N}}$ be a Borel (countable) partition of $\mathfrak {F}_N$ such that $\textrm{diam}(A_j)\le \delta$ and let $\gamma _j^\delta \in A_j^\delta$. Then, we let $L\ge -\log _2(\delta /N)$ and

$$\begin{aligned} g_\delta (\gamma ,s):= &   \sum _{j\in \mathbb {N}}\sum _{k=-2^{L}}^{2^L-1}g(\gamma _j^\delta ,Nk2^{-L})\mathbb {1}_{A_j^\delta }(\gamma )\mathbb {1}_{[Nk2^{-L},N(k+1)2^{-L})}(s)\\  &   +\sum _{j\in \mathbb {N}}g(\gamma _j^\delta ,N)\mathbb {1}_{A_j^\delta }(\gamma )\mathbb {1}_{\{N\}}(s). \end{aligned}$$

It is easy to see that $g_\delta$ has bounded support, since *g* has compact support, and that $g_\delta$ converges to *g* uniformly. On the one hand, it is follows from the discussion above that the functions $\Xi _{g_\delta }$ are Borel, as they are sum of Borel functions. However, it is also immediate to see that $\Xi _{g_\delta }$ converge uniformly to $\Xi _g$ on $\mathfrak {F}_N$ and hence $\Xi _g$ is Borel as it can be written as a limit of a sequence of Borel functions.

   However, this actually concludes the proof. Let us see why this is the case. We recall that a function is of Baire class $\zeta \in \mathbb {N}$ if it is the pointwise limit of a sequence of functions in the Baire class $\zeta -1$. The Baire class 0 is by definition the family of continuous functions.

   Let us prove that if *g* is bounded, boundedly supported and of Baire class 1, then $\Xi _g$ is Borel. By definition, we can find a sequence of continuous functions $g_\ell$ converging to *g* pointwise. It is immediate to see that we can assume that the functions $g_\ell$ are uniformly bounded and that their support is contained in a common bounded set. However, for every $\gamma \in \mathfrak {F}_N$ this implies that $\lim _{\ell \rightarrow \infty }\Xi _{g_\ell }(\gamma )=\Xi _g(\gamma )$ by dominated convergence theorem. This shows, since the functions $\Xi _{g_\ell }$ are Borel, that $\Xi _g$ is Borel as well.

   However, since every Borel function is in some Baire class, see [31, Theorem 24.3], arguing as above inductively, implies that $\Xi _g$ is Borel whenever *g* is bounded with bounded support.

   Let us conclude this paragraph with the proof of the Borelianity of $\Psi$. The function

$$\begin{aligned} g_\phi (\gamma ,s):=\langle D(s,\mathscr {E}(\gamma ))\, ; \, \phi (\mathscr {E}(\gamma )(s))\rangle , \end{aligned}$$

is Borel since all the functions involved in the definition are Borel, and it is easy to check that it has bounded support and it is bounded. Indeed $D(\gamma ,s)\le 1$ for every $(\gamma ,s)\in \mathfrak {F}_N\times [-N,N]$, since the curves $\gamma$ are 1-Lipschitz, and hence $\Vert g_\phi \Vert _\infty \le \Vert \phi \Vert _\infty$. This proves that the functions $\Xi _{g_\phi }=f_\phi$ are Borel for every $\phi \in C_c(\mathbb {R}^n,\mathbb {R}^n)$. This concludes the proof that $\Psi$ is Borel.

   We further observe that for every $\gamma \in \mathfrak {F}_N$ and $\phi \in C_c(\mathbb {R}^n)$ we have that

$$\begin{aligned} \langle \Vert \Psi (\gamma )\Vert \, ; \, \phi \rangle =\int \phi (\mathscr {E}(\gamma )(s))d\nu _\gamma (s). \end{aligned}$$

Hence, the above argument proves also that $\Vert \Psi (\gamma )\Vert$ is Borel.

conclusion of the proof. Since $\Gamma$ is Borel, we infer that $\mathscr {T}_N:=\Gamma ^{-1}(\mathfrak {F}_N)\cap [-N,N]$ is Borel, where $\Gamma$ is the curve of curves given in the statement of the lemma. Therefore, the Borelianity of $t\mapsto \llbracket \Gamma (t)\rrbracket$ and that of $t\mapsto \Vert \llbracket \Gamma (t)\rrbracket \Vert$ follow from the fact that $\llbracket \Gamma (t)\rrbracket =\Psi _N(\Gamma (t))$ for every $t\in \mathscr {T}_N$.

   Towards a proof of item (i) of the Lemma, consider the set $\mathscr {X}$ the set of those $(t,\gamma )\in I\times \mathfrak {F}$ such that $\varvec{\mu }_t=\llbracket \gamma \rrbracket$. By assumption, there exists a Borel subset ${\tilde{I}}\subset I$ of full measure, for which for every $t\in {\tilde{I}}$ there exists $\gamma \in \mathfrak {F}$ such that $\varvec{\mu }_t=\llbracket \gamma \rrbracket$ and such that the map $t\mapsto \varvec{\mu }_t$ restricted to ${\tilde{I}}$ is Borel. This is due to Lusin’s theorem. Define now the map $\gimel _1:(t,\gamma )\mapsto \mathbb {M}[\varvec{\mu }_t-\llbracket \gamma \rrbracket ]$ and let us check that it is Borel. First of all, the mass $\mathbb {M}$ is lower semicontinuous and the map ${\tilde{I}}\times \mathfrak {F}\ni (t,\gamma )\mapsto \varvec{\mu }_t-\llbracket \gamma \rrbracket$ is Borel. Therefore, $\mathscr {X}=\Xi ^{-1}(0)$ is Borel and von Neumann measurable selection theorem, see [51, Theorem 5.5.2] guarantees that there exists a universally measurable selection $\tilde{\Phi }:\tilde{I}\rightarrow \mathfrak {F}$ of $\mathscr {X}$. Therefore, there exists a Borel map $\Phi :I\rightarrow \mathfrak {F}$ that coincides $\mathscr {L}^1$-almost everywhere on *I* with $\tilde{\Phi }$ and hence

$$\begin{aligned} \varvec{\mu }_t=\llbracket \Phi (t)\rrbracket \qquad \text {for } \mathscr {L}^1\text {-almost every }t\in I. \end{aligned}$$

   Finally, the proof of item (ii) can be obtained with the same argument, mutatis mutandis, employed for item (i) by showing that with von Neumann selection theorem, we can extract a universally measurable section of the set $\mathscr {Y}$ of those $(t,\gamma )\in I\times \mathfrak {F}$ such that $\mu _t=\Vert \llbracket \gamma \rrbracket \Vert$. The Borelianity of the set $\mathscr {Y}$ is inferred from the Borelianity of the map $\gimel _2(t,\gamma ):=\mathbb {M}[\varvec{\mu }_t-\Vert \llbracket \gamma \rrbracket \Vert ]$, since $\mathscr {Y}=\gimel _2^{-1}(0)$. $\square$

### Proposition A.4

Let (*I*, *dt*) be a (possibly unbounded) Borel subset of real line endowed with the Lebesgue measure $\mathscr {L}^1$ and $t\mapsto \varvec{\mu }_t$ be a family of vector-valued measures satisfying the hypothesis (a) and (b) of Definition 2.11 and such that for almost every $t\in I$ there exists a 1-Lipschitz fragment $\gamma _t:K_t\rightarrow \mathbb {G}$ defined on a compact set $K_t$ of $\mathbb {R}$ such that $\varvec{\mu }_t=\llbracket \gamma _t\rrbracket$. Then, for every $\varepsilon >0$ there exists a Borel set $I_\varepsilon \subset I$, $N\in \mathbb {N}$ and a Borel map $\mathfrak {c}_\varepsilon :I_\varepsilon \rightarrow \mathscr {M}(\mathbb {R}^n,\mathbb {R}^n)$ such that

(i) for every $t\in I_\varepsilon$ we have $\mathfrak {c}_\varepsilon (t)=\llbracket \Gamma (t)\rrbracket$ where the map $\Gamma :I_\varepsilon \rightarrow \mathcal {X}_N$ is a Borel map with respect to the metric $d_{eu,\mathscr {H}}$ introduced in Definition 4.1;
(ii) $$\int _{I\setminus I_\varepsilon }\mathbb {M}(\varvec{\mu }_s)\,ds<\varepsilon /2$$, $$\int _{I_\varepsilon } \mathbb {M}(\varvec{\mu }_s-\mathfrak {c}_\varepsilon (s))\,ds<\varepsilon /2$$ and $$\Vert \mathbb {M}(\mathfrak {c}_\varepsilon )\Vert _{L^\infty (I_\varepsilon )}<\infty $$.

### Proof

Throughout the proof, we identify without further comment $\mathbb {G}$ with its underlying vector space $\mathbb {R}^n$ as Lipschitz fragments in $\mathbb {G}$, as it has been previously remarked, are Lipschitz in $\mathbb {G}\cong \mathbb {R}^n$ endowed with the Euclidean metric. Without loss of generality, we can assume that the $\varvec{\mu }_t$’s are supported on the closed ball *B*(0, *R*) for some $R>0$. Since $t\mapsto \mathbb {M}(\varvec{\mu }_t)$ is supposed to be measurable and since $\int _\mathbb {R}\mathbb {M}(\varvec{\mu }_t)dt<\infty$, for every $\varepsilon >0$ there exists a compact set $\tilde{I}_\varepsilon$ such that $\int _{I\setminus {{\tilde{I}}}_\varepsilon }\mathbb {M}(\varvec{\mu }_t)dt<\varepsilon /6$ and $\Vert \mathbb {M}(\varvec{\mu }_t)\Vert _{L^\infty ({\tilde{I}}_\varepsilon )}<\infty$ and $t\mapsto \mu _t$ is Borel on $\tilde{I}_\varepsilon$.

Fix $N\in \mathbb {N}$. In the proof of Lemma A.3 we have showed that the map $\Xi :I\times \mathfrak {F}\rightarrow \mathscr {M}(\mathbb {R}^n,\mathbb {R}^n)$ defined as $(t,\gamma )\mapsto \mathbb {M}(\varvec{\mu }_t-\llbracket \gamma \rrbracket )$ is Borel. Let $\mathscr {G}_{\varepsilon ,N}:=\tilde{I}_\varepsilon \times \mathcal {X}_N\cap \Xi ^{-1}([0,\varepsilon /6\mathscr {L}(\tilde{I}_\varepsilon )))$ and note that by the Borelianity of $\Xi$ we infer that $\mathscr {G}_{\varepsilon ,N}$ is Borel and that the set

$$\begin{aligned} \mathscr {I}_{\varepsilon ,N}:=\{t\in {\tilde{I}}_{\varepsilon }:\text { there exists }\gamma \in \mathcal {X}_N\text { such that }(t,\gamma )\in \mathscr {G}_{\varepsilon ,N}\}\qquad \text {is Suslin}. \end{aligned}$$

In particular $\mathscr {I}_{\varepsilon ,N}$ is universally measurable. We define $\mathscr {I}_\varepsilon :=\cup _{i\in \mathbb {N}}\mathscr {I}_{\varepsilon ,i}\setminus \mathscr {I}_{\varepsilon ,i-1}$ and note that thanks to Proposition A.2 we have $\mathscr {L}^1(\tilde{I}_\varepsilon \setminus \mathscr {I}_\varepsilon )=0$ and that $\mathscr {I}_\varepsilon$ is universally measurable. Therefore, there exists an $N\in \mathbb {N}$ and a compact set $I_\varepsilon$ of $\mathscr {I}_{\varepsilon ,N}$ such that

$$\begin{aligned} \int _{{\tilde{I}}_\varepsilon \setminus I_\varepsilon }\mathbb {M}(\varvec{\mu }_t)\,dt\le \varepsilon /6. \end{aligned}$$

Recalling that both $I_\varepsilon$ and $\mathcal {X}_N$ are Polish spaces, von Neumann measurable selection theorem, see [51, Theorem 5.5.2], implies that there exists a universally measurable uniformization of $I_\varepsilon \times \mathcal {X}_N\cap \mathscr {G}_{\varepsilon ,N}$, that is, a universally measurable map $\tilde{\Gamma }_{\varepsilon }:I_\varepsilon \rightarrow \mathcal {X}_N$ such that $(t,\tilde{\Gamma }_{\varepsilon }(t))\in \mathscr {G}_{\varepsilon ,N}$. By Lusin’s theorem, there exists a map that we denote with $\Gamma _{\varepsilon }$ that coincides with the map $\tilde{\Gamma }_\varepsilon$ outside a $\mathscr {L}^1$-null subset of $I_\varepsilon$.

Therefore, applying Lemma A.3 to $\Gamma _\varepsilon$ we get that the map $\mathfrak {c}_\varepsilon :I_\varepsilon \rightarrow \mathscr {M}(\mathbb {R}^n,\mathbb {R}^n)$ defined as $\mathfrak {c}_\varepsilon (t):=\llbracket \Gamma _\varepsilon (t)\rrbracket$ is Borel. Let us now check item (ii). First of all

$$\begin{aligned} \int _{I\setminus I_\varepsilon }\mathbb {M}(\varvec{\mu }_t)dt\le \int _{I\setminus {\tilde{I}}_\varepsilon }\mathbb {M}(\varvec{\mu }_t)dt+\int _{{\tilde{I}}\setminus I_\varepsilon }\mathbb {M}(\varvec{\mu }_t)dt\le 2\varepsilon /6<\varepsilon /2. \end{aligned}$$

Secondly

$$\begin{aligned} \int _{I_\varepsilon } \mathbb {M}(\varvec{\mu }_s-\mathfrak {c}_\varepsilon (s))\,ds\le \mathscr {L}^1(I_\varepsilon )\cdot \frac{\varepsilon }{6\mathscr {L}^1({\tilde{I}}_\varepsilon )}<\varepsilon /2, \end{aligned}$$

Where in the first inequality above we used the fact that by construction

$$\begin{aligned} \mathbb {M}(\varvec{\mu }_t-\mathfrak {c}_\varepsilon (t))=\mathbb {M}(\varvec{\mu }_t-\llbracket \Gamma _\varepsilon (t)\rrbracket )\le \varepsilon /6\mathscr {L}^1({\tilde{I}}_\varepsilon ). \end{aligned}$$

Finally, for almost every $t\in I_\varepsilon$, we have

$$\begin{aligned} \mathbb {M}(\mathfrak {c}_\varepsilon (t))\le \mathbb {M}(\varvec{\mu }_t)+\mathbb {M}(\varvec{\mu }_t-\mathfrak {c}_\varepsilon (t))<\infty . \end{aligned}$$

This concludes the proof. $\square$

### Lemma A.5

Let $n,m\in \mathbb {N}$. For every $r>0$ the map $\textsf{E}_r^{n,m}:\mathscr {M}(\mathbb {R}^n,\mathbb {R}^m)\times \mathbb {R}^n\rightarrow \mathbb {R}^m$ defined as $\textsf{E}_r^{n,m}(\varvec{\nu },x):={\varvec{\nu }}(U(x,r))$ is Borel, where *U*(*x*, *r*) denotes the closed Euclidean ball with centre *x* and radius $r>0$.

### Proof

First of all, let us prove that the map $\textsf{E}_r^+:\mathscr {M}(\mathbb {R}^n)\times \mathbb {R}^n\rightarrow \mathbb {R}$ defined as $\textsf{E}_r^+(U(x,r)):=\nu (U(x,r))$ is Borel. Without loss of generality we can restrict the map $\textsf{E}_r^+$ to a set of measures with equibounded mass. Let $\nu _i\overset{*}{\rightharpoonup }\nu$ and $x_i\rightarrow x$ and note that for every $\varepsilon >0$ we have

98 $$\begin{aligned} \limsup _{i\rightarrow \infty }{\textsf{E}}_r^+(\nu _i,x_i)=\limsup _{i\rightarrow \infty }\nu _i(U(x_i,r))\nonumber \\ \le \limsup _{i\rightarrow \infty }\nu _i(U(x,r+\varepsilon ))\le \nu (U(x,r+\varepsilon )), \end{aligned}$$

where the last inequality follows from Portmanteau’s theorem. However, thanks to the arbitrariness of $\varepsilon$, we conclude that ${\textsf{E}}_r^+$ is upper semi-continuous. This concludes the proof for positive measures.

For measures in $\mathscr {M}(\mathbb {R}^n,\mathbb {R})$, one just uses Hahn’s decomposition theorem and applies the above discussion to the negative and positive part and recalls that the sum of Borel functions is Borel.

Finally, to obtain the result for vector valued case we apply the above discussion component-wise. $\square$

### Lemma A.6

Let *I* be a Borel subset of $\mathbb {R}$. Suppose that $I\ni t\mapsto \mu _t$ is a map satisfying items (a) and (b) of Definition 2.11 and for which for almost every $t\in I$ there exists a Lipschitz fragment $\gamma _t:K_t\rightarrow \mathbb {G}$ such that . Then, there exists a Borel map $\mathfrak {v}:I\times \mathbb {G}\rightarrow V_1$ and a Borel set $E\subseteq I\times \mathbb {G}$ such that for almost every $t\in I$ we have

$$\begin{aligned} \mathscr {H}^1\big ( \textrm{im}(\gamma _t)\triangle \{x\in \mathbb {G}:(t,x)\in E\}\big )=0, \end{aligned}$$

$\mathfrak {v}(t,x)\in \{\lambda D\gamma _t(s)\in V_1\setminus \{0\}:\lambda \in \mathbb {R}\text { and }s\in \gamma _t^{-1}(x)\}$ and $|\mathfrak {v}(x,t)|=1$ for every $(t,x)\in E$ and $\mathfrak {v}=0$ otherwise.

### Proof

We divide the proof in several steps. First, since $t\mapsto \mu _t$ is measurable, by Lusin’s theorem there exists a Borel map coinciding with $t\mapsto \mu _t$ up to Lebesgue null sets. Thus, in the following we will assume that the map $t\mapsto \mu _t$ is Borel and that  for every $t\in I$. This reduction is without loss of generality because we are required to prove a statement up to sets of null $\mathscr {L}^1$ measure.

**Step 1.** In this first step we check that we can split in a Borel way each $\mu _t$ as a sum of mutually singular measures that are $\mathscr {H}^1$ restricted to the image of a bi-Lipschitz fragment. We claim that there exists a Borel map $(j,t)\mapsto \mu _{j,t}$ such that for almost every $t\in I$ we have

(i) $\mu _t=\sum _{j\in \mathbb {N}}\mu _{j,t}$ where  for some 2-bi-Lipschitz fragment $\gamma _{j,t}:K_{j,t}\rightarrow \mathbb {G}$;
(ii) $D\gamma _{j,t}(\gamma ^{-1}_{j,t}(x))\in \{\lambda D\gamma _t(s)\in V_1\setminus \{0\} :\lambda \in \mathbb {R}, \,s\in \gamma _{t}^{-1}(x)\}$ for every $j\in \mathbb {N}$ and for $\mu _{j,t}$-almost every $x\in \mathbb {G}$.

In order to prove items (i) and (ii) let us observe that [7, Lemma 4.1] implies that for every $t\in I$ there exists a family of 2-bi-Lipschitz maps $\tilde{\gamma }_{j,t}:K_{j,t}\rightarrow \mathbb {G}$ such that $\textrm{im}(\tilde{\gamma }_{j,t})$ are pairwise disjoint and

$$\begin{aligned} \mathscr {H}^1\Big (\textrm{im}(\gamma _t)\setminus \bigcup _{j\in \mathbb {N}} \textrm{im}(\tilde{\gamma }_{j,t})\Big )=0\qquad \text {and}\qquad \sum _{j\in \mathbb {N}}\mathscr {H}^1(\textrm{im}(\tilde{\gamma }_{j,t}))=\mathscr {H}^1(\textrm{im}(\gamma _t)). \end{aligned}$$

Denote by $\mathscr {B}$ the space of 2-bi-Lipschitz fragments and note that $\mathscr {B}$ is complete and separable when endowed with the metric $d_{eu,\mathscr {H}}$. Further, it is also immediate to see that $\mathscr {B}^\mathbb {N}$, the space of sequences in $\mathscr {B}$, is Polish. Thanks to (the proof of) Proposition A.3 we know that the map $\gamma \mapsto \Vert \llbracket \gamma \rrbracket \Vert$ is Borel. Hence, let $M:I\times \mathscr {B}^\mathbb {N}\rightarrow \mathscr {M}(\mathbb {R}^n,\mathbb {R})$ be the function defined as

Being a composition of Borel maps, it is immediate to see that *M* is Borel. Hence, the set $M^{-1}(0)$ is a Borel set and thus thanks to von Neumann measurable selection theorem, see [51, Theorem 5.5.2] we can find a universally measurable map $t\mapsto \{ \gamma _{j,t}\}_{j\in \mathbb {N}}$ such that $\mu _t=\sum _{j\in \mathbb {N}} \Vert \llbracket \gamma _{j,t}\rrbracket \Vert$. Summing up, so far we have shown that there exists a universally measurable map $\Psi :t\mapsto \{ \gamma _{j,t}\}_{j\in \mathbb {N}}$ such that

99 $$\begin{aligned} \mu _t=\sum _{j\in \mathbb {N}}\Vert \llbracket \gamma _{j,t}\rrbracket \Vert \qquad \text {for }\mathscr {L}^1\text {-almost every }t\in I. \end{aligned}$$

Clearly, by Lusin’s theorem there exists a Borel map that we will call with abuse of notations $\Psi$, still satisfying (99) and coinciding with the original $\Psi$ on a set of full $\mathscr {L}^1$ measure. The metric that makes $\mathscr {B}^\mathbb {N}$ a complete and separable metric space is

$$\begin{aligned} d_\infty (\{\gamma _j^1\}_{j\in \mathbb {N}},\{\gamma _j^2\}_{j\in \mathbb {N}}):=\sum _{j\in \mathbb {N}}2^{-j}\frac{d_{eu,\mathscr {H}}\left( \gamma _j^1,\gamma _j^2\right) }{1+d_{eu,\mathscr {H}}\left( \gamma _j^1,\gamma _j^2\right) }, \end{aligned}$$

and it can be easily checked that the projection on the *k*th component $\pi _k(\{\gamma _{j}\}_{j\in \mathbb {N}})=\gamma _k$ is continuous from $(\mathscr {B}^\mathbb {N},d_\infty )$ to $(\mathscr {B},d_{eu,\mathscr {H}})$. This implies that for every $j\in \mathbb {N}$ the map $t\mapsto \Vert \llbracket \gamma _{j,t}\rrbracket \Vert =\Vert \llbracket \pi _j(\{\gamma _k\}_{k\in \mathbb {N}})\rrbracket \Vert$ is composition of Borel maps and hence it is Borel. However, since $\mathbb {N}$ is countable, this also implies that $(j,t)\mapsto \Vert \llbracket \gamma _{j,t}\rrbracket \Vert$ is Borel. So, defined $\mu _{j,t}:=\Vert \llbracket \gamma _{j,t}\rrbracket \Vert$ we have by construction that for $\mathscr {L}^1$-almost every *t* we have

where the last identity follows from the fact that the curves $\gamma _{j,t}$ are biLipschitz. The above identity implies that the measures $\mu _{j,t}$ are mutually singular. If this was not the case there would be a Borel set of positive $\mathscr {H}^1$-measure where the Radon-Nykodim derivative of $\sum _{j\in \mathbb {N}}\mu _{j,t}$ with respect to $\mathscr {H}^1\llcorner \textrm{im}(\gamma _t)$ would be bigger than 2, which is excluded by the computation above. This concludes the proof of item (i).

Let us check item (ii). Thanks to [39, Theorem 4.4], we know that

$$\begin{aligned} \mathscr {H}^1(\textrm{im}(\gamma _t)\setminus \gamma _t({\tilde{K}}_t))=0, \end{aligned}$$

where ${\tilde{K}}_t:=\{s\in K_t:D\gamma _t(s)\ne 0\}$ and fix a $j\in \mathbb {N}$. It elementary to see that $\mu _t$-almost every $x\in \textrm{im}(\gamma _t)$ is a $\mu _t$ density point of $\textrm{im}(\gamma _{j,t})$. For such *x*’s can find $\sigma _0\in K_{j,t}$ and $\tau _0\in \tilde{K}_t$ such that the points $\sigma _0$ and $\tau _0$ are two Lebesgue density points for $K_{j,t}$ and ${\tilde{K}}_t$ respectively with $\gamma _{t}(\tau _0)=x$, $\gamma _{j,t}(\sigma _0)=x$ and such that $D\gamma _t(\tau _0)$ and such that $D\gamma _{j,t}(\sigma _0)$ exist; that $\tau _0$ is a $\mathscr {L}^1$ density point of the set $C_{j,t}:=\{s\in \tilde{K}_t:\gamma _t(s)\in \textrm{im}(\gamma _{j,t})\}$. In addition, for every $r\in C_{j,t}$ there exists $\sigma (r)\in K_{j,t}$ such that $\gamma _{j,t}(\sigma (r))=\gamma _t(r)$ and, since $\gamma _{j,t}$ is bi-Lipschitz, we infer that if $r\rightarrow \tau _0$ then $\sigma (r)\rightarrow \sigma _0$. By Pansu’s differentiability theorem, see Remark 2.5, we know that

100 $$\begin{aligned}&\lim _{C_{j,t}\ni s\rightarrow \tau _0+}d_\mathbb {G}(\delta _{|s-\tau _0|^{-1}}(\gamma _{t}(\tau _0)^{-1}*\gamma _{t}(s)),D\gamma _t(\tau _0))=0, \end{aligned}$$

101 $$\begin{aligned}&\lim _{C_{j,t}\ni s\rightarrow \tau _0+}d_\mathbb {G}\Big (\delta _{|\sigma (s)-\sigma _0|^{-1}}(\gamma _{j,t}(\sigma _0)^{-1}*\gamma _{j,t}(\sigma (s))),D\gamma _{j,t}(\sigma _0)\Big [\frac{\sigma (s)-\sigma _0}{|\sigma (s)-\sigma _0|}\Big ]\Big ) \nonumber \\&\qquad =\lim _{C_{j,t}\ni s\rightarrow \tau _0+}d_\mathbb {G}(\delta _{|\sigma (s)-\sigma _0|^{-1}}(\gamma _{j,t}(\sigma _0)^{-1}*\gamma _{j,t}(\sigma (s))),D\gamma _{j,t}(\sigma _0)\mathfrak {o}(s))=0, \end{aligned}$$

where in the second display equation above $\mathfrak {o}(s)$ denotes the sign of $\sigma (s)-\sigma _0$ and since $D\gamma _{j,t}(\sigma _0)$ is (or more precisely, can be represented by) a vector in the first layer of the algebra $V_1$, the element $D\gamma _{j,t}(\sigma _0)\mathfrak {o}(s)$ has to be intended as $D\gamma _{j,t}(\sigma _0)$ if $\mathfrak {o}(s)=1$ and as $-D\gamma _{j,t}(\sigma _0)$ if $\mathfrak {o}(s)=-1$. Note, further, that since $\gamma _{j,t}$ is 2-biLipschitz, we also know that

102 $$\begin{aligned} \frac{|\sigma (s)-\sigma _0|}{|s-\tau _0|}\le 2\frac{d_\mathbb {G}(\gamma _{j,t}(\sigma (s)),\gamma _{j,t}(\sigma _0))}{|s-\tau _0|}=2\frac{d_\mathbb {G}(\gamma _{t}(s),\gamma _{t}(\tau _0))}{|s-\tau _0|}\le 2, \end{aligned}$$

where the last inequality follows from the fact that $\gamma _t$ is assumed to be 1-Lipschitz. This implies in particular that

$$\begin{aligned} \begin{aligned} 0\overset{(100)}{=}&\lim _{C_{j,t}\ni s\rightarrow \tau _0+}d_\mathbb {G}(\delta _{|s-\tau _0|^{-1}}(\gamma _{t}(\tau _0)^{-1}*\gamma _{t}(s)),D\gamma _t(\tau _0))\\ =&\lim _{C_{j,t}\ni s\rightarrow \tau _0+}d_\mathbb {G}(\delta _{|s-\tau _0|^{-1}}(\gamma _{j,t}(\tau _0)^{-1}*\gamma _{j,t}(\sigma (s))),D\gamma _t(\tau _0))\\ =&\lim _{C_{j,t}\ni s\rightarrow \tau _0+}d_\mathbb {G}\Big (\delta _{\frac{|\sigma (s)-\sigma _0|}{|s-\tau _0|}}\Big (\delta _{|\sigma (s)-\sigma _0|^{-1}}\big (\gamma _{j,t}(\sigma _0)^{-1}*\gamma _{j,t}(\sigma (s))\big )\Big ),D\gamma _t(\tau _0)\Big )\\ \ge&\lim _{C_{j,t}\ni s\rightarrow \tau _0+}d_\mathbb {G}\Big (\delta _{\frac{|\sigma (s)-\sigma _0|}{|s-\tau _0|}}(D\gamma _{j,t}(\sigma _0)\mathfrak {o}(s)),D\gamma _t(\tau _0)\Big )\\&\quad -\lim _{C_{j,t}\ni s\rightarrow \tau _0+}\frac{|\sigma (s)-\sigma _0|}{|s-\tau _0|}d_\mathbb {G}\Big (\delta _{|\sigma (s)-\sigma _0|^{-1}}\big (\gamma _{j,t}(\sigma _0)^{-1}*\gamma _{j,t}(\sigma (s))\big ),D\gamma _{j,t}(\tau _0)\Big )\\ \overset{(101),\,(102)}{=}&\lim _{C_{j,t}\ni s\rightarrow \tau _0+}d_\mathbb {G}\Big (\delta _{\frac{|\sigma (s)-\sigma _0|}{|s-\tau _0|}}(D\gamma _{j,t}(\sigma _0)\mathfrak {o}(s)),D\gamma _t(\tau _0)\Big ). \end{aligned} \end{aligned}$$

Since both $D\gamma _{j,t}(\sigma _0)$ and $D\gamma _t(\sigma _0)$ are contained in the first layer $V_1$, we know that the above identity implies that

$$\begin{aligned} \lim _{C_{j,t}\ni s\rightarrow \tau _0+}\frac{|\sigma (s)-\sigma _0|\mathfrak {o}(s)}{|s-\tau _0|}D\gamma _{j,t}(\sigma _0)=D\gamma _t(\tau _0). \end{aligned}$$

In particular this shows that $\frac{|\sigma (s)-\sigma _0|\mathfrak {o}(s)}{|s-\tau _0|}$ converges to some $\lambda \in \mathbb {R}$ and hence item (ii) is proved.

**Step 2.** In the next step we show that the map that associates to each point $x\in \mathbb {G}$ and to any bi-Lipschitz fragment $\gamma _{j,t}$ the Radon-Nykodym derivative of $\llbracket \gamma _{j,t}\rrbracket$ with respect to $\Vert \llbracket \gamma _{j,t}\rrbracket \Vert$ at $x\in \mathbb {G}$ is Borel. Thanks to Lemma A.3(ii) there is a Borel map $\Gamma :\mathbb {N}\times I\rightarrow \mathfrak {F}$ such that $(j,t)\mapsto \llbracket \Gamma (j,t)\rrbracket$ is Borel and $\Vert \llbracket \Gamma (j,t)\rrbracket \Vert =\mu _{j,t}$ for $\mathscr {H}^0\otimes \mathscr {L}^1$-almost every $(j,t)\in \mathbb {N}\times I$. Concerning the applicability of Lemma A.3(ii) to the above situation, we note that the statements in Lemma A.3 requires that the $\mu _t$’s are parametrized by a Borel subset of $\mathbb {R}$, however, either by skimming the proof of Lemma A.3, or by recalling that we can reparametrize $(\mathbb {N}\times I,\mathscr {H}^0\otimes \mathscr {L}^1)$ as $(\mathbb {R},\mathscr {L}^1)$ by [3, Remark 2.7 (iii)], we see that Lemma A.3(ii) is still applicable.

Let us denote by $I_0$ a Borel subset of *I* of full measure such that $\Vert \llbracket \Gamma (j,t)\rrbracket \Vert =\mu _{j,t}$ on $\mathbb {N}\times I_0$ and let us note that $(j,t)\mapsto \delta _j\otimes \delta _t\otimes \mu _{j,t}$ is Borel on $\mathbb {N}\times I_0$. To see this, it is sufficient to check that for every Borel set $G\subseteq \mathbb {N}\times I_0\times \mathbb {R}^n$ the map $(j,t)\mapsto \delta _j\otimes \delta _t\otimes \mu _{j,t}(G)$ is Borel, where $\delta _\iota$ and $\delta _s$ denote the Dirac deltas at $\iota$ and *s* respectively, compare with Definition 2.11. Note that

$$\begin{aligned} \delta _j\otimes \delta _t\otimes \mu _{j,t}(G)= &   \int \int \int \mathbb {1}_G(k,s,x)d\delta _j(k)d\delta _t(s)d\mu _{j,t}(x)\\= &   \mu _{j,t}(\{x\in \mathbb {G}:(j,t,x)\in G\})\quad \text {for every }(j,t)\in \mathbb {N}\times I_0. \end{aligned}$$

Since $\mathbb {N}$ is countable, to check that $(j,t)\mapsto \delta _j\otimes \delta _t\otimes \mu _{j,t}(G)$ is Borel, it is clearly enough to prove that for every fixed $k\in \mathbb {N}$ we have that $t\mapsto \mu _{k,t}(\{x\in \mathbb {G}:(k,t,x)\in G\})$ is Borel. This however can be proved arguing, mutatis mutandis, as in the step *construction and Borelianity of* $\Psi$ of the proof of Lemma A.3. The fact that the map $(j,t)\mapsto \delta _j\otimes \delta _t\otimes \mu _{j,t}$ is Borel implies that we can therefore define the measure

$$\begin{aligned} \nu :=\int _{\mathbb {N}\times I_0} \delta _\iota \otimes \delta _s\otimes \mu _{\iota ,s}\,d\mathscr {H}^0(\iota ) ds. \end{aligned}$$

It is immediate to see that the set

$$\begin{aligned} H_1:=\{(j,t,x)\in \mathbb {N}\times I_0\times \mathbb {G}:x\in \textrm{im}(\gamma _{j,t})\text { and }\gamma _{j,t}'(\gamma _{j,t}^{-1}(x))\text { exists}\}, \end{aligned}$$

is a set of full $\nu$-measure and therefore it coincides up to sets of $\nu$-null measure with a Borel set $H_2$. Further, define the map

$$\begin{aligned} \gimel _\ell (j,t,x):=\frac{\llbracket \Gamma (j,t)\rrbracket (U(x,\ell ^{-1}))}{\mu _{j,t}(U(x,\ell ^{-1}))}=\frac{\textsf{E}_{\ell ^{-1}}^{n,n}( \llbracket \Gamma (j,t)\rrbracket ,x)}{\textsf{E}_{\ell ^{-1}}^{n,1}(\mu _{j,t},x)}, \end{aligned}$$

where $\textsf{E}_{\ell ^{-1}}^{n,n}$ and $\textsf{E}_{\ell ^{-1}}^{n,1}$ are the Borel map introduced in Lemma A.5. Observe that since $\gimel _\ell$ is composition of Borel functions (on $H_2$), it is Borel as well. By Besicovitch-Lebesgue differentiation theorem, see [6, Theorem 2.22], for every $j\in \mathbb {N}$ and every $t\in I_0$ we have that

$$\begin{aligned} \frac{d\llbracket \Gamma (j,t)\rrbracket }{d\mu _{j,t}}(x)= &   \lim _{r\rightarrow 0}\frac{\llbracket \Gamma (j,t)\rrbracket (U(x,r))}{\mu _{j,t}(U(x,r))}=\lim _{\ell \rightarrow \infty }\frac{\llbracket \Gamma (j,t)\rrbracket (U(x,\ell ^{-1}))}{\mu _{j,t}(U(x,\ell ^{-1}))}\\= &   \lim _{\ell \rightarrow \infty }\gimel _\ell (j,t,x)\qquad \text {for }\mu _{j,t}\text {-almost every } x\in \mathbb {G}. \end{aligned}$$

This shows that on Borel a subset of full $\nu$-measure $H_3$ of $H_2$, the function $(j,t,x)\mapsto \frac{d\llbracket \Gamma (j,t)\rrbracket }{d\mu _{j,t}}(x)$ coincides with a Borel function, being pointwise limit of a sequence of Borel functions. In addition, thanks to the definition of $\llbracket \Gamma (j,t)\rrbracket$, Lebesgue differentiation theorem implies that for every $j\in \mathbb {N}$ and $t\in I_0$ we have

103 $$\begin{aligned} \frac{d\llbracket \Gamma (j,t)\rrbracket }{d\mu _{j,t}}(x)=\gamma _{j,t}^\prime (\gamma _{j,t}^{-1}(x))\in H\mathbb {G}(x)\setminus \{0\}, \qquad \text { for }\mu _{j,t}\text {-almost every } x\in \mathbb {G}. \nonumber \\ \end{aligned}$$

Summing up, this shows that we can find a Borel subset $H_4$ of full $\nu$-measure in $\mathbb {N}\times I_0\times \mathbb {G}$ such that for every $(j,t,x)\in H_4$ we have

- $$x\in \textrm{im}(\gamma _{j,t})$$;
- $\gamma _{j,t}^\prime (\gamma _{j,t}^{-1}(x))\in H\mathbb {G}(x)\setminus \{0\}$ exists and $\frac{d\llbracket \Gamma (j,t)\rrbracket }{d\mu _{j,t}}(x)=\gamma _{j,t}^\prime (\gamma _{j,t}^{-1}(x))$ and $(j,t,x)\mapsto \frac{d\llbracket \Gamma (j,t)\rrbracket }{d\mu _{j,t}}(x)$ is Borel on $H_4$.

Therefore, we define on $\mathbb {N}\times I\times \mathbb {G}$ and on $I\times \mathbb {G}$ respectively the maps

$$\begin{aligned} \tilde{\mathfrak {d}}(j,t,x):={\left\{ \begin{array}{ll} \frac{d\llbracket \Gamma (j,t)\rrbracket }{d\mu _{j,t}}(x) & \text {if }(j,t,x)\in H_4\\ 0& \text {otherwise} \end{array}\right. }\qquad \text {and}\qquad \mathfrak {d}(t,x):=\sum _{j\in \mathbb {N}} \tilde{\mathfrak {d}}(j,t,x). \end{aligned}$$

Both $\tilde{\mathfrak {d}}$ and $\mathfrak {d}$ are immediately seen to be Borel. However, since $\mu _{j_1,t}$ and $\mu _{j_2,t}$ are mutually singular whenever $j_1\ne j_2$, we infer that

104 $$\begin{aligned} \mathfrak {d}(t,x)= &   \tilde{\mathfrak {d}}(j,t,x) \qquad \text {for } \mu _{j,t}\text {-almost every }x\in \mathbb {G}, \nonumber \\  &   \text { for }\mathscr {L}^1\text {-almost every }t\text { and every }j\in \mathbb {N}. \end{aligned}$$

This immediately implies by item (i) of step 1 that we also have that $\mathfrak {d}(t,x)\in H\mathbb {G}(x)\setminus \{0\}$ for $\mu _t$-almost every $x\in \mathbb {R}^n$ and $\mathscr {L}^1$-almost every *t*.

**Step 3.** In this last step we introduce the map $\mathfrak {v}$ and conclude the proof. Define

$$\begin{aligned} \mathfrak {v}(t,x):={\left\{ \begin{array}{ll} \frac{\pi _1(\mathfrak {d}(t,x))}{|\pi _1(\mathfrak {d}(t,x))|}& \text { if }\mathfrak {d}(t,x)\ne 0;\\ 0& \text {otherwise}. \end{array}\right. }, \end{aligned}$$

and note that $\mathfrak {v}$ is immediately seen Borel. Since $\mathfrak {d}(t,x)=\gamma _{j,t}'(\gamma _{j,t}^{-1}(x))$ on $(j,t,x)\in H_4$, we infer that for those (*t*, *x*) for which there exists $j\in \mathbb {N}$ such that $(j,t,x)\in H_4$ we have

105 $$\begin{aligned} \mathfrak {v}(t,x)= &   \frac{\pi _1(\mathfrak {d}(t,x))}{|\pi _1(\mathfrak {d}(t,x))|}=\frac{\pi _1(\gamma _{j,t}'(\gamma _{j,t}^{-1}(x)))}{|\pi _1(\mathfrak {d}(t,x))|}\nonumber \\= &   \frac{D\gamma _{j,t}(\gamma _{j,t}^{-1}(x))}{|\pi _1(\mathfrak {d}(t,x))|}\in \{\lambda D\gamma _t(s)\in V_1\setminus \{0\} :\lambda \in \mathbb {R}, \,s\in \gamma _{t}^{-1}(x)\}, \end{aligned}$$

where in the first identity above we used the definition of $\mathfrak {v}$; in the second identity we have employed item (b) of the properties of $H_4$ in step 2, (104) and the definition of $\mathfrak {d}$; in the last identity we used Lemma 2.6 and Lemma 2.7 that imply that $D\gamma =\pi _1(\gamma ')$; finally in the inclusion we have used item (ii) of step 1. The set $\tilde{E}:=\bigcup _{j\in \mathbb {N}}\{(t,x)\in I\times \mathbb {G}:(j,t,x)\in H_4\}$ is Borel and he have therefore proved above that

$$\begin{aligned}  &   \mathfrak {v}(t,x)\in \{\lambda D\gamma _t(s)\in V_1\setminus \{0\} :\lambda \in \mathbb {R}, \,s\in \gamma _{t}^{-1}(x)\}\\  &   \qquad \text {for every }(t,x)\in \tilde{E}. \end{aligned}$$

However, since $H_4$ has full $\nu$-measure it is elementary to check that $\tilde{E}$ has full $\int \delta _s\otimes \mu _s\, ds$-measure and this shows in particular that

$$\begin{aligned}  &   \mathfrak {v}(t,x)\in \{\lambda D\gamma _t(s)\in V_1\setminus \{0\} :\lambda \in \mathbb {R}, \,s\in \gamma _{t}^{-1}(x)\}\\  &   \qquad \text {for } \mu _{t}\text {-almost every }x\in \mathbb {G}\text { for almost every }t\in I, \end{aligned}$$

concluding the proof. $\square$

## Differentiation Properties for Non-doubling Radon Measures

In this section we prove that for every Radon measure $\nu$ and $\nu$-almost everywhere there exists a sequence of scales on which $\nu$ behaves like a doubling measure and along such sequence of scales a Lebesgue differentiation type theorem holds. The following Lemma is a Carnot analogue of [47, Lemma 2.4].

### Lemma B.1

Let $\nu$ be a non-negative Radon measure on $\mathbb {G}$ and let $t \in (0,1)$. Then, for every $j\in \mathbb {N}$ the set

$$\begin{aligned} E_{\nu ,j}^t:=\Big \{x\in \mathbb {R}^n:\limsup _{k\rightarrow \infty }\frac{\nu (B(x,t^{k+1}))}{\nu (B(x,t^k))}>j^{-1}\Big \}, \end{aligned}$$

is Borel. In addition $\nu (\mathbb {R}^n\setminus E_{\nu }^t)=0$, where $E_{\nu }^t=\cup _{j\in \mathbb {N}}E_{\nu ,j}^t$.

### Proof

Since the map $x\mapsto \nu (B(x,t^k))$ is upper semicontinuous, we infer that

$$\begin{aligned} \mathfrak {r}(x):=\limsup _{k\rightarrow \infty }\frac{\nu (B(x,t^{k+1}))}{\nu (B(x,t^k))}\qquad \text {is Borel.} \end{aligned}$$

This shows that the set $E_{\nu ,j}^t=\{x\in \mathbb {R}^n:\mathfrak {r}(x)>j^{-1}\}$ is Borel as well. Denote $\mathscr {B}:=\mathbb {R}^n\setminus \cup _{j\in \mathbb {N}}E_{\nu ,j}^t$ and assume by contradiction that $\nu (\mathscr {B})>0$. Since as seen above, the functions $x\mapsto \nu (B(x,t^{k+1}))/\nu (B(x,t^k))$ are Borel, by Severini-Egorov’s Theorem there exists a compact set $K\subset \mathscr {B}$ with $\nu (K)>0$ where for every $\varepsilon >0$ there exists $k_0\in \mathbb {N}$ such that

$$\begin{aligned} {\nu (B(x,t^{k+1}))} \le \varepsilon {\nu (B(x,t^k))}\qquad \text {for every } x\in K \text { and every } k\ge k_0. \end{aligned}$$

For every such $x\in K$ it therefore holds that

$$\begin{aligned} \nu (B(x,t^{k})) \le \nu (B(x,t^{k_0})) \varepsilon ^{k-k_0}\qquad \text {whenever }k\ge k_0. \end{aligned}$$

Since *K* is compact, $\nu$ is Radon and $t<1$, there exists a constant $C>0$ such that $\sup _{x\in K}\nu (B(x,t^{k_0}))\le C$. Therefore, thanks to Remark 2.2 there exists a constant $\vartheta \in (0,1)$ possibly depending on *K* such that

$$\begin{aligned} \nu (U(x, \vartheta t^{\mathfrak {s}k})) \le C \varepsilon ^{k-k_0}\qquad \text {whenever }k\ge k_0, \end{aligned}$$

where *U*(*x*, *r*) denotes the closed euclidean ball with centre *x* and radius *r*. If we let $\delta \in (0,1)$ and $\varepsilon :=\delta t^{\mathfrak {s}n}$, there exists $k_0=k_0(\delta ,\vartheta ,t)$ such that

$$\begin{aligned} \nu (U(x, \vartheta t^{\mathfrak {s}k})) \le C \delta ^{k-k_0}t^{\mathfrak {s}n(k-k_0)}=C \delta ^{k-k_0}t^{-\mathfrak {s}nk_0}\vartheta ^{-n}(\vartheta t^{\mathfrak {s}k})^n\qquad \text {whenever }k\ge k_0. \end{aligned}$$

Let $k_1\ge k_0+2$ and $\mathcal {U}:=\{U(x,\vartheta t^{\mathfrak {s}k}):x\in K\text { and }k\ge k_1\}$ and note that, thanks to Besicovitch’s covering theorem, (see [22, Corollary 2.8.15]), there exists a countable disjoint subfamily $\tilde{\mathcal {U}}=\{U(x_i,r_{i})\}$ of $\mathcal {U}$ such that $\nu (K\setminus \cup _{i\in \mathbb {N}}B(x_i,r_i))=0$. This implies that

$$\begin{aligned} \begin{aligned} \nu (K)=&\sum _{i\in \mathbb {N}} \nu (B(x_i,\vartheta t^{\mathfrak {s}k_i})) \le C t^{-\mathfrak {s}nk_0}\vartheta ^{-n}\sum _{i\in \mathbb {N}}\delta ^{k_i-k_0} (\vartheta t^{\mathfrak {s}k_i})^n \\ \le&C\delta ^{k_1-k_0}\vartheta ^{-n} t^{-\mathfrak {s}nk_0}\sum _{i\in \mathbb {N}} (\vartheta t^{\mathfrak {s}k_i})^n\\ =&C\mathscr {L}^n(B(0,1))^{-1}\delta ^{k_1-k_0}\vartheta ^{-n} t^{-\mathfrak {s}nk_0}\sum _{i\in \mathbb {N}}\mathscr {L}^n(U(x_i,\vartheta t^{\mathfrak {s}k_i})) \\ \le&C\mathscr {L}^n(B(0,1))^{-1}\delta ^{k_1-k_0}\vartheta ^{-n} t^{-\mathfrak {s}nk_0} \mathscr {L}^1(U(K,1)). \end{aligned} \end{aligned}$$

where *U*(*K*, 1) denotes the closed Euclidean neighbourhood of radius 1 of the compact set *K*. Since $\delta \in (0,1)$ the arbitrariness of $k_1$ implies that $\nu (K)=0$ and in turn $\nu (\mathscr {B})=0$. This is a contradiction. $\square$

### Proposition B.2

Let $\nu$ be a Radon measure on $\mathbb {G}$ and let $f\in L^1(\nu )$. Then, for $\nu$-almost every $x\in \mathbb {G}$ there exists an infinitesimal sequence $r_k^x$ such that

106

In addition, if we let $\nu =\nu ^a+\nu ^s$ be the Radon-Nikodym decomposition of $\nu$ where $\nu ^a\ll \mathscr {L}^n$ and $\nu ^s\perp \mathscr {L}^n$, we have that

$$\begin{aligned} \lim _{k\rightarrow \infty } \frac{\nu ^a(B(x,r_k^x))}{\nu ^s(B(x,r_k^x))}=0\qquad \text {for } \nu ^s\text {-almost every }x\in \mathbb {G}. \end{aligned}$$

### Proof

Let us fix $t:=1/5$, and note that thanks to Lemma B.1 it is clearly sufficient to prove the proposition for $x\in E_{\nu ,j}^t$. Let $j\in \mathbb {N}$ and

$$\begin{aligned} \mathscr {U}_j:=\{B(x,t^k):x\in E_{\nu ,j}^t\text { and }\nu (B(x,t^{k}))\ge j^{-1}\nu (B(x,t^{k+1}))\}, \end{aligned}$$

The covering $\mathscr {U}_j$ is fine at every $x\in E_{\nu ,j}^t$ and, applying [22, Theorem 2.8.17] with the choice $\delta :=\textrm{diam}(\cdot )$ to $\mathscr {U}_j$ we see that $\mathscr {U}_j$ is a $\nu$-Vitali covering of $E_{\nu ,j}^t$, see [22, §2.8.16]. Therefore, by [22, Corollary 2.9.9] we infer that for every $\varphi \in L^1_{loc}(\nu )$ we have

for $\nu$-almost every $x\in E_{\nu ,j}^t$. Finally, since $\nu ^a$ and $\nu ^s$ are mutually singular, there exists a Borel set *E* such that $\nu ^s(\mathbb {G}\setminus E)=0$ and $\nu ^a(E)=0$. This implies in particular that for $\nu$-almost every $x\in E\cap E_{\nu ,j}^t$ we have

$$\begin{aligned} 0= &   \lim _{\begin{array}{c} k\rightarrow \infty \\ B(x,t^{k})\in \mathscr {U}_j \end{array}}\frac{\nu (B(x,t^k)\setminus E\cap E_{\nu ,j}^t)}{\nu (B(x,t^k))}=1-\lim _{\begin{array}{c} k\rightarrow \infty \\ B(x,t^{k})\in \mathscr {U}_j \end{array}}\frac{\nu (B(x,t^{k})\cap E\cap E^t_{\nu ,j})}{\nu (B(x,t^{k}))}\\= &   1-\lim _{\begin{array}{c} k\rightarrow \infty \\ B(x,t^{k})\in \mathscr {U}_j \end{array}}\frac{\nu ^s(B(x,t^{k})\cap E^t_{\nu ,j})}{\nu (B(x,t^{k}))}. \end{aligned}$$

The above computation shows in particular that

$$\begin{aligned} \lim _{\begin{array}{c} k\rightarrow \infty \\ B(x,t^{k})\in \mathscr {U}_j \end{array}}\frac{\nu ^a(B(x,t^{k}))}{\nu (B(x,t^{k}))}=0\qquad \text {for }\nu \text {-almost every }x\in E\cap E_{\nu ,j}^t, \end{aligned}$$

that in turn immediately implies that

$$\begin{aligned} \lim _{\begin{array}{c} k\rightarrow \infty \\ B(x,t^{k})\in \mathscr {U}_j \end{array}}\frac{\nu ^a(B(x,t^{k}))}{\nu ^s(B(x,t^{k}))}=0,\qquad \text {for } \nu ^s\text {-almost every }x\in E_{\nu ,j}^t. \end{aligned}$$

This concludes the proof. $\square$
